# The odorant receptor repertoire of teleost fish

**DOI:** 10.1186/1471-2164-6-173

**Published:** 2005-12-06

**Authors:** Tyler S Alioto, John Ngai

**Affiliations:** 1 Department of Molecular and Cell Biology, Functional Genomics Laboratory, Helen Wills Neuroscience Institute, University of California, Berkeley, California 94720, USA; 2 Grup de Recerca en Informàtica Biomèdica, Institut Municipal d'Investigació Mèdica, Universitat Pompeu Fabra, Centre de Regulació Genòmica, Psg. Marítim 37-49, 08003 Barcelona, Spain

## Abstract

**Background:**

Vertebrate odorant receptors comprise three types of G protein-coupled receptors: the OR, V1R and V2R receptors. The OR superfamily contains over 1,000 genes in some mammalian species, representing the largest gene superfamily in the mammalian genome.

**Results:**

To facilitate an informed analysis of *OR *gene phylogeny, we identified the complete set of 143 *OR *genes in the zebrafish genome, as well as the OR repertoires in two pufferfish species, fugu (44 genes) and tetraodon (42 genes). Although the genomes analyzed here contain fewer genes than in mammalian species, the teleost *OR *genes can be grouped into a larger number of major clades, representing greater overall OR diversity in the fish.

**Conclusion:**

Based on the phylogeny of fish and mammalian repertoires, we propose a model for *OR *gene evolution in which different ancestral *OR *genes or gene families were selectively lost or expanded in different vertebrate lineages. In addition, our calculations of the ratios of non-synonymous to synonymous codon substitutions among more recently expanding OR subgroups in zebrafish implicate residues that may be involved in odorant binding.

## Background

The perception and discrimination of thousands of different odorants by the vertebrate olfactory system results from the activation of specific odorant receptors expressed by olfactory neurons in the nose. The first odorant receptors were identified in the rat [1] and belong to what is now referred to as the "OR" superfamily of odorant receptors [2]. ORs exhibit a predicted seven transmembrane topology and sequence motifs characteristic of the A family (rhodopsin-like or Class I) of G protein-coupled receptors. Subsequent to the initial discovery of the OR superfamily of odorant receptors, two unrelated types of G protein-coupled receptors (GPCRs) were identified in the mammalian vomeronasal organ, the V1R receptors [3] and the V2R receptors [4-7]. The vomeronasal V1R and V2R receptors are thought to subserve signaling to pheromonal compounds [2].

The *OR *gene superfamily is the largest multigene superfamily described in mammalian genomes. The completion of both the Celera and public consortium versions of the mouse genome confirmed the existence of about 1068 potential intact *OR *genes (comprising at least 228 subfamilies) and 334 pseudogenes [8,9]. In humans, there are ~340 intact *OR *genes and ~300 pseudogenes [10-12]. By way of contrast, molecular cloning and genomic DNA blot hybridizations in fish species suggest an *OR *repertoire size approximately five- to ten-fold smaller than that of mammalian species [13,14].

An understanding of how vertebrate olfactory receptor repertoires evolved can be gained from comparing the properties and organization of genes from divergent vertebrate species. In this regard, the zebrafish, *Danio rerio*, provides a useful model for comparative genomics studies. Recent studies have demonstrated that the zebrafish genome encodes only 1 *V1R*-like receptor [15] (T.S.A and J.N., unpublished results) and ~60 olfactory C-family (Class III) GPCRs related to the mammalian V2R family (T.S.A., P. Luu, E. VanName, and J.N., manuscript in preparation); one fish olfactory C-family receptor has been shown to be activated by amino acids [16,17], which are potent odorants for fish.

In the OR superfamily, approximately 28 genes and 5 pseudogenes were identified previously in zebrafish using PCR and homology-based techniques [14,18-22]. Although a number of phylogenetic reconstructions have been made [8,9,18,23-28], a more accurate view of the *OR *superfamily's evolutionary history would be facilitated by comparisons between genomic datasets that include a more complete representation of member genes from each species (see also [29]).

For the present study, we carried out genome data mining on the zebrafish genome sequence provided by the Sanger Institute *Danio rerio *Sequencing Project and found 143 potentially intact genes belonging to the zebrafish OR superfamily. We find that despite the limited size of the zebrafish OR repertoire, it comprises eight diverse OR families, with family members sharing on average ~40% amino acid identity. In addition, *OR *genes from two pufferfish species – fugu and tetraodon – can be grouped into six families which overlap with the zebrafish gene families. Analysis of the ratio of possible non-synonymous to synonymous codon substitutions suggests that OR genes in general are under negative or purifying selection; only a small number of residues within the transmembrane domains – the likely sites of odorant binding – appear to have undergone positive selection. Based on these findings we propose a model for the evolution of the vertebrate OR repertoire.

## Results and discussion

### Prediction of zebrafish OR genes

The third (Zv3) and fourth (Zv4) draft zebrafish genome assemblies  of whole genome shotgun sequence (5.7 × coverage) were searched for *OR *gene sequences using a modification of the method described for identifying *OR *sequences from the mouse genome [8].

The protein coding sequences of the vast majority of known *OR *genes characterized to date are uninterrupted by introns, which obviates the need for splice site prediction in the identification of most *OR *genes. Our gene prediction strategy was to combine a low-threshold BLAST search with profile Hidden Markov Model- (HMM) based gene prediction with the program Genewise . The Genewise results were post-processed using custom Perl scripts to generate complete ORFs. This process was repeated in an iterative fashion, as follows. The zebrafish genome assembly was subjected to TBLASTN search with a representative set of known zebrafish *OR*s (<50% percent identity among members of this set). The gene prediction program Genewise was then run on the genomic sequences surrounding each unique BLAST hit using a profile hidden Markov model (HMM) of the *OR *superfamily (see Methods for details). In each round, the newly predicted *OR *genes were added as queries for the next BLAST search. They were also aligned to previous members, and a new profile HMM was constructed for use in the next round of gene prediction.

Initial query sequences included members of each of the *OR9*, *OR2*, *OR5*, *OR4*, and *OR13 *subfamilies described previously [18]. Predicted genes were subjected to a set of criteria for inclusion in the final set of *OR *genes. First, all coding sequences were required to be longer than 700 base pairs in length and show highest sequence similarity to previously characterized *OR *sequences. To be considered full-length, the deduced amino acid sequence was required to be greater than 275 amino acids, contain seven predicted transmembrane domains and exhibit the presence of a conserved N-linked glycosylation site with the pattern N-X- [TS]-X (where X is any amino acid residue except for proline) at the N-terminus. Sequences failing to meet these criteria and those lacking start or stop codons due to assembly gaps were considered to be partial genes. FASTY comparison to known OR peptide sequences was used to generate conceptual translations of potential pseudogenes and to determine the number of disruptions (frame shifts, early stop codons and in-frame deletions). To compensate for disruptions in OR coding sequences due to possible sequencing and/or assembly errors, we adopted a classification system previously proposed for mining of the mouse genome [8]. In the present study, a gene was considered intact if it had a complete coding sequence with up to one disruption, or a pseudogene if it was either a partial sequence with one or more disruptions or a full-length sequence with two or more disruptions.

Based on these criteria, our search identified 143 intact *OR *genes (136 with no disruptions), 7 partial genes, 10 pseudogenes greater than 700 bp in length, and 15 gene fragments shorter than 700 bp (these shorter fragments were excluded from further analysis) [see Additional file 2 and Additional file 10]. Thus, we believe our total *OR *gene count is a conservative estimate of the true size of the OR repertoire, with between 78% (136/175) and 86% ([143+7]/175) of identifiable OR sequences consisting of potentially functional *OR *genes.

How complete is the predicted *OR *gene repertoire? To address this question, we extracted 65 zebrafish *OR *sequences (28 non-redundant published *OR *genes or cDNAs, 4 unpublished full-length cDNAs, and 33 ESTs) from Genbank and determined whether or not they were represented in the set of predicted genes by aligning them to OR genomic sequence. The 33 ESTs were first consolidated into 23 clusters based on overlapping sequences. Eight of these corresponded to known genes, while 15 clusters were novel, bringing the non-redundant set of Genbank genes to 47. Forty-five out of 47 of these sequences were identified in our search [see Additional file 10]. The two that we were unable to identify, *OR115*-*15 *and *OR128*-*14 *(an unpublished cDNA that was identified in the more recent Zv5 genome assembly and an EST, respectively) belong to two of the largest subfamilies in the repertoire. Thus, we are confident that the repertoire of odorant receptors described here includes nearly all of the *OR *genes encoded in the zebrafish genome. However, because some gaps remain in the assembly near several small receptor gene clusters, we expect that as the genome sequence is refined, a few additional receptor genes will be found.

### OR nomenclature and classification

OR families are typically defined as monophyletic groups with members that share greater than 40% amino acid identity, whereas subfamily members share greater than 60% amino acid identity [30]. Using these operational definitions, we classified the zebrafish *OR *genes into families and subfamilies by reconstructing their phylogeny by neighbor-joining with 1000 bootstrap replicates. Clades of *OR *genes with less than ~40% and less than ~60% inter-branch amino acid identity were used to group genes into distinct families and subfamilies, respectively. The average percent identity between families is approximately 25% while the maximum observed percent identity between any two ORs of different families is 39%.

To unify the naming for zebrafish *OR *genes, we propose a revised nomenclature based on the following rationale. Both newly predicted and previously described *OR *genes were named (or re-named) according to subfamily membership. Subfamilies were numbered sequentially starting at the number 101 (to avoid confusion with previous zebrafish *OR *nomenclature) in a depth-first traversal of the phylogenetic tree. Within subfamilies, *OR*s were numbered sequentially according to genomic position, if known. The new nomenclature showing subfamily membership and correspondence to previously identified zebrafish *OR *genes are shown in Table S1 [see Additional file 10].

### Genomic distribution of zebrafish OR genes

**Figure 1:**
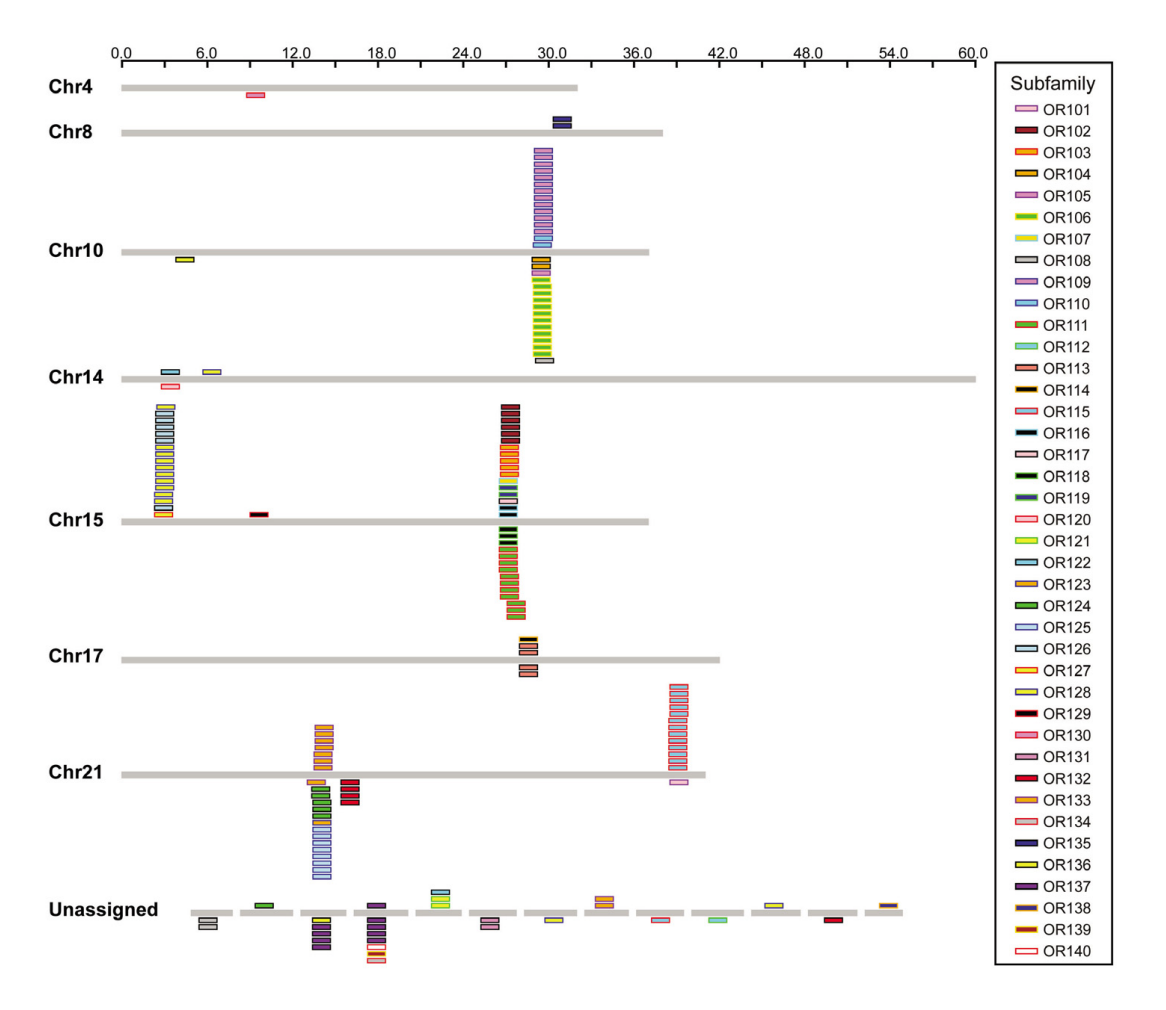
**Chromosomal distribution of zebrafish *OR *genes**. The majority of OR genes are organized in large clusters at only a few loci in the zebrafish genome. OR genes are depicted as boxes above (plus strand) or below (minus strand) a line representing each chromosome that encodes ORs. Genes are color-coded according to subfamily.

Previous studies have demonstrated that *OR *genes are clustered in vertebrate genomes [8,9,18,31,32]. In mammalian genomes, *OR *genes are distributed widely, residing on 18 chromosomes in the mouse [8] and 21 chromosomes in humans [10,11]. From the zebrafish Zv3 and Zv4 assemblies, we found that 119 of the identified zebrafish *OR *genes are distributed in five major clusters containing between 14 and 31 genes each. There are two clusters on chromosome 15, two on chromosome 21, one on chromosome 10, several small clusters on chromosomes 8, 14 and 17, and in a few cases, genes exist as singletons (Figure 1) [see Additional file 10]. Subfamilies are largely contiguous (see below) and subfamily members usually share the same transcriptional orientation, suggesting tandem duplication as a mechanism of expansion within a subfamily [18,33]. We were able to assign genomic locations for ~80% of the *OR *genes we identified (29 remain unassigned).

### Phylogeny of zebrafish OR genes

**Figure 2:**
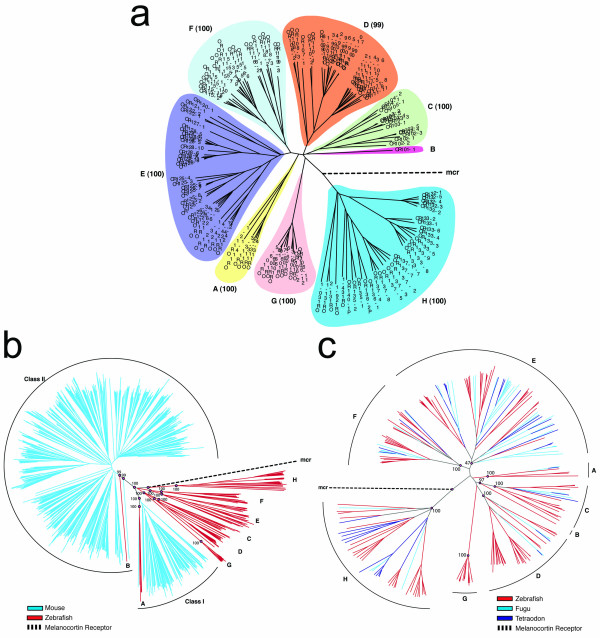
**Phylogeny of zebrafish and other vertebrate OR families**. **(a) **Phylogeny of zebrafish receptors. A neighbor joining tree was constructed based on an alignment of the predicted amino acid sequences of 143 intact genes and 4 full-length pseudogenes identified from the zebrafish genome [see Additional file 2]. *OR *genes are named by subfamily and colored by family. The eight gene families are labeled A-H. The zebrafish melanocortin receptor branch (dotted line labeled "mcr") indicates the root of the tree. Bootstrap scores for each family are indicated in parentheses. **(b) **Phylogenetic relationship among zebrafish and mouse odorant receptors. The following sets of genes were aligned and used to construct a tree by neighbor joining: the mouse odorant receptors, mORs [8,9]; the subset of 136 intact zebrafish ORs with no disruptions identified in this study (highlighted in red); and mouse melanocortin receptors (mcr). Note the presence of *OR *gene subfamilies *OR112*, *OR113*, and *OR114 *(Family A) within the Class I clade and zebrafish *OR101*-*1 *(Family B) within the Class II clade. Bootstrap scores corresponding to selected nodes are indicated.**(c) **Phylogeny of the complete OR repertoires of zebrafish, fugu and tetraodon identified in this study. One-hundred-thirty-six non-disrupted genes from zebrafish, 42 non-disrupted genes from fugu, and 42 non-disrupted genes from tetraodon were used in this neighbor joining analysis. Families are labeled A-H and correspond to the zebrafish families shown in panel A. Bootstrap scores corresponding to selected nodes are indicated.

Using a neighbor joining algorithm (see Methods), we constructed a phylogenetic tree of the 143 intact *OR *genes and 4 full-length pseudogenes identified in the two zebrafish genome assemblies, using the zebrafish melanocortin receptors as an outgroup (Figure 2a). Based on this analysis and the criteria set forth above, zebrafish ORs could be classified into 8 families (≥40% intra-family sequence identity) and 40 subfamilies (≥60% intra-subfamily sequence identity). An additional thirteen sequences comprising 7 partial genes and 6 pseudogene fragments were subsequently assigned to subfamilies based on their sequence similarities and additional phylogenetic analyses (data not shown). Most of the gene families contain between 12 and 40 genes each; the two smallest families, Family A and Family B, contain 6 and 1 genes each, respectively. The intra-subfamily identity threshold was lowered for three subfamilies, OR102, OR115 and OR125, to generate monophyletic clades [see Additional file 11]. High bootstrap support (Figure 2a) justify these classifications, with all subfamilies exhibiting bootstrap scores of 100%.

The topology of the phylogenetic tree shown in Figure 2a is supported by three additional lines of evidence. First, we calculated all possible pairwise identities both within and between different groups of OR sequences. With the three exceptions noted above for subfamilies OR102, OR115, and OR125, the minimum percent identity within each subfamily is ≥ 62% [see Additional file 11]. Importantly, the maximum inter-subfamily identity is 44% [see Additional file 12] and the maximum interfamily identity is 38% [see Additional file 13]; both of these values are well below the ≥ 62% identity typically observed between members within a given subfamily. Thus, the sorting of ORs by neighbor joining analysis into distinct families and subfamilies is supported by an analysis based on all possible pairwise identities. Second, highly related OR genes are tightly clustered in the zebrafish genome, with the members of a given subfamily residing adjacent to one another, uninterrupted by more distantly related genes [18,33]. In the present analysis, we found that the assignment of OR subfamilies by neighbor joining analysis indeed is consistent with this genomic organization; out of 23 multigene subfamilies, members from only five (OR111, OR113, OR126, OR128, and OR133) are found in genomic clusters interrupted by genes from other subfamilies (Figure 1) [see Additional file 10]. Third, we constructed a phylogenetic tree using a maximum likelihood algorithm; at both the family and subfamily levels, maximum likelihood analysis yields tree topologies comparable to those derived by neighbor joining [see Additional file 5].

### Comparison of fish and mammalian OR repertoires

To gain additional insight into how the *OR *gene superfamily evolved in vertebrates, the zebrafish ORs were aligned to additional sets of vertebrate OR sequences. *OR *genes were predicted from the genome sequences of two pufferfish species, fugu (*Takifugu rubripes*) [27] and tetraodon (*Tetraodon nigroviridis*) [34], using methods identical to those used for finding zebrafish *OR*s. Forty-four (3 with one disruption) and 42 (6 with one disruption) intact genes were found in fugu [27,29] and tetraodon, respectively [see Additional file 3 and Additional file 4]. As the genome sequence data for these two species represent ~95% and ~92% coverage, respectively, we expect that the *OR *genes identified here comprise the majority of each species' OR repertoire. Thus, the OR repertoires of both pufferfish species appear to be only ~one-third the size of the zebrafish repertoire. A summary of the OR genes identified in zebrafish, fugu, and tetraodon genomes is provided in Table 2.

**Table 1:** Average pairwise identities between odorant receptor families. Pairwise comparisons were performed between each member of a family with each member of the family to be compared. The average percent identity was then calculated for all comparisons between each pair of families. Similar results were obtained for consensus sequences of each family generated from Hidden Markov Models (HMMs) of each family. Zebrafish families are labeled A-H according to Figure 2a. The two major groups of mouse genes are denoted as "Class I" and "Class II." The maximum percent identity between families used to guide family classification of phylogenetic clades was 40% identity.

**A**	100									
**B**	31	100								
**C**	28	34	100							
**D**	29	31	29	100						
**E**	28	28	26	26	100					
**F**	29	29	27	28	32	100				
**G**	25	30	28	26	25	26	100			
**H**	18	18	17	18	19	19	18	100		
**Class I**	**32**	32	29	28	27	28	24	17	100	
**Class II**	28	**38**	28	29	28	29	25	18	30	100
	**A**	**B**	**C**	**D**	**E**	**F**	G	**H**	**Class I**	**Class II**

**Table 2:** Summary of identified teleost OR genes. OR sequences identified in the present study are listed in this table.

	**Zebrafish**	**Fugu**	**Tetraodon**
**Intact genes ^a^**	143 (7)	44 (3)	42 (6)
**Partial genes ^b^**	7	9	4
**Pseudogenes ^c^**	10	4	11
**Total**	160	57	57

^a ^A gene is operationally defined as "intact" if it possesses a full-length OR protein coding sequence with no more than one disruption. For each species, the number of genes with a single disruption is listed in parentheses; the remainder contain no disruptions.

^b ^A sequence encoding ≤ 275 contiguous amino acids, missing specific features characteristic of ORs (see the text for details), or missing start or stop codons is classified as a partial gene.

^c ^A sequence is defined as a pseudogene if it is a partial gene with one or more disruption or a full-length gene with 2 or more disruptions.

Nine-hundred-thirty-five mouse OR sequences [8,9] were either downloaded from Genbank (864 genes) or extracted from MGSCv3 using published coordinates (71 genes) [9]. Phylogenetic trees were computed for ORs from zebrafish and mouse (Figure 2b) [see Additional file 6] and zebrafish, fugu and tetraodon (Figure 2c) [see Additional file 7]. The location of the melanocortin receptor branch represents the root of each tree.

Mouse ORs can be classified into two groups, Class I and Class II, each showing on average greater than 40% intra-group sequence identity [8]. Based on their greater similarity to the limited number of fish *OR *genes identified prior to the present study, Class I genes from amphibians and mammals have been referred to as "fish-like" [8,10,24]. However, our analysis of the complete set of zebrafish *OR *genes indicates that this view cannot be generalized to the entire fish OR repertoire. Mammalian Class I and Class II genes can in fact be grouped more closely with only two out of eight ~equidistantly-related zebrafish families; Class I genes show close similarity to only a small subset of zebrafish *OR *genes (*OR112*-*1*, *OR113*-*1*, *OR113*-*2 *and *OR114*-*1*, which together comprise Family A), and one zebrafish gene (*OR101*-*1*, comprising the single member Family B) clusters together with mammalian Class II genes (Figure 2b). We base these conclusions on phylogenetic reconstructions as determined by neighbor joining (Figure 2b) and maximum likelihood [see Additional file 6], as well as on a separate calculation of average pairwise identities of genes between families (Table 1) [see Additional file 13]. In all cases, the alignment of mouse and zebrafish genes was gap-minimized and trimmed to remove N- and C-terminal tails [see Additional file 2]. Overall, mouse Class I exhibits similar average pairwise identity to the zebrafish families (27.3 ± 4.8% identity [mean ± standard deviation]; range: 17 – 32%) as mouse Class II (27.7 ± 5.5%; range: 18 – 38%); the difference in mean values is not significant in a two-tailed t-test (p = 0.89). Calculations comparing consensus sequences representing each family yielded similar results (data not shown).

A comparison of teleost *OR *genes further reveals that six of the eight zebrafish OR families overlap with pufferfish families; families B and G do not appear to be present in pufferfish (Figure 2c) [see Additional file 7]. In our phylogeny comparing zebrafish and pufferfish genes by neighbor joining, we find low bootstrap support for Family E (score = 47), likely reflecting this family's closer proximity to Family F in the multi-species tree as compared to the tree generated with zebrafish *OR *genes alone (however, Family E has high bootstrap support by maximum likelihood analysis [see Additional file 7]). Interestingly, in zebrafish the most divergent family (Family H) shows only 17–19% identity to other families (versus 25–34% interfamily identity amongst the other families; Table 1). The location of the outgroup melanocortin receptor between Family H and the other families supports the conclusion that this family is the result of a very ancient gene duplication event. Based on the degree of divergence from other *OR *gene families, it is possible that the genes comprising Family H may not in fact encode bona fide odorant receptors. However, the predicted zebrafish Family H receptors retain one of the highly conserved OR signature motifs (see below), and one member of this family (*OR137*-*7*) was previously identified as an EST from a zebrafish olfactory epithelium cDNA library [see Additional file 10]. In addition, when zebrafish Family H sequences were used in BLAST searches of both the non-redundant protein sequence database and the mouse genome sequence, previously identified OR sequences were identified as the closest hits (data not shown). Family H also forms a cluster distinct from non-OR GPCRs in a phylogenetic tree comprising mouse and zebrafish ORs together with a set of 199 non-OR Type I (rhodopsin class) mouse GPCRs [see Additional file 8]. Thus, for the present purposes we consider the Family H sequences operationally as *OR *genes. More generally, this phylogenetic reconstruction based on OR and non-OR GPCRs reveals that the ORs as a group are distinct from the other Type I GPCRS.

A similar phylogeny for vertebrate *OR *genes was recently described [29]. This study placed zebrafish, fugu, Xenopus and chicken *OR *genes into groups roughly comparable to those described here in Figure 2, with Family A corresponding to these authors' Group β (which clusters closely with human Class I genes); the single zebrafish gene comprising Family B falling within Group γ /human Class II; Family C corresponding to Group ε; Families D and G corresponding to Group ζ (which is not a monophyletic clade); Families E and F corresponding to Group δ; and Family H corresponding to Group η. Two highly divergent groups (not identified or retained in our search) – termed κ and θ – were also described, although their identities as *OR *genes are unclear [29].

### Conserved motifs in predicted OR protein sequences

**Figure 3:**
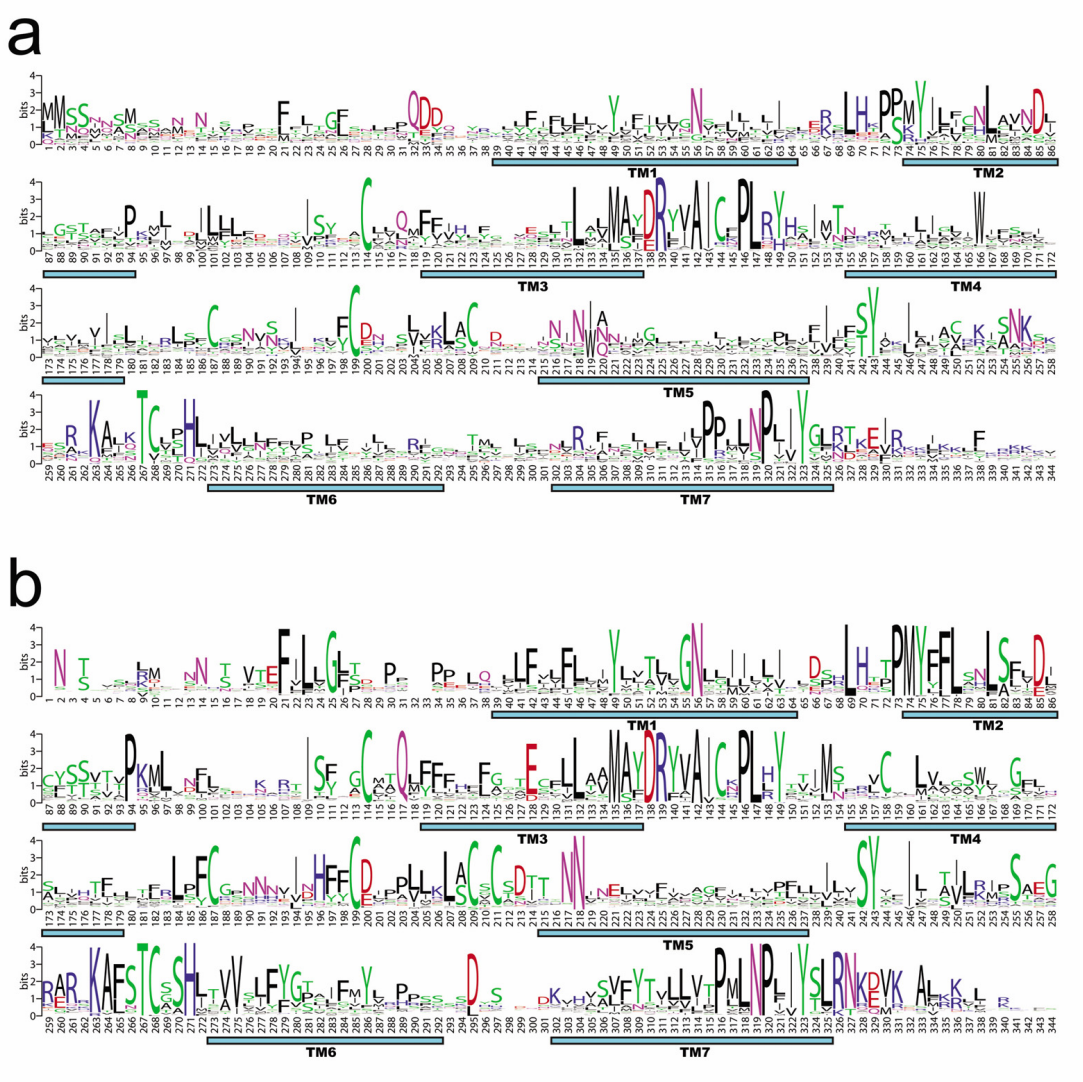
**Sequence logos of zebrafish and mouse OR families**. Conservation of predicted amino acid sequence for the zebrafish **(a) **and mouse **(b) **OR repertoires is shown graphically (see the text). Y axis, information content. X axis, residue position. For this analysis, positions with gaps in more than 95% of sequences, as well as poorly aligned N- and C-terminal sequences, were removed. Positions in the species-specific logos are identical according to this alignment. The logo was generated from this alignment using the program WebLogo (G.E. Crooks, G. Hon, J.-M. Chandonia and S.E. Brenner, personal communication), available at .

Previous studies of vertebrate ORs have identified a number of conserved sequence motifs characteristic of these receptors [8,12,35]. These include the following: an N-linked glycosylation site NX [TS]X in the N-terminal domain; the motif MA [FY] [DE]RYVAIC located at the third transmembrane domain (TM3)/second intracellular loop (IC2) junction which is thought to interact with G-proteins (specifically G_olf_); three conserved cysteine residues in the second extracellular loop (EC2) thought to partake in disulfide bonding; and the motif KAFSTCXSH in IC3 containing an intracellular cysteine conserved in GPCRs and potential phosphorylation sites. We found that these motifs are conserved in all the zebrafish *OR *families, with the exception of Family H, in which only the MAYDRYVAIC motif is conserved. This sequence conservation is illustrated by a sequence logo generated from the alignment of predicted full-length zebrafish OR coding sequences (Figure 3a). In this representation, the relative frequency with which an amino acid appears at a given position is reflected by the height of its one-letter amino acid code in the logo, with the total height at a given position proportional to the level of sequence conservation. Interestingly, when compared to the sequence logo representing the alignment of mouse Class I and Class II ORs (Figure 3b), the zebrafish OR logo shows lower conservation amongst the predicted zebrafish receptor sequences (reflected by the more numerous and shorter letters at individual positions in the logo), revealing the greater diversity within the zebrafish vs. mouse OR superfamily (Table 1).

### Adaptive evolution of OR genes

**Figure 4:**
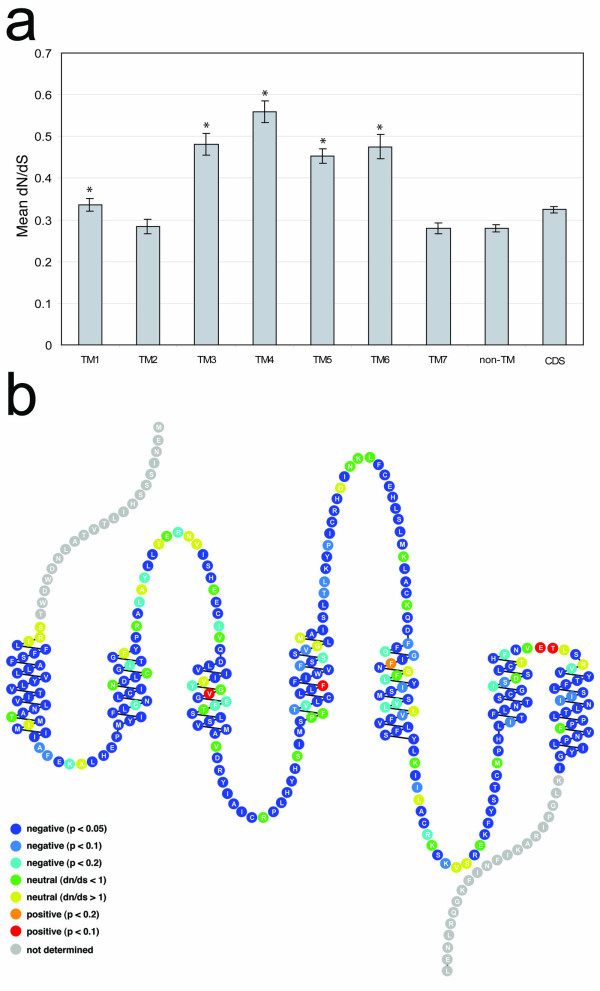
**Sites under positive and negative selection in OR coding sequences**. Nucleotide alignments were generated from the corresponding amino acid alignment [see Additional file 2] after removal of N- and C-terminal sequences and gap removal with respect to OR124-3, and subjected to two analyses of selective pressure. **(a) **Analysis of dN/dS ratios by sub-regions of OR coding sequences indicates that OR genes in general are under negative or purifying selection (dN/dS < 1). However, analysis of informative pairwise comparisons reveals that transmembrane domains (TMs) 3–6, and to a lesser degree TM1, have significantly higher average pairwise dN/dS ratios when compared with the average for non-transmembrane coding sequence (asterisks; p < 1 × 10^-3^). Error bars show standard errors of the means. **(b) **A schematic representation of OR124-3 (an example OR) with transmembrane domains one through seven shown from left to right. SLAC analysis reveals sites under positive selection (dN/dS > 1) with p < 0.1 (red), p < 0.2 (orange), p < 0.5 (yellow), and sites under negative selection (dN/dS < 1) with p < 0.05 (dark blue), p < 0.1 (light blue), p < 0.2 (turquoise), p < 0.5 (green). The null hypothesis is that a site is neutrally evolving with dN/dS = 1. Yellow and green residues may be considered neutrally evolving. Sites on the representative OR124-3 sequence with dN/dS > 1 and p < 0.1 are: V108 (dN/dS = 1.39), F145 (dN/dS = 1.52), E261 (dN/dS = 1.48) and T262 (dN/dS = 1.42). Snake plot generated using the RbDe web service [48].

What evolutionary processes might explain the diversity of *OR *gene sequences? This diversity could be the result of genetic drift, with polymorphisms in the population being fixed at a rate consistent with the absence of selective pressure. Alternatively, the sequence diversity could reflect true functional divergence, perhaps in the ligand specificities of the encoded receptors. Considering the diversity of OR proteins encoded in vertebrate genomes and the even greater diversity of compounds detected by these receptors, ligand binding sites within ORs may be expected to be under positive selective pressure as organisms evolve new receptor proteins to recognize odorant compounds. As for other Type 1 (rhodopsin class) GPCR ligands, odorants are thought to bind to OR proteins in the plane of the membrane, in contact with residues in the transmembrane domains [2]. Consistent with this notion, previous studies have demonstrated that odorant receptor genes have been subject to positive selective pressure, especially in the transmembrane domains thought to coordinate odorant binding [13,36] (however, see [37]). We therefore attempted to pinpoint the precise codon sites – and thus the amino acid residues – that may have been subjected to positive selection during the evolution of the zebrafish OR superfamily. To this end, we used the relative frequency of non-synonymous vs. synonymous codon substitutions to assess the selective processes acting on these receptor genes [38]. Where there is no positive or negative selection on a sequence, the number of non-synonymous changes relative to the number of possible non-synonymous changes (dN) would be equal to the number of synonymous changes relative to the number of possible synonymous changes (dS) – i.e., dN/dS = 1. Significant deviations of dN/dS from unity reflect selection on the sequence; a dN/dS ratio > 1 indicates that a region has undergone positive selection, whereas a dN/dS ratio < 1 indicates negative or "purifying" selection [38]. For our analysis, we aligned 136 full-length zebrafish OR coding sequences containing no disruptions and calculated dN/dS ratios based on a gap-minimized alignment (Table 3 and Figure 4a). We found that when the OR coding sequence is partitioned broadly into transmembrane domains (TMs) 1–7 and non-transmembrane domains (excluding the N- and C-terminal tails), none of these regions exhibits positive selection. Rather, with average dN/dS ratios <1, these protein regions all appear to be under negative or purifying selection. Interestingly, TMs 1, 3, 4, 5, and 6 display significantly higher average dN/dS ratios than the combined intracellular and extracellular loops (p < 1 × 10^3^; see Figure 4a). The observation that these transmembrane domains were in general under less purifying selection than other regions of the protein is consistent with the possibility that they may have adapted to bind different odorants. In contrast, TM2 and TM7 display significantly lower average dN/dS ratios compared to the complete coding sequence (p < 0.05). The apparently stronger negative selection on TM2 and TM7 (as compared to the other transmembrane regions) suggests that these transmembrane domains subserve a common – perhaps structural – role in these receptors.

**Table 3:** Comparison of selective pressure by transmembrane domain.

	**TM1**	**TM2**	**TM3**	**TM4**	**TM5**	**TM6**	**TM7**	**non-TM**	**CDS**
**Length (amino acids)**	26	21	19	24	25	19	24	110	268
**Mean pairwise dN/dS^a^**	0.330	0.276	0.457	0.574	0.437	0.513	0.282	0.304	0.325
**TM vs loops p-value (2 -tailed t test)^b^**	9.9E-04	0.81	2.1E-12	7.6E-21	1.2E-18	2.5E-10	1.00		
**TM vs CDS p-value (2 -tailed t test)^b^**	0.45	0.025	2.1E-08	5.9E-16	7.9E-12	6.0E-07	0.0024		
**Number of positively selected sites (p < 0.1)^c^**	0	0	1	1	0	0	0	2	4
**Number of negatively selected sites (p < 0.1)^c^**	21	16	12	15	15	14	19	80	192

^a ^The mean ratios of non-synonymous substitutions per non-synonymous site (dN) to synonymous substitutions per synonymous site (dS) were calculated for each transmembrane region, the aggregate of the non-transmembrane regions, as well as the entire *OR *coding sequence. dN/dS ratios were calculated only for pairwise comparisons in which mutational saturation had not been reached. A constant set of 262 informative pairs was used for all calculations.

^b ^Probabilities that the average dN/dS ratio for the particular TM domain is no different than the average for the entire CDS or the non-TM domains, using a two-tailed t test.

^c ^Number of sites with probabilities greater than 0.9 (p < 0.1) of being positively or negatively selected for each domain. Ancestral sequences were reconstructed, and at every non-constant site, dN and dS were calculated. If dN < (or >) dS, a p-value derived from a two-tailed binomial distribution was used to assess significance.

We hypothesized that specific codon sites corresponding to odorant-binding residues might have been positively selected as coding sequences diverged after gene duplication events. Accordingly, identification of these sites would suggest possible ligand binding sites. We therefore performed a site-by-site analysis of dN/dS ratios based on the alignment of the set of 136 full-length intact coding sequences used above. To avoid a potentially high rate of false positives common with pooled site methods (see [39]), we used the more conservative likelihood individual site (IS) method [40] based on the original proposed IS approach [41]. The phylogenetic relationships between sequences were determined and a substitution model was estimated from the data. The ancestral codon sequences at each node were then reconstructed and the dN and dS values were calculated for each codon site. In Figure 4b, the probability of being under positive or negative selection (dN/dS values different than dN/dS = 1.0) for each codon site is indicated on a snake plot of a representative OR amino acid sequence, OR124-3 (see also Table 3). By these criteria, only two sites within the transmembrane domains (one in TM3 and one in TM4) appear to have been subjected to positive selection, consistent with the notion that they may play a role in contacting ligands. Interestingly, two adjacent sites in the short third extracellular loop (very close to the top of TM6) also exhibit dN/dS ratios > 1. Overall, our characterization of dN/dS ratios reveals a striking paucity of sites exhibiting signs of positive selection, possibly reflecting the dominating influence of negative selection throughout the receptor coding region. Alternatively, since non-synonymous substitutions may occur only sporadically over evolutionary time, the signatures of less recent substitutions may no longer be detected by this analysis of the entire zebrafish OR family.

### Evolution of the vertebrate OR gene repertoire

The characterization of the complete *OR *repertoires from both fish and mammalian species allows an informed analysis of *OR *gene evolution in the vertebrate lineage. One noteworthy feature of our phylogenetic reconstruction is the presence of a group of zebrafish and pufferfish subfamilies which together form a putative OR family (Family H) more divergent than the other families are to each other (Figure 2c and Table 1); based on a BLAST search of the mouse genome using representative zebrafish Family H sequences, this family is absent from the mouse. We hypothesize that the node between this branch of the tree and the others is the root representing the most ancient gene duplication event observable in the teleost lineage. This is supported by the placement at this node of the melanocortin receptor (outgroup) branch. In addition, when we aligned five OR sequences from lamprey [25,42] to the teleost ORs, they formed two additional families on either side of the melanocortin receptor branch, one which is clearly an OR family (more similar to teleost OR families A-G than to H) and one which appears as an outgroup (equidistant from all teleost families A-H and more dubious as an OR family) [see Additional file 9]. Since the lamprey diverged before the teleost/tetrapodon split, these observations provide further support for this node as the root of the tree. It should be noted, however, that until the lamprey genome has been fully sequenced, we will only have a partial picture of the ancestral OR repertoire. We expect that characterization of the entire lamprey OR repertoire will shed light on more ancient evolutionary events.

From our analysis of mammalian, teleost and lamprey OR sequences, we propose the following model for OR gene evolution in vertebrates. *OR *genes in present-day vertebrates likely descended from eight ancestral *OR *genes (or gene families) that existed at the time of the split between ray-finned and lobe-finned fish (the ancestors of teleosts and tetrapods, respectively) approximately 450 million years ago (mya) [43]. A phylogenetic reconstruction based on mouse and zebrafish ORs and 199 mouse non-OR GPCRs [see Additional file 8] indicates that the ORs form a group distinct from all other Type I GPCRs, possibly reflecting a very ancient duplication event(s) and/or rapid divergence of the ORs in the evolution of Type I GPCRs. Our estimate of ancestral *OR *gene number is based on the identification of 8 *OR *gene families in teleosts, two of which show somewhat higher similarity to the 2 *OR *gene families in mammals. The grouping of zebrafish and pufferfish *OR *genes into common families indicates that the gene duplication events that gave rise to the major OR families probably occurred prior to the speciation of teleosts. In addition, the greater similarities between zebrafish Family A and mouse Class I, and between zebrafish Family B and mouse Class II infer that the ancestral genes for these families existed before the tetrapodon/teleost split. Our model therefore suggests a history during which ancestral genes or gene families were selectively lost during the evolution of the different vertebrate lineages. Of the ancestral families, zebrafish retained 8 families, fugu and tetraodon retained 6 families, and mammals retained 2 families. It should be noted that the low bootstrap score (47) for Family E in the comparison of zebrafish and pufferfish *OR *genes (Figure 2c) raises the possibility that Families E and F (which are adjacent to each other in the teleost phylogenetic tree) may have arisen from a more recent duplication in the teleost lineage. Alternatively, the genes in these groups may have been subjected to gene conversion events, with the effect of homogenizing the sequences between these two families.

It is also possible that the 4–6 gene families unique to teleosts descended from Family A/Class I and/or Family B/Class II ancestral genes, after the tetrapodon/teleost split. Such a scenario seems unlikely, however, considering the roughly equivalent degree of divergence exhibited between 7 out of the 8 teleost gene families (including Families A and B). Moreover, amphibian and avian OR genes can be grouped into 6 out of the 8 identified OR families (Families A, B/Class II, C, E, F and H), further implicating the presence of common ancestral genes for these families prior to the tetrapodon/teleost split [29].

Mechanisms of gene or family loss in a particular vertebrate lineage may have involved a number of processes, for example, gene conversion, pseudogenization of all genes in a family, unequal crossover recombination events during meiosis, or larger chromosomal rearrangements. From the available data we cannot infer the precise order and rate of *OR *gene family expansion and contraction, or speciation events. Nonetheless, six of the retained *OR *gene families were subject to a substantial net expansion and diversification in zebrafish (and to a lesser degree in the pufferfish), while the other two ancestors gave rise to the present-day mammalian Class I and Class II ORs as well as a small number of zebrafish genes. We hypothesize that relaxed selective pressure on a subset of the ancestral tetrapodon OR repertoire led to the loss of major *OR *gene families in the mammalian lineage. The expansion within the two remaining gene families was likely driven by the adaptation to the terrestrial odorous environment. Thus, different selective pressures found in the aquatic and terrestrial environments led to different sizes and shapes of the OR repertoires of fish and mammals.

It is generally thought that the diversity of OR sequences – as represented in the number of receptor families – underlies the diversity of chemical structures or "odor space" that can be detected by an organism's olfactory system. Thus, with ~6–8 *OR *gene families retained over evolutionary time (vs. 2 in mammals), fish may be capable of detecting a larger diversity of chemical structures than mammals. However, the larger total number of OR sequences in mammals (~1,000 vs. ~100 in fish) presumably allows a finer discrimination amongst the compounds that are detected by the mammalian olfactory system.

## Methods

### Iterative data mining

Genome-wide searches of the third (Zv3) and fourth (Zv4) draft zebrafish genome assemblies  made available by the Sanger Center on Nov 27, 2003, and July 12, 2004, respectively, were performed several times using the predicted ORs from each previous round to increase our querying power. This iterative data-mining approach has been published for finding OR genes in the mouse genome [8]. A detailed description of our protocol is provided in the Supplement [see Additional file 1].

### Alignment and tree construction

For multiple alignments of OR genes, ClustalX 1.81 [44] was used with default parameters and gaps were inspected manually and edited in xced  to ensure integrity of transmembrane domains and proper alignment of anchoring OR motifs. N- and C-terminal tails were trimmed for all alignments. The neighbor-joining algorithm as implemented by PFAAT  was used to generate unrooted phylogenetic trees from these alignments using the BLOSUM 50 similarity matrix; positions with greater than 40% gaps were excluded. One thousand bootstraps were performed to assess the support at each tree node. Trees were visualized with *unrooted *[45]. Maximum likelihood analysis was carried out using PHYML [46] on the same processed amino acid alignments described above. Bootstrap analysis with 100 replicates was carried out using the JTT model of amino acid substitution. The consensus tree including bootstrap support for each node was plotted for each dataset using either ATV [47] or *unrooted*.

The sequences used for comparison to the zebrafish *OR *genes were obtained from Genbank and included the set of intact MORs [Genbank: AY072961] – [Genbank: AY074256] [8] plus 71 newly identified *OR *genes extracted from MGSCv3 using coordinates from the online supplement to [9], 5 full-length lamprey *OR *receptors [Genbank: AAC82383, Genbank: AAC82384, Genbank: AAC82385, Genbank: CAA10135, Genbank: CAA10136]. [25], the zebrafish melanocortin 1, 2, 3, 4, 5a and 5b receptors [Genbank: NP_851301.1, Genbank: NP_851302.1, Genbank: NP_851303.1, Genbank: NP_775385.1, Genbank: NP_775386.1, Genbank: NP_775387.1], and 199 mouse non-OR Class A GPCRs extracted from the GPCRDB . Fugu and tetraodon *OR *sequences were predicted from the current genome assemblies [27,34] using the methods described above for zebrafish.

For the calculation of percent identities, mouse and zebrafish amino acid sequences were multiply aligned and trimmed of their N- and C-terminal tails as described above. Calculations of average, minimum and maximum intra-family, inter-family, intra-subfamily and inter-subfamily percent identities were based on percent identities calculated for all pairs of amino acid sequences in this multiple alignment.

### dN/dS analysis

The dN/dS ratios for multi-codon regions (i.e. individual transmembrane domains or loop regions) of the odorant receptor coding sequence were determined using previously published methods [38]. To make inferences about selective pressure (positive and negative selection) on individual codons (sites) within the coding sequence of the zebrafish OR genes, the Single Likelihood Ancestor Counting (SLAC) package , which implements the Suzuki-Gojobori method [41], was used. Details regarding both of these methods are provided in the Supplement [see Additional file 1].

### Genbank accession numbers

All sequences described in this study have been deposited in Genbank under accession numbers [Genbank: DQ305986] – [Genbank: DQ306145] (zebrafish), [Genbank:DQ306146] – [Genbank: DQ306202] (tetraodon), and [Genbank:DQ306203] – [Genbank: DQ306259] (fugu).

## Authors' contributions

TSA carried out the analysis. Both authors participated in the design of the study and writing of the manuscript.

## Supplementary Material

Additional File 1 Supplement: Methods and legends for Figures S1-S8 and Tables S1-S4 Click here for file

Additional File 2 Figure S1. Multiple sequence alignment of zebrafish OR amino acid translations. Click here for file

Additional File 3 Figure S2. Multiple sequence alignment of fugu OR amino acid translations. Click here for file

Additional File 4 Figure S3. Multiple sequence alignment of tetraodon OR amino acid translations. Click here for file

Additional File 5 Figure S4. Phylogeny of zebrafish ORs using maximum likelihood analysis. Click here for file

Additional File 6 Figure S5. Phylogeny of zebrafish and mouse ORs using maximum likelihood analysis. Click here for file

Additional File 7 Figure S6. Phylogeny of zebrafish, fugu and tetraodon ORs using maximum likelihood analysis. Click here for file

Additional File 8 Figure S7. Phylogeny of zebrafish and mouse ORs rooted by mouse non-OR GPCRs. Click here for file

Additional File 9 Figure S8. Phylogeny of zebrafish, fugu, tetraodon and lamprey ORs. Click here for file

Additional File 10 Table S1. The zebrafish OR repertoire. Click here for file

Additional File 11 Table S2. Pairwise intra-subfamily percent identities for zebrafish OR subfamilies. Click here for file

Additional File 12 Table S3. Pairwise inter-subfamily percent identities for zebrafish OR subfamilies. Click here for file

Additional File 13 Table S4. Pairwise inter-group percent identities for zebrafish OR families and mouse Class I and Class II ORs. Click here for file
