# Supplementary material for: The odorant receptor repertoire of teleost fish
Source: BMC Genomics. 2005 Dec 6;6:173. doi: 10.1186/1471-2164-6-173 (PMC1325023; doi:10.1186/1471-2164-6-173)
Supplement: Additional File 1 — Supplement: Methods and legends for Figures S1-S8 and Tables S1-S4 [file 1471-2164-6-173-S1.pdf]

Supplement: Methods and legends for Figures S1-S8 and Tables S1-S4

Methods

Iterative data mining

Genome-wide searches of the third release of the third (Zv3) and fourth (Zv4) draft zebrafish genome assemblies (ftp://ftp.ensembl.org/pub/assembly/zebrafish/) were performed several times, using the predicted ORs from each previous round to increase our querying power. This iterative data-mining approach [1] was implemented as follows:

1. Profile Hidden Markov Model construction. For the first iteration, a total of 25 full-length receptor sequences corresponding to OR subfamilies 2, 4, 5, 7, 9, and 13 [2] were aligned using ClustalW, and the alignment was used to build a profile Hidden Markov Model (HMM) using the HMMER software package (http://hmmer.wustl.edu/). In subsequent iterations, new OR sequences were incorporated into a representative set of deduced OR peptide sequences each with <50% identity to any other zebrafish OR sequence. For the second iteration, this consisted of 32 sequences out of 115 complete non-pseudogenic sequences. These were aligned with ClustalW with default parameters and a new profile HMM was constructed. These profiles were used in the subsequent gene prediction step by the program Genewise.

2. BLAST search. Representative zebrafish OR sequences (with <40% pairwise protein identity) were used as query sequences for a TBLASTN search in the ZFISH3 assembly. All hits with an E-value < 5e-5 and at least one high-scoring pair (HSP) longer than 150 amino acids in length were extracted from the database. A non-redundant set of contig sequences was assembled from these results.

3. Gene prediction. For each contig, the contig sequence was used as a query in a BLASTX search of a combined database of all known OR protein sequences in Genbank and all deduced zebrafish OR protein sequences from the previous iteration. Regions of BLAST similarity were defined by taking the union of overlapping HSPs or close neighboring HSPs (the distance between them less than 1.5 kb). These regions were extended in the 5′ and 3′ directions an additional 750 bp to define the region in which to perform detailed prediction of OR coding sequences. Genewise was run with the profile constructed in step 1 on each of these regions of the contig. Both strands were searched and pseudogenes were allowed. Predicted exons less than 700 bp in length were set aside for subsequent classification. For each exon, the end of the exon was extended to the next stop codon. An attempt was made to find an appropriate start codon within ten codon positions first in the 5′ direction and then in the 3′ direction. If no start codon was found in this window, the search was extended 5′ until the first ATG was found before reaching an in-frame stop codon. If no start codon was found, the gene was recorded as a partial coding sequence and the original start coordinate defined by Genewise was retained.

4. Conceptual translation. All CDS sequences were subjected to conceptual translation by FASTY search and alignment to the combined database described in step 3. (ftp://ftp.virginia.edu/pub/fasta/) [3, 4]. The portion of the conceptually translated sequence aligned to the top FASTY hit was then extended using standard translation to the ends of the CDS sequence. The positions of premature stop codons and frame shifts were noted and, in cases where two or more disruptions were found, the gene was marked as a pseudogene. Protein sequences less than 275 amino acids in length were annotated either as partial genes (if they had no disruptions) or as pseudogenes (if they had one or more disruptions). Each protein sequence was used as a query in a BLASTP

search of the non-redundant protein database and the top hit and percent identity of the best HSP were recorded.

5. Manual inspection. Considering the incomplete nature of the genome assembly, partial genes were not necessarily considered pseudogenes. Future improvements in the quality of the sequences, gap-filling and revisions in the assembly may eventually result in the determination of complete coding sequences for these genes. In some cases, conceptual translation resulted in two closely spaced frame shifts, while the original sequence had no disruptions in the open reading frame. These were re-translated and re-annotated as genes, not pseudogenes. Some *OR*s on short scaffolds were removed as duplicates if they shared more than 97% identity with another *OR* found on a longer mapped scaffold and the flanking DNA regions were almost identical as compared in the Artemis Comparison Tool (Sanger Institute) (http://www.sanger.ac.uk/Software/ACT/). In addition, three entire scaffolds were removed because they were duplicated by finished genomic sequence. Most of the manual inspections were done using the Artemis annotation tool developed at the Sanger Institute (http://www.sanger.ac.uk/Software/Artemis/). All *OR* annotations were maintained in Genbank flat file format of the contig sequences themselves. In nearly all cases, the top BLAST hit in the non-redundant protein database was an odorant receptor, validating the specificity and conservative nature of our approach. However, a few predicted genes matched non-odorant receptors and were removed from the set. If additional open reading frames with lengths of approximately 1000 base pairs were detected adjacent to already annotated *OR* genes, those exhibiting *OR* motifs and sequence similarity to known odorant receptors were added to the gene set for the next iteration.

6. Database update. All annotated non-duplicate contig Genbank sequence files were processed using custom PERL scripts to build FASTA databases of conceptual translations, standard translations, and CDS sequences.

dN/dS analysis

The dN/dS ratios for multi-codon regions (i.e. individual transmembrane domains or loop regions) of the odorant receptor coding sequence were determined with SNAP (http://www.hiv.lanl.gov/content/hiv-db/HTML/snap.html) using previously published methods [5]. The set of 136 full-length non-disrupted zebrafish *OR* coding sequences was codon-aligned and subalignments were made which corresponded to each transmembrane domain (as determined by comparison to previously published OR transmembrane domain sequences and alignment with bovine rhodopsin), combined non-transmembrane domains (intra- and extra-cellular loops), and the N- and C-termini-trimmed sequence . For each pair of sequences the following values were calculated: the observed number of synonymous (Sd) and nonsynonymous (Sn) substitutions, the number of potential synonymous (S) and nonsynonymous (N) substitutions, the proportions of Sd/S (ps) and Sn/N (pn), and the corresponding Jukes-Cantor corrected proportions dN and dS. In many pairwise comparisons, mutational saturation had been reached (ps or pn > 0.75) and therefore these comparisons were subsequently ignored. To avoid a potential issue with non-independence of points when using potentially different sets of gene pairs to compare multiple OR domains, a common set of gene pairs informative for all of the considered domains was selected for further analysis. The average of the dN/dS ratios for this set was calculated for each domain.

To make inferences about selective pressure (positive and negative selection) on individual codons (sites) within the coding sequence of the zebrafish OR genes, the Single Likelihood Ancestor Counting (SLAC) package (http://www.datamonkey.org), which implements the Suzuki-Gojobori method [6], was used. Briefly, the method by which this was done is as follows. First, a best-fitting nucleotide substitution model was automatically selected by fitting several such substitution models to both the data and a neighbor-joining tree generated from the alignment described above. Holding the substitution rates and branch lengths obtained at this step constant, a codon model was fit to the data and a global dN/dS ratio calculated. The ancestral sequences at each codon were reconstructed using maximum likelihood. The expected (normalized) and observed numbers of synonymous and non-synonymous substitutions (ES, EN, NS, NN) for every non-constant site were then determined. dN = NN/EN and dS = NS/ES were computed, and if dN < dS (negative selection) or dN > dS (positive selections), a p-value derived from a two-tailed extended binomial distribution was used to assess significance. Tests on simulated data (S.L.K. Pond and S.D.W. Frost, methods available at http://www.datamonkey.org) show that p values less than or equal to 0.1 identify nearly all true positives with a false positive rate generally below the nominal p value; for actual data, the number of true positives at a given false positive rate is lower. Therefore, in the present study, several thresholds for significance were considered with respect to identification of potential odorant-coordinating residues.

References

1. X Zhang, S Firestein: **The olfactory receptor gene superfamily of the mouse**. *Nat. Neurosci.* 2002, **5**:124-133.

2. JC Dugas, J Ngai: **Analysis and characterization of an odorant receptor gene cluster in the zebrafish genome**. *Genomics* 2001, **71**:53-65.

3. WR Pearson: **Flexible sequence similarity searching with the FASTA3 program package**. *Methods Mol Biol* 2000, **132**:185-219.

4. WR Pearson, T Wood, Z Zhang, W Miller: **Comparison of DNA sequences with protein sequences**. *Genomics* 1997, **46**:24-36.

5. M Nei, T Gojobori: **Simple methods for estimating the numbers of synonymous and nonsynonymous nucleotide substitutions**. *Mol Biol Evol* 1986, **3**:418-26.

6. Y Suzuki, T Gojobori: **A method for detecting positive selection at single amino acid sites**. *Mol Biol Evol* 1999, **16**:1315-28.

7. X Zhang, I Rodriguez, P Mombaerts, S Firestein: **Odorant and vomeronasal receptor genes in two mouse genome assemblies**. *Genomics* 2004, **83**:802-11.

8. J Freitag, A Beck, G Ludwig, L von Buchholtz, H Breer: **On the origin of the olfactory receptor family: receptor genes of the jawless fish (Lampetra fluviatilis)**. *Gene* 1999, **226**:165-174.

9. A Berghard, L Dryer: **A novel family of ancient vertebrate odorant receptors**. *J Neurobiol* 1998, **37**:383-92.

10. AL Barth, NJ Justice, J Ngai: **Asynchronous onset of odorant receptor expression in the developing zebrafish olfactory system**. *Neuron* 1996, **16**:23-34.

11. CA Byrd, JT Jones, JM Quattro, ME Rogers, PC Brunjes, RG Vogt: **Ontogeny of odorant receptor gene expression in zebrafish, Danio rerio**. *J Neurobiol* 1996, **29**:445-58.

12. F Weth, W Nadler, S Korsching: **Nested expression domains for odorant receptors in zebrafish olfactory epithelium**. *Proc Natl Acad Sci U S A* 1996, **93**:13321-6.

13. AL Barth, JC Dugas, J Ngai: **Noncoordinate expression of odorant receptor genes tightly linked in the zebrafish genome**. *Neuron* 1997, **19**:359-69.

14. Y Niimura, M Nei: **Evolutionary dynamics of olfactory receptor genes in fishes and tetrapods**. *Proc Natl Acad Sci U S A* 2005, **102**:6039-44.

Figure and table legends

Figure S1. Multiple sequence alignment of zebrafish OR amino acid translations. The deduced amino acid sequences of the 140 full-length genes and 4 full-length pseudogenes predicted from the zebrafish genome assemblies were aligned with *mafft* (1000 iterations) and refined using ClustalX1.81. Predicted transmembrane domains are labeled. Locations of N- and C-terminal trimming are marked with downward-pointing arrows. Pseudogenes are indicated with the suffix "P." Identical residues are shaded dark gray and similar residues shaded light gray. The threshold for consensus shading is 50%.

Figure S2. Multiple sequence alignment of fugu OR amino acid translations. The deduced amino acid sequences of the 53 genes and 4 pseudogenes predicted from the fugu genome assembly were aligned with default parameters using ClustalX1.81 and gaps were manually edited with BioEdit. Predicted transmembrane domains are labeled. Sequences are identified by assembly scaffold and a sequential numeric suffix. Locations of N- and C-terminal trimming are marked with downward-pointing arrows. Pseudogenes are indicated with the suffix "P." Identical residues are shaded dark gray and similar residues shaded light gray. The threshold for consensus shading is 50%.

Figure S3. Multiple sequence alignment of tetraodon OR amino acid translations. The deduced amino acid sequences of the 47 genes and 10 pseudogenes predicted from the tetraodon genome assembly were aligned with default parameters using ClustalX1.81 and gaps were manually edited with BioEdit. Predicted transmembrane domains are labeled. Sequences are identified by assembly scaffold and a sequential numeric suffix. Locations of N- and C-terminal trimming are marked with downward-pointing arrows.

Pseudogenes are indicated with the suffix "P." Identical residues are shaded dark gray and similar residues shaded light gray. The threshold for consensus shading is 50%.

Figure S4. Phylogeny of zebrafish ORs using maximum likelihood analysis. A maximum likelihood tree was constructed based on an alignment of the predicted amino acid sequences of the 136 non-disrupted full-length *OR* genes identified from the zebrafish genome. One hundred bootstrap replicates were performed and the consensus tree is shown. Bootstrap values are shown at each node in red and those corresponding to family-level nodes are indicated with stars. *OR* genes are named by subfamily and branches are colored by family (colors correspond to the OR families shown in Figure 2a of the main text). The eight gene families are labeled A-H. The melanocortin receptor branch (dotted lines) indicates the root of the tree.

Figure S5. Phylogeny of zebrafish and mouse ORs using maximum likelihood analysis. A maximum likelihood tree was constructed based on an alignment of the following sets of genes: the mouse odorant receptors, mORs [8, 9]; the 136 intact zebrafish ORs with no disruptions identified in this study (highlighted in red); and mouse melanocortin receptors (mcr). One hundred bootstrap replicates were performed and the consensus tree is shown. Bootstrap values are shown at selected nodes. The eight zebrafish OR gene families are labeled A-H, and the two mouse families are labeled Class I and Class II. The melanocortin receptor branch (dotted line) indicates the root of the tree.

Figure S6. Phylogeny of zebrafish, fugu and tetraodon ORs using maximum likelihood analysis. A maximum likelihood tree was constructed based on an alignment of the predicted amino acid sequences of the 136 non-disrupted full-length

OR genes from zebrafish, 42 non-disrupted genes from fugu, and 42 non-disrupted genes from tetraodon. One hundred bootstrap replicates were performed and the consensus tree is shown. Bootstrap values are shown at each node in red and those corresponding to family-level nodes are indicated with stars. The 8 identified OR families and are labeled A-H as in Figure 2 of the main text and Figure S4. Zebrafish genes are colored are colored as in Figure S4. Fugu genes are shown in black and tetraodon genes in dark blue. The melanocortin receptor branch (dotted lines) indicates the root of the tree. In accord with neighbor joining analysis (Figure 2c of the main text), the fugu and tetraodon *OR* genes sort to all but two of the identified OR families (Families B and G).

Figure S7. Phylogeny of zebrafish and mouse ORs rooted by mouse non-OR GPCRs. The following sets of genes were aligned: the mouse odorant receptors, mORs [1, 7]; the subset of 136 intact zebrafish ORs with no disruptions identified in this study; and 199 mouse non-odorant rhodopsin-like GPCRs downloaded from the G protein-Coupled Receptor Database (http://www.gpcr.org/7tm/). A phylogenetic tree was then generated by neighbor joining. The expanded representation of non-OR GPCRs in this phylogeny more clearly demonstrates the segregation of OR and non-OR GPCRs as well as the ~equal distance of zebrafish families C-H from the mouse Class I and Class II OR genes.

Figure S8. Phylogeny of zebrafish, fugu, tetraodon and lamprey ORs. The following sets of genes were aligned with ClustalW: the intact zebrafish, fugu, and tetraodon ORs identified in this study; five lamprey ORs [8, 9]; and the zebrafish melanocortin receptors 1-5 (mcr). A phylogenetic tree was then generated by neighbor joining. The mcr branch (dotted line) indicates the root of the tree.

Table S1. The zebrafish OR repertoire. Subfamilies were determined by examination of a PHYLIP distance matrix generated by ClustalX and the phylogenetic tree generated from the alignment of all full-length intact zebrafish ORs. Subfamilies are monophyletic with 100% bootstrap support and exhibit greater than 60% intra sub-family identity. *OR* genes were renamed according to subfamily starting at subfamily 101 to avoid confusion with previous naming systems. The prefix "OR" stands for "Odorant Receptor." Within subfamilies, genes were numbered sequentially according to genomic position. Putative pseudogenes are indicated with the suffix "P." Also provided in this table are the Genbank accession numbers, identity with previously published OR sequences [2, 10-14] and OR-encoding ESTs, classification as gene or pseudogene, classification as full-length or partial sequence, the number of coding sequence disruptions, and coordinates in the annotated sequence as well as the Zv4 assembly coordinates and chromosomal coordinates.

Table S2. Pairwise intra-subfamily percent identities for zebrafish OR subfamilies. Pairwise comparisons were performed between each member of a subfamily. The average percent identity was then calculated for all comparisons within a subfamily. Average, minimum and maximum percent identities exhibited among members of each subfamily are listed.

Table S3. Pairwise inter-subfamily percent identities for zebrafish OR subfamilies. Pairwise comparisons were performed between each sequence and all other sequences not included in the querying gene's subfamily. Average, minimum and maximum inter-subfamily percent identities are listed.

Table S4. Pairwise inter-group percent identities for zebrafish OR families and mouse Class I and Class II ORs. Pairwise comparisons were performed between members of different groups. Average, minimum and maximum inter-group percent identities are listed.
