# Supplementary material for: The odorant receptor repertoire of teleost fish
Source: BMC Genomics. 2005 Dec 6;6:173. doi: 10.1186/1471-2164-6-173 (PMC1325023; doi:10.1186/1471-2164-6-173)
Supplement: Additional File 2 — Figure S1. Multiple sequence alignment of zebrafish OR amino acid translations. [file 1471-2164-6-173-S2.pdf]

```
                                     ⇓        TM1                           TM2
                       *        20        *        40        *        60        *        80        *
OR106-12 : MANE-------NYTVKNVDSLIITGFDHLQ--NQKLLGFLVLISYMLILLGNGINLFIILTDRHL-HKPMYILICNLAVVDIMYSSTCSTTMISVLIA :  88
OR106-11 : MAN--------ETFVKNVDSFIITGFDHLQ--NQKLLGSLILITYILILLGNGINLFIISTDRHL-HKPMYILICNLAVVDVMYSSTCSTTMISVLLA :  87
OR106-1  : MG---------NYTVNNVDSFIITGFDHLQ--NQKLLGSLILITYILVLLGNGINLCVISTNRHL-HKPMYILICNLAVVDVMYSSTCSTTMISVLLA :  86
OR106-2  : MAAE-------NYSVRNVDSFIITGFDHLQ--NQTLLGSLILITYILILLGNGINLFIILSDRHL-HKPMYILMCNLAVVDIIYTSTCSTTMISVLLA :  88
OR106-3  : MAAE-------NSTMKNIDSFIITGFDHLQ--NQKLLGSLILITYILILLGNGINLCIILADKHL-YKPMYILMCNLAVVDIIYTSTCSTTMISVLLA :  88
OR106-4  : MANE-------TYFVKNVDSFTITGFDHLQ--NQKLLGFLILISYMLILLGNGINLCIISTNRHL-HKPMYILICNLALADIIYTTISSATMISVLLA :  88
OR106-6  : MANA-------TYFVKNVDSFIITGFDHLQ--NQKILGSLILITYILILLGNGINLCVISTNRHL-HKPMYILICNLAVVDIMYTSACSTTMISVLLA :  88
OR106-8  : MANV-------TYFVKNVDSFIITGFDHLQ--NQKLLGSLILISYMFILLGNGINLCIISTDKHL-HKPMYILICNLAVVDMMYSSTCSTTMISVLLA :  88
OR106-9  : MVN--------ETFVKNVDSFIITGFDHLQ--NQKLLGSLILISYMFILLGNGINLCIISTDRHL-HKPMYILICNLAVVDIMYSSTCSTTMISVLLA :  87
OR106-10 : MAN--------ETFVKNVDSFIITGFDHLQ--NQKLLGSLILISYMFILLGNGINLCIISTDRHL-HKPMYILICNLAVVDIMYSSTCSTTMISVLLA :  87
OR106-7  : MAND-------NYTVKKIDNFMITGFDHLQ--NQKLLGFLILISYMFILLGNGINLCIISTNRHL-HKPMYILICNLAVVDIMYSSTCSTTMISVLLA :  88
OR106-5  : MAN--------ETYMKNVDSFIITGFDHLQ--NQKLLGSLILISYMFILIGNIINLCIISTDRHL-HKPMYILICNLAVVDIMYSSTCSTTMISVLLA :  87
OR108-1  : MQN-------INSSIIQPAGFYIVALSSVP--YSNVYVMFLTVLYVITVICNVFLMSIIVYDQRL-HVPKFMAVGNLAVVDLILSTSLVPGMIKIYIV :  88
OR108-2  : MQNA--ISSGQNSSIIQPAGFYIVALSSVP--YSNVYVMFLTVLYVITVICNVFLISIIVYDQRL-HVPKFMAVGNLAVVDLILSTSLVPGMIKIYIV :  93
OR108-3  : MQNN--IAS-SNSTLIQPAGFYIVGLRSMP--YSNVYVMFLTVLFVITVICNVFLISIIVYDQRL-HVPKFMAVGNLAAVDLILSTALVPGMIKTYIA :  92
OR110-1  : MNQ----DFVGNGNVSTPSYFYISGFSGIP--HMRYYYIFLFFIYIITVLGNSCLMSVIIMDRNL-HTPKYIAVFNLSLTDICESTAVIPQLLDTFLF :  91
OR110-2  : MN-----DFAENRNISIPSYFYISGFSGIP--HMRYYYIFLSFVYIISFLGNSCLMFVIIIDRNL-HTAKYIAVFNLSLSDICESTAVIPQLLDTFLF :  90
OR109-1  : MS-----SHPANLTFVRPATFSLSGFSNMP--QAKYYYMFLSLVYAVTVLGNSFIMCIIYLARRL-HTAKYIVVFHLALSDLCGSSALIPKIIEMFLF :  90
OR109-2  : MSSIQ-SNSSANETFVRPATFFLSGLSNVP--HAKYYYVFLSLVYVATVLGNSFIMCVIYLSRRL-HTAKYVVVFHLAFSDLCGSSTVIPKIIDTFLF :  94
OR109-4  : MSFIQ-SNSSANETFVRPATFFLSGLSNVP--HAKYYYVFLSLVYVATVLGNSFIMCVIYMSRRL-HTAKYIVVFHLAFSDLCGSSTVIPNVIDTFLF :  94
OR109-3  : MSSIQ-SNSSANETFVRPAKFFLSGLSNVP--HVKYYYVFLSLVYVVTVLGNSFLMCTIYLARRL-HTAKYIVVFHLAFSDLCGSSAVIPKTIDTFLF :  94
OR109-5  : M---Q-SNSSANTTFVRPATFFLNGLSNVP--HVKYYYMFLSLVHVATVLGNSFLMSTIYLARRL-HTAKYIVVFHLAFSDLCGSSTVVPKIIDTFLF :  91
OR109-6  : MSSIQ-TNPSANLTFVRPAKFFLSGLSNVP--HAKYYYVFLSLAYVVTVLGNSFLMCIMYLSRRL-HTAKYIAVFHLALSDLCGSSTVIPKVIDTLLF :  94
OR109-9  : MSSIQ-TNPSANLTFVRPAKFFLSGLSNVP--HAKYYYVFLSLAYVVTVLGNSFLMCIMYLSRRL-HTAKYIAVFHLALSDLCGSSTVIPKVIDTLLF :  94
OR109-7  : MD----SNSSANVAFVRPATFFLNGFSNFP--HAKYYYLFLSLVYAVTVLGNSFIMCIIYLSRRL-HTAKYIAVFHLALCDLCGSSAVIPKVVDTFLF :  91
OR109-10 : MD----SNSSANVTFVRPATFFLNGFSNFP--HAKYYYLFLSLVYVVTVLGNSFIMCTIYLSRRL-HTAKYIAVFHLALCDLCGSSAVIPKVVDTFLF :  91
OR109-8  : M---Q-SNSSANVTFVRPATFFLSGLSNVP--HAKYYYVFLSLVYVVTVLGNSFIMYIIYLARRL-HTAKYIAVFHLAFSDLCGSSALIPKVIDTFLF :  91
OR109-11 : MNSIQ-SNSSANVTFVRPATFFLSGLSNVP--HAKYYYVFLSLVYVVTVLGNSFIMYIIYLARRL-HTAKYIAVFHLAFSDLCGSSALIPKVIDTFLF :  94
OR111-1  : MSSVN-ASFSKNISIVHPEYFFVIGLTGIG--YSSYYYIFLFITYFITVVGNTAVILIIVLDRRL-HSAKYIGMFNLALADFGEANALIPNMMKTFLF :  94
OR111-2  : MSSVN-ASFSQNISIVHPENFFIIGLTGIQ--HSGYYYIFLFFTYFISVIGNSVVLLIIALERNL-HSAKYIGVFNLALADIGETSALIPNMMKNFLF :  94
OR111-3  : MSSVN-ASFSQNISIVRPEYFFISGLSGIP--YSSYYYIFLFVVYFISVIGNSVVLLIIAVDRSL-HSPKYIGVFNLALADIGETNALIPNMMKTFLF :  94
OR111-4  : MNSVN-ASFYQNISIIRPEYFFISGLSGIP--FSNYYYIFLFVIYFISVIGNSVVLLIIAVDRSL-HSPKYIGVFNLALADIGETNALIPNMMKTFLF :  94
OR111-5  : MSSLN-ASFNQNISIVHPQYFFIIGLSGIP--YSSYYYIFLFVIYFITIIGNSLVLLIIALDRSL-HSPKYIGVFNLALADIGETNALIPNMVKTFLF :  94
OR111-10 : MSSLN-ASSYKNTSIVHPEYFFISGLSGIP--YSSYYYVFLFITYFITIIGNSVVLLIIAFERSL-HSPKYIVVFNLALADIGETNALIPNMMKTFLF :  94
OR111-7  : MSSLN-ASFSLNISVVRPEYFFILGLSGIP--YSNLYYIFIFIIYFITVIGNFLVILLIVLDRSL-HSPKYIGVFNLALADIGETNALIPNMMKTFLF :  94
OR111-6  : MNFLN-ESFYQNISFVHPQYFYIIGLSGTP--YSSYFYVFLFITYFITLFGNSVVLLIIALERSL-HSPKYIGVFNLALADIGETNALIPNMVKTFLF :  94
OR111-9  : MS------LYENNSIVRPEYFFILGLSGIP--NSSYYYIFLFIIYCITVIGNSLVLLIIALDRSL-HSAKYLCVFNLALADFGETNAVIPNMLKTFLF :  89
OR111-11 : MSSDS-ASFSSNISIVHPEYFFITGLSGTE--YSNYYYIFLFFLYIVAVIGNFIVLLIIALERSL-HSPKYIAVFNLALADIGEMNALVPNMIKMFLF :  94
OR111-8  : MS------LSQNSSSVYPEYFFIIGLSGIH--NSNYYYMFLCITYFVTVTGNSLVLLLIALYRSL-HSPKYIGVFNLALADFGETNALIPNMMRTFLF :  89
OR107-1  : MNLS---VTFSNGTASFNEGFYLTAFQTLP--TKNYLILGLAIIYIITLLGNLVLLSVVLMNSSL-QNPKYLAVCNLAVVDISINSVIIPQMVPVFVF :  92
OR104-1  : MSQ------ANDTTASTVTEFFIMGFPGLLPKYYSLTAALLFCIYIAVITGNSLIVVLFVIERSL-HKPMYIIMLSLSVSDICFCTVALPKVISRYWF :  91
OR104-2  : MSQE-----GNETRAFVVTEFFIMGFPGLLPKYYSLTAALLFCIYIAVITGNSLIVVLFVIERSL-HKPMYIIMLSLSVSDICFCTVALPKVISRYWF :  92
OR105-1  : MLS---------KNWTTISEFIIVGIPGLHPDYYGLVSAIFFVIYITTVAGNMFFLVLFVTTESL-RKPMYIILASLALSDMCFSTVALPKIISKYWF :  88
OR103-1  : MSA---------GNISFVKEFFIVGFPGLQKGYFDSVAALMLFVFVCILAGNGTFLILFSLEKSL-HKPMYYVILNLVASDILFSTTTLPKIIARYLF :  88
OR103-5  : MPV---------ENTSFVKDFVIVGFPGLQPHYYGLVSALLFFVYVFTLIGNAVFFTLFVITKSL-HKPVYYFIINLVVSDVLFSTATLTKIISRYWF :  88
OR103-2  : MSV---------GNTSFAKDFVIVGFPGLQPHYYGLVSALLFFVYLFTLIGNAVFFTLFVITKSL-HKPVYYFIINLVASDVLFSTTTLPKIISRYWF :  88
OR103-3  : MSV---------GNTSFVKDFFIVGFPGLQPHYYGLVSALLFFVYLFTLMENGVFFTLFVITKSL-HRPVYYFIINLVVCDVLLSTTTLPKIISRYWF :  88
OR103-4  : M-----------ENTSFAKDFFIVGFPGLQPHYYGLVSALLFFVYLFTLMENGVFFTLFVITKSL-HRPVYYFVLNLVVCDVLFSTTMLPKIISRYWF :  86
OR102-1  : MT----------AYYTNITEFYLLGFPGLHPEYYGAVGFLLFFVYLTLAGGNIFIIAFIAYEKSL-QKPTYLIFCNLAVCDIAIGTVTLPKAIAKYFF :  87
OR102-3  : MTNSVTPRVMTFTNITVFTEFYLLGFPGLHPDYYGALGTLFLFVYLTLLGGNIFIIAFIAYEKSL-QKPTYVIFCNLAVCDLTFGTVVLPQAIAMYFI :  97
OR102-4  : MANSVTPRVMTFTNITVFTEFYLLGFTEIHPDYYGAVGTLFLFVYLTLLGGNIFIIAFIAYEKSL-QKPTYVIFCNLAVCDIAAGTVVLPQAIAMYFI :  97
OR102-5  : MT---------FTNVTVFTEFYLLGFPELHPDYYGAVGTLFLFVYLTLLGGNIFIIAFIAYEKSL-QKPTYVIFCNLAVCDLTFGTVVLPQAIAMYFI :  88
OR102-2  : MGSR--SNVETYQNTTIITEFVLRGFPGLPPEYHGSVGALLFILYLVLATGNIFIIVFVMIEKSI-QKPTYIIFCNLAMADLALGTTTYPRVIAKYWM :  95
OR101-1  : MN-----------TSGSVEYFILDGL------KDKIVLPIFFTVYVSVLSGNSMIIYLVRTDPKL-DTPMYFFLHNLSFSDIVYTSVTIPKMLSVFLT :  80
OR113-1  : MKD-------LTAQNTSFTEFRLNGFYSLG-EWRPFLFIPFFLMFMLAIIANSILIYLIKTQRSL-HSPMYVLIGVMAVVDLIMPLSFVPNMLLSFLF :  89
OR113-4  : MKD-------LTPQNTSFTEFRLNGFYSLG-EWRPFLFIPFFLMFMLAITANSILIYLIKTQRSL-HSPMYVLIGVMAVVDLIMPLSFVPNMLFSFLF :  89
OR113-2  : MMN-------LTVQNTSFKDFRLTGFFSLG-EWRPLLFIPFFFMFLLAITANSILIYLIKTQKSL-HSPMYVLIGVMAVLDLILPIFFVPNMLLSFLF :  89
OR113-3  : MRD-------PPVQNTSFTDFKLNGFYSLG-DLRPFLFIPFFLMFSLAITANSLLIYLIISQRSL-HSPMYILIGLMGVVDLLLPVFFVPNMLLNFLF :  89
OR114-1  : MED-------KTFN-ISYQEFYLIGFTDLQ-DYRRYLFIPFFLIFAFTLFTNSIIILVIVKESKL-HAPMYILIGFVAALGFLQPIFAIPRILISFLF :  88
OR112-1  : MTEE------LQGANFSHTKFLLTGFPEIY-KYRRFLFLPFFLIYLLLLVGNSLLFVIKSTESL-HSPMYIFVSALAVVDIIVPTAIIPAMLLGFLF :  90
OR124-3  : ME-----------NISSSHILTVTALNDWDWTSRILFFSFALLVYLVTIVLNATMIMIIAFEKAL-HEPMYIFLCNLCINDLCGITGFYPRALAYLLT :  86
OR124-2  : ME-----------NISSSHILIVTALNDWDWTSRILFFSFALPCYLLTIVLNATLIMIIAFEKAL-HEPMYIFLCNLCINDLCGTTGFYPRALTYLLT :  86
OR124-1  : ME-----------NISSSHILTVTALNDWDWTSRILFFSFALPGYLLTIILNATLIMIIAFEKAL-HEPMYIFLCNLCINDLCGITGFYPRALTYLLT :  86
OR124-4  : ME-----------NISTSHILTVTALNDWDWTSRILFFSFALPGYLITIVMNATLIMIIAFEKAL-HEPMYIFLCNLCVNDLCGITGFYPKALVYLLT :  86
OR123-1  : MD-----------NNSSFSMFSLTALNGSRSS-RITIFSFALPGYFLTIFVNTLLILIIVLEKVL-HQPMYIFLCNLCINGLYGATGFYPPLLYYLLS :  85
OR126-1  : MLPL-------LKNETIVVVFTLSGLNDTM-GNRFVFFSLTALYYPFIVFCNVTILYAVMMHKKL-HEPMFFFICNLCVNALYGTVGFYPKFLYDLLS :  89
OR126-2  : MQPA-------LKNESSVLIFTLSALNETT-ENRYVLFSLTALYYPLIVFCNVTVIFTIISHKKL-HEPMYVFICSLCVNGLFGTVGFYPKFLYDLLS :  89
OR126-3  : MQSP-------LKNESNVFVFALSGLNETM-ENRYVLLSFTALFYPVVIFCNVTVILTIFLHKKL-HEPMYIFLCNLCINGIFGTSGFYPKFMYDLLA :  89
OR126-5  : MQQQ-------LENESSVSVFKLSGLNETM-ENRIVFFSFTALFYPLMVFCNVTVMFTIMSNKKL-HEPMYMFICNLCVNGLFGTSGFYPKFLYDLLA :  89
OR126-4  : MQSL-------WKNESSVSVFTLSALSETM-ENRYVLLTFTALFYPLVVFCNVTVILTIFLHKKL-HEPMYVFICNLCVNGVYGTSGFYPKFMYDLLA :  89
OR125-1  : MD-----------NMSYPVILTLMVPKETK-LYRHIYFICFLVLYLLILSINICLVMVIIIEKVL-HEPMYIFLCHLCINGVYGATGFYPKILSDLIL :  85
OR125-2  : MD-----------NMSYPVILTLIIPKDSK-SYRHVYFICFLVLYLLILSINIRLVVVIVMEKVL-HEPMYIFLCHLCINGVYGATGFYPKILSDLIL :  85
OR125-8  : MD-----------NRSYPVILTLMVPKDSK-SYRHVYFTCFLLLYLLILSINIRLVMVIIMEKAL-HGPMYIFLCHLCVNGVYGATGFYPKFLSDLIL :  85
OR125-3  : MG-----------NITYFDSYTLMELQSSK-SYRHIYFACFFLLFCLICLLNSCLCVVIVMERAL-HQPMFIFLCNLCINGIYGATGFYPKLLHDLQL :  85
OR125-5  : MD-----------NMTHFETYTLMEPLNAK-SYRYIYFMCFFLLYALVLLVNIWLIVVIVIERAL-HEPMYIFLCNLCVNDIYGCLGFYPKFLHDLIL :  85
OR125-6  : MD-----------NMTHFETYTLMEPLNAK-SYRYIYFMCFFLLYALVLLVNIWLIVVIVIERAL-HEPMYIFLCNLCVNDMYGCVGFYPKFLHDLIL :  85
OR125-7  : MD-----------NMTNFETYTLMEPLNAK-SYRYIYFMCFFLLYALILFVNIWLIVVIVIERAL-HEPMYIFLCNLCVNDIYGCVGFYPKFLHDLIL :  85
OR125-4  : MD-----------NQTVFASYTLMVPRDSK-SFRHIYFICFLVLYVLIILVNAWVSTVIVLERTL-HQPMYIFLCNLCVNDIYGAAGFYPKFLHDLML :  85
OR128-13 : MDN------------GTYFYFMFFEN---LG-YIRYALFTSGLILYCVIILFNAVIIITIFWNRTL-HQPMYILISCLSVNSVYGTAGFFPRVLTDLLS :  82
OR128-7  : MDN------------GTYFYFMLFEN---LG-YIRYALFTSGLILYCVIILFNAVIIITIFWNRTL-HQPMYILISCLSVNSVYGTAGFFPRVLTDLLS :  82
OR128-9  : MDN------------GTYFYFMLFEN---LG-YIRYALFTTGLILYCVIILFNAAIIITIFQGPSL-HQPMYILISCLSVNSVYGTAGFFPRVLTDLLS :  82
OR128-8  : MDN------------GTYFYFMLFEN---LG-YIRYALFTTGLILYCVIILFNAVIIITVFCGRTL-HQPMYILISCLSVNSVYGTAGFFPRVLTDLLS :  82
OR128-6  : MDN------------GTYFYFMFFEN---LG-YIRYALFTSGLILYCVIILFNAVIIITIFWNRTL-HQPMYILISCLSVNSVYGTAGFFPRVLIDLLS :  82
OR128-10 : MEN------------GTTFYLMLFEN---IG-CTRYALFSLGIILYSAILLFNFLVILAIFLERTL-HQPMYILISCLSFNSVFGTAAFFPRLLTDLLS :  82
OR128-1  : MEN------------NTNFNFMLFEN---LG-YIRYALFILGFVLYFSIIFFNVLIMLAVFLERTL-HQPMYILISCLSMNSLFGTAGFFPRVLSDLLS :  82
OR128-2  : MEN------------NTSFNFMLFEN---LG-YIRYALFILGFVLYFAIILFNVLIMLAVFRERTL-HQPMYILISCLSMNSLFGTAGFFPRVLSDLLS :  82
OR128-3  : MEN------------YTSFNFMLFEN---LG-FIRYALFILGFVLYFAIILFNVLIMLAVFLERTL-HQPMYILISCLSMNSLFGTAGFFPRVLSDLLS :  82
OR128-4  : MEN------------YTSFNFMLFEN---LG-YIRYSLFVLGFVLYFAIIFFNVLIMLAVFLERTL-HQPMYILISCLSMNSLFGTAGFFPRVLSDLLS :  82
OR128-5  : MEN------------NTNFNFMWFEN---LG-YIRYALFILGFILYFVIILFNVLIMLAVFLERTL-HQPMYILISCLSINSLFGTAGFFPRVLSDLLS :  82
OR127-1  : MEN-----------GTMSSSFYFTLFKEFVH--IRYVILTLTLTVYLAIIVCNAIILFVVFKERSL-HEPMYILISCLSVNDLYGSAGFFPRLIADLLS :  85
OR122-2  : MDN------------STYFTTVILTGYIDIG-DIKYLCFVVLILLFAAIIVANLFIIGIIYKEKSL-HEPMYVFLCSLCANQLCGSFALFPFVLGNMLR :  85
OR122-1  : MDN------------STYFTTVILTGYIDIG-DIKYLCFVVLILLFAAIIVANLFIIGIICKEKSL-HEPMYVFLCSLCANQLCGSFALFPFVLGNMLR :  85
OR121-1  : MEN------------GANFTFIRLTGYLNLDFKMKYFLFAVLTVLYSTIIFANSFLIGVICLAKIL-HKPMYMFLCSLFVNELYGSTALFPSLQVNLLL :  86
OR120-1  : MS-------------LSSLNITLFFTGYGPPSALNYGVFVISLVVYLTTVVANVTLMLVICLDASL-HKPMYVFLFNLAINGLIGSSAVLPKIMDNLVD :  85
OR115-14 : MD-----------NQTFQYSKLLVEGLQVTSQSTLPTFILLLMVYAFIMVSNIGMMVLISADKCL-HQPMYVLFCNLTLNDILGTTDFVPPVLRNLLI :  86
OR115-2  : MD-----------NQTFQYSILLVEGLQVTSQSTLPTFILLLIVYVFIMVSNIGMMIQISADKCL-HQPMYVLFCNLTLNDVLGTTVFMPHVLKNLLK :  86
OR115-13 : MD-----------NQTFQYSKLLVEGLQVTSQSTLPTFILLLMVYAFIMVSNIGMMIQISADKCL-HQPMYVLFCNLTLNDVLGTTVILPGLLRNILT :  86
OR115-12 : MD-----------NRTFRYSTLLVEGLQITQQSSQLTFIFLLTVYVFSMVSNIGILIQILAEKSL-HQPMYILFCHLPMSDIIGATVLIPRLLKDLLT :  86
OR115-10 : MD-----------NQTFRSSTLLMEGLQVANQYRQLAFIFVLIAYIFIMIFNIGLLIQISSEKSL-HQPMYILLCNLPLNDVFGTTLLLPRLLRNILL :  86
OR115-11 : MD-----------NLTLRYSVLLVEGLSVSPPYSYVAFFSLLTVYIFIMVSNLWILLNIMIDKDL-HQPMYSIYSNLPLRDVIGTSVIVPRLLRDVLT :  86
OR115-9  : MD-----------NLTFRHSVLLVEGLKVTHQSSQPVFIVLFLSYIFAMISNITLIFLISTEKNL-HDPMHFLFCNLPVNDIIGTTVILPRLMQDVLK :  86
OR115-7  : MD-----------NLTFTNSILLVEGLKVTPQSSQPVFIMFLLAYIFAMVSNIGLIFLISTEKNL-HDPMHFLFCNLPLNDIMGTTVIMPRLLQDILT :  86
OR115-4P : MD-----------NLTITNNILLVEGLKVTPQSSYPVFVLLLLVYIFAMGCNIGLIILISTEKNL-HHPMHFLFCNLPLNDILGTTVMLPRLLQDILR :  86
OR115-1  : MD-----------NLTITNYTLLVEGLKVAPQSSYPVFILLLLVYVFIMVSNIGLIVLISTEKNL-HHPMHFLFCNLPLNDIIGTNVILPRLMQDMLR :  86
```

Figure S1

```
                                                   ⇓______________TM1______________        ______________TM2______________

OR115-6  : MD-----------NLTITNRILLIEGLKVTQHSSYPVFILLLLVYLFIMGCNIGLVIVILTEKNL-HHPMHFLFFNLPLNDIIGSTVIMPRLLQDILR :  86
OR115-8  : MD-----------NLTLTNNVVLVEGLKVTLQSSYPAFILLLLIYVFTMGCNIGLTILILTQKNL-HNPMHFLFCNMPVNDIIGSTVIMPRLLQGILR :  86
OR115-5  : MD-----------NLTFTNDFLLVEGLKVTYPSSYPIFILLFLFYIFAMGCNIGLIILIATEKKL-HHPMHFLFCNLPVNDIIGTTVILPRLICDIFK :  86
OR115-3P : MDL----------NETFRHSILLVEGFRVTPQSTYPVFILLLLAYVFTMVSNIGLIVLISTEKNL-HHPMHFLFCNLPLNDIIGTTVIMPRLMLDILK :  87
OR116-1  : MEN----------LSIIVSFELTLDPFFIPPGAKYPIFIFGIFIYMFAAFCNLTLLLLIILTKSL-HKPMYFILFSLPLNDFVGITSMLPKVLSDILT :  87
OR116-2  : MEN----------LSIIVSFELTLDPFSIPPGAKYPIFIFGIFIYMFGAFCNLTLLLLIFLTESL-HKPMYFILFSLPLNDFVGITSMLPKVLSDILT :  87
OR117-1  : MAG--------NLSNPTFTFYLKIAKFDIDPKAKYPVFFAGILVYMFSVICNTTILGLIISQKSL-HKPMFYILFSLPLIDLIGITAGLPRVLVDIVT :  89
OR118-1  : MYP----------NESVFSLVLTLHSLELPEANIYPAFIFGLTTYLFILLCNLTIIITICLNRNL-HKPMYILLLNLPINDVLGATCLFPQLLYSIWS :  87
OR118-2  : MYP----------NGSVFPLVLTLQSLELPQMSIYPAFIFGLTTYLFILFCNLTIVITICLNRSL-HKPMYILLLNMPINDTMAATNFFSQLLYSIWS :  87
OR118-3  : MYP----------NESVLSVVLTLHSLELPQMSIYPVLTFGIMTYLIVLLCNLTIIVTICLNRNL-HKPMYILLLNLPINDTMGSTSLFPHLLYSIWS :  87
OR119-1  : MSS-A-------VDVYNVSSDLSLQGFDLSPEKAIIAFFFAIVNYIIILLCNSFLLFTIISNKAL-HEPMHILLLNLPINDLIGSTCLFPHVMRELLF :  89
OR119-2  : MSS-A-------VDVYNVSSDLSLQGFDLSPEKAIIAFFFAIVNYIIILLCNSFLLFTIITNKAL-HEPMHILLLNLPINDLIGSTCLFPHVMRELLF :  89
OR132-5  : MAGSN-------GTAEDSSFTYQQIFKLDMDLVTSTKTAVAVLTSLFFFFVNCVMFFALKSKHIFFETPRYILFGHMLMNDSVLLLVTTVMYVVALCF :  91
OR132-1  : MAGSN-------GTYGNVPFTHQQVFKLDMDAGTSTKTAVAVLTSLFFFSVNCVMISVLKSKRMFYETPRYILFGHMLMNDSVLLLVTTVMYTLALCF :  91
OR132-2  : MD-SN-------GTSGDVSFLYQQVFQLDWDPLTICETILAVLTSLFFFFVNCVMFLALKSKHIFFETPRYILFGHMLMNDSVLLLLTTVMFMLALCF :  90
OR132-3  : MD-SN-------GTSGDVPFLYQQVFQLDRDPLTICETILAVLTSLFFFFVNCVMFLALKSKHIFFETPRYILFGHMLMNDSVLLLLTTVMFMLALCV :  90
OR132-4  : MD-SN-------GTSGDASFLHQQVFQLDWDFLTISKTMLAVLTSLFFVFVNCVMFYALKSKHIFFETPRYILFGHMLMNDSVLLLLTTVTFMLALCF :  90
OR133-1  : M-----------SNSNVTNSNITQ---TITPVDRDLKTILSVSSCVIFLYVNGVMIFTLRKKPVFQETSRYILFGHMLWVDTLNLVMSVVLFACAVFR :  84
OR133-2  : M-----------SNINGSTSNLTQT-LSLVDRDAPVKTFLCVTPCVVFLYVNGVMMFTLRKKTVFLETSRYILFAHMLWLDTLHLFMSVVLFVCAVSR :  86
OR133-3  : M-----------SHLNESTSKLTQ---RLMEQFAPVRTFLIVTPCVLFLYVNAVMIFTLRKKTVFQQTSRYILFGQMLWVDTLNLFMSVVMYVCAASG :  84
OR133-4  : M-----------STLNGSTSNNTQ---SLMDQFAPMKAFLLVTPCVIFLYVNGVMIFTLRKKTVFQQTSRYILFGQMLWVDTLNLFMSVLLYLCAISR :  84
OR133-6  : M-----------SNLNESTSNNTQ---RLMDQYTEVRTFLIVTPCVIFLYVNGVMILTLRKKAVFQQASRYILFGQMLWVDTLNLFISVVMYMCAVSR :  84
OR133-5  : M-----------SSLNGSSSNNTQ---RLMDQYVPLKTFLLVTPCVIFLYVNGVMIFTLRKKTVFQQTSRYILFGQMLWVDTLSLLMSVLLYLCAISR :  84
OR133-7  : M-----------SNLNASASNLTQ---RLMDQDAPTKAFLFVTPCVIFLYVNGVMLFTLRKKAVFQETSRYILFGHMLWVDTLNLFMSVVLYICAVSR :  84
OR133-8  : M-----------SNFNESASNLTQ---RLIDQNGPTKAFLIVTPSVVFLYVNGVMIFTLRKKAVFQEMSRYILFGHMLWVDTLNIFMSVVLFICAVSR :  84
OR133-9  : MK--M-------SNFNESASNLTL---GLIDQNTPMKASLIVTPSIVFLYVNGVMIFTLRKKAVFQEMSRYILFGHMLWVDTLSLIFSLSVYICAVCR :  86
OR137-7  : M--------------NSTVLLYSQ---QERYADAFAKNFSTVLLAVIIISINGMFVFIFFRRSIFHSDPRYILYIHLVMNDTLMVFISVVLFVMTFAW :  81
OR137-1  : M--------------NSTVLLYSL---QERYSDVFAQNFTVVCLAVIIISINGMFVFIFFRRSIFHSDPRYILYIHLVINDMLMVFTSVILFVMAFIW :  81
OR137-2  : MTA-----------TNSTRFLYSL---QEQYSDSFAKNFTVVLLAVIIVSVNGMFVLTFFRSSIFYTDPRYILYIHLVMNDMLMVCITVILFVMAYAW :  84
OR137-3  : M--------------NSTMFTYTN---VDRYAEAFAKNFTTVLLGLIITCVNGLFVFTFFKSSIFYNDPRYILYVHMVICDMLMVIFSVGLSVMAYAW :  81
OR137-8  : M--------------NSTMLEYNA---QDRFRDALVRNVIVVIFGITINSINGVFVFTFFRSSVFXEDPRYILYIHLVINDMLMVFVSVSLSVMAYAW :  81
OR137-4  : M--------------NSTMLQYNA---QDRFKDALVRNVIVVVFGITINSIIGLFVFTFFRSSVFYNDPRYILYIHLVINDMLMVFLSVSLSVMAYAW :  81
OR137-5  : M--------------NSTVLQVNA---QDRFRDALVRNFIVVIFGITINSINGVFVFTFFKNSVFYNDPRYILYIHLVINDMLMVFVSVSLSVMAYAW :  81
OR136-2P : MN-----------------STIWI---VDSYEEALAKNIVIVSLGFIINVINGMLVVTFFSNPMFSRDSRYILYIHLVINDMLMICVSVILYVLTYTA :  78
OR136-1  : MCSCL-------FSSTEMNTTVWI---VDNYQEAITKHIVIVFLALIVNVINGMLVVTFFSSHVFSRDSRYILYIHLVINDMLMICASVAIYVLTYIA :  88
OR135-2  : MN----------------SLELNTTRRKDFLWEAIFKNIIVVGLGIIINFINSLLFYTLFKNPVLMQEPRYILYMQLIINDIILLSVCVTLFVITNSL :  82
OR135-1  : MN-------------SSNNKQ-------DFLGDCVFKSFFVVLLGVIINCINSILLYTFFKNPVLAQEPRYILYTQLIINDIITLSVGVTLYVFTCLL :  78
OR140-1P : MNN----------TLPADPFY-----------EKFIKNFISVALGLIINFINGTFAMVFFKNTVFHNDPRYILYLHLVINDMILLSFFVGLQVTTYVF :  77
OR134-1  : MN-------------STAGQQLLL---RDTFAMAFMKNFIVVLVWFLLSYINGSVVATFFRHQIFYEDPRYILFIHMVINDAVQLTVTIALFLVSYIL :  82
OR129-1  : MQN--------------LTELPANATTSSSNLSVIVKVCVVMPIFIAFLFFILLTLYTFASHRQFFDSSRYVLFTYMLINDTLLLFSSVALFLLAMAG :  84
OR131-1  : MN-------------STSNSSLSN---TFIMKSLKEKALLVQVLVGILLYVNGLMIFTFLKKETF-RDTRYILFAQTLFVDSSFMIFADLTLLCSAYQ :  81
OR131-2  : MN-------------STSNSSLGN---TFISKTLKEKSLTVQVLVGILLYVNGLMIFTFLKKETF-RDTRYILFAQTLFVDSALMLFADLTLVGSAYE :  81
OR130-1  : MS------------SYNSSGDEQMQLIQVDPVRRNISLALTQVIVWPFIYLIFLMLLIFSKKETFTTEMRYILFGHSLLVDLTFLLLTDFVVLLSYNL :  86
```

Figure S1

```
                                             TM3                                TM4
             100          *        120         *        140         *        160         *        180         *
OR106-12  : EI--KTVSYYSCISRTYFYHLGGFTECMALTLMAIDRLIAIRLPLRYHSIVTNSRVFLLIFLTWLAGFAVIGYVISILDNVPYCQPI--IKYVFCEYA : 182
OR106-11  : EI--KTVSFYSCISRTYFYHVGGFTECMALTLMAIDRLIAIRLPLRYHSIVTNSRVFLLIFLTWLAGFAVIGYVISVSDNVPYCQPI--IKYVFCEYA : 181
OR106-1   : DV--KTVSYYSCISRMFFYHIGDFTECMALTLMAIDRLIAIRLPLRYHSIVTNSRTFLFIVLTWVIAIALMGVLTSAVDNAPYCQPV--IKYVFCDYP : 180
OR106-2   : DV--KTVSYYSCISRMFFYSLGDLTICMALTLMAIDRLIAIRLPLRYHSIVTNSQTFFFILLTWIIAIALIGVMTSAVDNAPYCQPV--IRYVFCEYA : 182
OR106-3   : ED--KTVSYYSCISRMFFYHLGDYTICLALTLMAIDRLIAIKLPLRYHSIVTNSQTLLFIVLTWIIAIAIMGVLTSIIDNAPYCQPI--IKYVFCDYP : 182
OR106-4   : EV--KTVSYYSCILRMYFYHLGDLTECMALTLMAIDRVIAIRLPLRYHSIVTNSRILWLIFLTWMFCFAAIGYLILLADSLPYCQPV--IKYIYCSYA : 182
OR106-6   : EI--KTVSYYSCISRMFFYTLGDLTECMALTLMAIDRVIAIRLPLRYHSIVTNSRILWLIFLTWMFCFAAIGYLILLADSLPYCQPV--IKYIYCSYA : 182
OR106-8   : EI--KTVSYYSCISRTYFFNLGDLTESMALTLMALDRLIAINLPLRYNSFVTNSRILWLVFLTWILSFAVIGFLIYVTDSLTYCQPI--IKYVFCSYS : 182
OR106-9   : EI--KTVSYYSCISRTYFFNLGDLTECMALTLMALDRLIAISLPLRYHSFVTNSRILWLIFLTWILSFALIGFLTYVADTLPYCQPI--IKYVFCSYS : 181
OR106-10  : EI--KTVSYYSCISRTYFFNLGDLTECMALTLMAIDRLIAINLPLRYHSFVTNSRILWLIFLTWIFSFAVIGFLTYVADTLPYCQPI--IKYVFCSYS : 181
OR106-7   : EV--KTVSYYSCISRTYFYHLGDLTECMALTLMAIDRIIAIKLPLRYHSIVTNVRIVLLIFLAWLICFVAVGFLTYVADSLTYCQPV--IKYVFCSYS : 182
OR106-5   : EI--KMVSYYSCITRTFFYHVADIAECMALTLMAIDRVVAIRLPLRYHSIVTNLRTFCFIFVTWLMGFAAVGFFTSVLDKSPYCQPL--IKYAFCSYS : 181
OR108-1   : LD--NFMPFKLCLVQMYTYYTFLELETLSLCILSYDRFIAICFPLRHESINTNTRMTYIICVSWFLSFSISLYGTLSIPALSFCGSLQ-VNSYFCDYA : 183
OR108-2   : LD--NFMPFKLCLVQMYTYYTFLKVEALSLCILSYDRFIAICFPLRHESINTNTRMTYIICVSWFLSFSISLYGTLSIPALSFCGSLQ-VNSYFCDYA : 188
OR108-3   : LD--NFVPFNLCLLQMYIYYTCTCLESFSLCILSYDRFIAICFPLRHESINTNSRMAYIISAVWFFCVVTLLYAVFSILTLSFCGSNE-MNSYFCDYT : 187
OR110-1   : GN--QMMPYGLCMSSMFSVLLFLAMQSLTLTVLSFDRFVAISLPLRYHMIVTQRLMLIIIAASWTLALLLMIIGAIFMSRLSFCKVIVTVNSFYCDHG : 187
OR110-2   : RN--QLIPYRLCLYNMFSVFVSISTQSLTLAVLSFDRLVAICLPLRYHMIVTFRSMLVIIGLTWALTLLMVMTATIFISRLSFCKVIVTVNSFYCDHG : 186
OR109-1   : DH--QDISYEACLTNMFFVYHFMNLQSLTLLALAYDRLVAICFPLQYHAIVTKSAMFLIIGVMWIVSVTFFSILVGLVNTISFCRSNV-LNSYFCDYA : 185
OR109-2   : EH--QEISYEACLANTFFIYHFLNIQSLTLLVMAYDRLVAICFPLRYHAIITKPTMFLILGGMWIYSVTYFSVFIGLLNRLSFCKSNV-IDSYFCDYS : 189
OR109-4   : DH--QEISYEACLANTFFTYHFMNLQSLTLLVMAYDRLVAICFPLQYHAIVTNAAMFSILGGMWIFSVTHFSVFVGLLNRLSFCKSNV-IDSYFCDYS : 189
OR109-3   : DH--QDISYEACLANTFFIYHFLNIQSLTLLVMAYDRLVAICFPLHYHAIITKAAMFLILGGMWIFSVTHISVFVGLLNRLSFCRSTV-IDSYFCDYS : 189
OR109-5   : EH--QDILYEACLANMFFVYFFMSMQSLTLLILAYDRLVAICFPLRYHAIVTKPSMFLIIGAMWIFSLTYFSVLVGLVNRISFCGSNV-IDSYFCDHG : 186
OR109-6   : DH--QDVSYEACLTHMFFIYHFMNLQSLTLLVMAYDRLVAICFPLRYLAIVTKPSMFLIVGVMWIFSVIYFSVLVGLVNRISFCGSRV-IDSYFCDQG : 189
OR109-9   : DH--QDVSYEACLTHMFFIYHFMNLQSLTLLVMAYDRLVAICFPLRYLAIVTKPSMFLIVGVMWIFSVIYFSVLVGLVNRISFCGSRV-IDSYFCDQG : 189
OR109-7   : DH--QDISYAACMANIFFVFHFMNVQSLTLLVMAYDRLVAICFPLRYHAIVTKTSMLLILGVIWTFSLIFFSVFVDSINRLSFCRTNV-INNYFCDYC : 186
OR109-10  : DH--QDISYEACMANIFFVFHFMNVQSLPLLVMAYDRLVAICFPLRYHAIVTKTSMLLILGVIWTFSLIFFSVFVDSINRLSFCRTNV-INNYFCDYC : 186
OR109-8   : DH--QDISYEACMANMFFVLHFMNMQSLTLLALAYDRLIAICFPLRYHAIINKSAMFLIIGVMWIFSATFFSVVVGSVSRISFCISNV-IDTYYCDFA : 186
OR109-11  : DH--QDISYEACMANMFFVLHFMNMQSLTLLALAYDRLIAICFPLRYHAIINKSAMFLIIGVMWIFSATFFSVVVGSVSRISFCISNV-IDTYYCDFA : 189
OR111-1   : DS--QYIYYNACMANMFFVFLFSTMQSFTLVVMAYDRLIAICLPLRYHSIVNNSNMILIFSAIWAFNSSVVALMVSLIDRLSFCESNM-IQSYFCDHG : 189
OR111-2   : DS--QYISYNGCMANMFFVFLFSSMQSFTLVVMAYDRLIAICLPLRYHAIVNNTSMILMTLAIWAFNTFVVALMVSLINRLSFCESNM-IQSYFCDHG : 189
OR111-3   : NS--QYISYNGCMANMFFVFLFNSIQSFTLVALAYDRFIAICLPLRYHAIVNNTSMILIFLAIWAFNSSVVASMVSMITRLSICKSNV-IPSYFCDHG : 189
OR111-4   : DS--QYISYNACLANMFFVFLFSSVQSFTLVALAYDRFIAICLPLRYHAIVTNTSMILIFSTIWVFNSSVVASMVSMIPRLSFCDSNK-IPSYFCDHG : 189
OR111-5   : DS--QYISYNACLANMFFVFFFSSLQSVSLVIMSYDRLIAICLPLRYHSIVNNSSMILVSPAIWIFNSSMVALMVSLITRISFCDSNV-IQSYFCDHG : 189
OR111-10  : DS--QYISYNACLVNMFFVFLFSTMQSFTLVVLAYDRFVAICLPLRYHAIVNNSSITLILSAKWAFNSSMVALMVSLITRISFCDSNV-IQSYFCDHG : 189
OR111-7   : DS--QYISYNACLVNMFFVFLFSSMQSLTLVVMAYDRLIAICLPLRYHVIVNNTSMISIFSAVFMFNSIIVASMVSLVTNISFCKSNV-IQSYFCDHG : 189
OR111-6   : DS--QYISYNACLANMFFVFLFSSIQSFALLVLAFDRFVAICLPLRYHALVNNTTMLLAFAAVWAFNSFVVALMVSLITRLSFCQSNM-IQSYFCDHG : 189
OR111-9   : DS--QYISYNACLANMFFVNYFINVQSATLVVLAFDRVIAICLPLRYHSIVNNIVMLIVFFGIWTLNTLVVALSVSLMTRLSICKSNV-VESYYCDYG : 184
OR111-11  : ES--QYISYNACMVNMFFVHFFTTMQSFTLVVMSYDRFVAICLPLRYHAIVNNTVISVVFLVLWAFNVFVLLVVVGMITRLSICKSNV-ILSYYGDHG : 189
OR111-8   : DS--QYISYNACLANMFFVHFFSTVQSFTLVVLSYDRFIAIFLPLRYHAIVNYTFMYFVFSIIWVFNASIVAFAVSLITRLSFCESNK-VNSYYCDYG : 184
OR107-1   : NQ--NYISFGACFSQMFFMHFFGDMESFSLALLAYDRLIAICWPLRYSTINTNLRMLLIIALIWILVTLLDIFPVIFASRLPYCSSRA-VLSCCCEHG : 187
OR104-1   : ND--GFISFYVCLFQRQLIHYFGTLNSLIMMLMALDRYLAICHPLRYPVLMTNRTMRLLVGFSWVTAMIAPSISLSLTLKLPFCGPNM-IAHCFCDSL : 186
OR104-2   : ND--GYISFYVCLFQRQLIHYFGALNCLIMMLMALDRYLAICHPLRYPVLMTNRMLGLLLGFSWVTAMIPPTITLTQTLKLPFCGPNI-IAQCFCDST : 187
OR105-1   : NA--GATPFHACFFQMELIHYFGSLNSLIMMIMALDRYVAICYSLRYQTVMTNRVTYILITTAWITAFIAPTVATLDTQQLPYCGPNL-ILQCYCDHI : 183
OR103-1   : QD--GSISFMGCFIQMYFVHYFGTVNAFILAIMAFDRYVAICNPLRYPNIVTKWNIFYLCLISWVIANVTPLMMVIRAYPLPYCAENT-IIQCYCDHI : 183
OR103-5   : QD--GTISFLGCFVQMFFVHLFGSVCSVVLAVMAIDRYTAICYPLQYHSIMTNRNVLILILSPWILSFWGPLALVIRAYPLPYCADNS-IIHCYCDHV : 183
OR103-2   : QD--GTISFLGCFVQMFFVHFLGSACSVVLAVMAIDRYTAICYPLQYHSIMTNRNVLILILSPWILSFLGPLALVIRAYPLPYCADNS-IIHCYCDHV : 183
OR103-3   : QD--GTISFLGCFVQMFFVHYLGGVISNVLAVMAIDRYAAICYPLQYHNIMTHRNMLILILGCWLLSLAGALMLVIRAYPLPYCAENT-IIHCYCEHV : 183
OR103-4   : QD--GTISFLGCFVQMFFVHYLGGVISNVLAVMAIDRYAAICYPLQYHNIMTNRNILILILGCWILSLAGPLMLVIRAYPLPYCAENT-IIHCYCEHV : 181
OR102-1   : DS--SEITFLSCFVQMFFVHYLGSINSFLMLLMAVDRFVAVCNPLRYCVLITNKTILLACALIWMLCISWLTFMVYNTFDEPYCGSNI-ITQNYCDHN : 182
OR102-3   : NI--NTISFNLCFVQMFFLFCLSNVSSFIILLMTIDRFVAIRNPLRYCVLITNKTVFIACGVIWTFITPLMAFIVYQSYDEPYCASNV-VTQILCERN : 192
OR102-4   : NI--NTISFNGCFVQMFFVHYLGSVSSLIILLLTIDRIVAIQNPFRYCVLITNKTIFIACGVIWTAITPLMAIIVYEAYDEPYCATNV-VTQIFCERN : 192
OR102-5   : NI--NTISFSRCFVQMFFVHYLGSVSSLIILIMTIDRIVAIQNPFRYCVLITNKTIFIACGVIWTAITPLMAIIVYEAYDEPYCATNV-VTQIFCERN : 183
OR102-2   : SD--KIISFSGCFTQMFFIHFLGATNSFLMALMALDRFIAICNPLRYHTLIKNSTILIICAFVWTANMLQLVGVTLLSLSVKYCGSNV-IPHCYCDHV : 190
OR101-1   : GD--KTISKTGCFMQIQFVLWMGLTGRGLLTAMAFDRYVAICNPLRYTTIMTRKLCVLLIFASWTYGALIVLPPVIWATQLPYCGPNV-VKHMFCDHS : 175
OR113-1   : NW--SGISLTGCLMQMFGIHFVGSFQVTLLFWMALDRYFAICKPLYYHKYMEMSNFLKFVFAPVIRNALLIVTMVSLAGRLSFCATNI-IDHSFCEHM : 184
OR113-4   : NW--SGISLTGCLIQMFGIHFVGTFQNTLLLWMALDRYFAICKPLYYHKNMEMTNFLKFVAVPVIRNVLLIIIMVSLAGRLSYCLTNV-IDHCFCEHM : 184
OR113-2   : NW--SGISLPGCLIQMFGLHFIGTFQSTLLFWMALDRYFAICKPLYYHKSMEILNFLKFVFAPVIRNGLLTVTMVSLAGRLSFCATNV-IDHSFCEHM : 184
OR113-3   : NW--SGISLTACLIQMFCLHFVGTFQTTLLLCMALDRYFAICKPLYYHKYKEISNFLKFVFAPVIRNALLIVIIVSLAGRLSFCATNV-IDHCFCEHM : 184
OR114-1   : GR--NMIYKSECLVQMFCLHMAGCFQSTVLVAMAVDRFFAITFPLQYHYYVNLKTSLIFGLALFLRNLVVVVSMVGLIAPLQFCKTNV-IYHCVCEHT : 183
OR112-1   : DL--NEITLSGCLTQMFVTHFFSSVESTILLAMALDRFVAICKPLHYNEIMNSSMFLKLFLFTLIRSGTIMSTLVALASPLSFCGSNV-IYHCYCDHM : 185
OR124-3   : ER--NVISHEECIVQDLVIIVYGVGEFTSLSVMAVDRYIAICRPLHYHSIMSPFTVLCLLFFIWVFSSSVGLMAISLTLKYPICRHD--INKLFCEHL : 180
OR124-2   : ER--NVILHAECIVQGFVIIVFSIGEFTNLSVMAVDRYIAICRPLHYHRIMSPFTVLSLVTFIWVFPCSVGAMSISLTLKYPLCRHD--INRIFCENL : 180
OR124-1   : ER--NVISHEECIVQGLVIVIYATGEFTSLSVMAVDRYIAICRPLHYHRIMSSFTVLSLVTFIWVFSCFVGLMAISLTLKYPICRHN--INKLFCEHL : 180
OR124-4   : ET--NRISLEECIVQGLVIVIYAVGEFSNLSVMAIDRYLAICRPLHYHSIMSPFTVLCLLSFNWVFPFIASLVAIVVAMKNPICKYD--IDKLFCENL : 180
OR123-1   : ES--NVISLKECAIQSFAIFTYAIGEFTNLSVMAFDRYLAICRPLYYHNIMTTVTVWRLLTFIWMFPCCTAILLILMTLRFPICIKK--ISKLYCETW : 179
OR126-1   : QD--HVITYAGCLIQIFIIYSSVLCDISTLTVMAYDRYVAICRPLEYHSIMTDQRIVECILFCWLTPFFCMTVLIGLTARLTLCGSA--IEKLYCENW : 183
OR126-2   : QD--QVISYVGCLIQIFVIYSSLLCDFSTLTVMAYDRYVAICRPLEYHSVMTARMVFKCILFCWLPPMSCLCLLVTLTSRLTLCGSS--IEKLYCENW : 183
OR126-3   : PE--HVISYAGCLIQIFVIYSSVFCDFSTLTVMAYDRYVAICRPLEYHSMMTNQKILKCVLFCWLSPFFCMAVLILLIARLTLCGFR--IEKLYCEIW : 183
OR126-5   : HD--HVISYVGCLIQIFVIYSSALCDFSTLTVMAYDRYVAICRPLEYHSIMTSRMILKCILFCWLAPFVCMSVLIVLIARLTLCAST--IEKLYCEIW : 183
OR126-4   : QD--QVISYAGCMIQILVIYSYLICDFSTLTVMAYDRYVAICRPLEYHSIMTNQKIFKCILFCWLPAIFSISFLILLLVRLPLCGST--IEKLYCEIW : 183
OR125-1   : DS--YVISSHMCALQTYVIYSSLLSEITILTVMSYDRYVAICKPLDYHSRLTKNTCVKLILFSWIVPTSFSVASILLSNLRAFCKYH--IDKLYCDNW : 179
OR125-2   : DS--YVISSHMCALQIFVIYSSLLCEFTILTVMSYDRYVAICKPLDYHTRLTKNTCVKLILFSRIAPTFSVFIGTLLSNVRPFCTYH--IDKLYCDNW : 179
OR125-8   : DS--YVISSHMCALQTYVIYSSLLSDITILTVMSYDRYVAICKPLNYHSKLTKLTCAILILLSWIVPNFSVIPAIMLAHLRPFCKYH--IDKFYCDNW : 179
OR125-3   : DS--YVIPSYMCALQSFVIYSSIKCEYSILAAMAYDRHVAICQPLDYHTKMNPVTCVILLFFCWTVPFITTLISLLLSNKLVICKNH--IDKLYCDNW : 179
OR125-5   : NS--YVISSYMCAIQAFIVYSYVMCECSTLAVMAYDRHVAICQPLDYHAKMNRFSCSILLVLCWLIPFLSMFIAVFLSNRLVLCKNH--IDKLYCDNW : 179
OR125-6   : NS--YVIPSYMCAIQAFIVYSFVMCEFSTLAMMAYDRHVAICQPLDYHTKMNRFSCSILLVLCWLIPFLSMFIAVFLSNRLVLCKNH--IDKLYCDNW : 179
OR125-7   : NS--YVIPSYMCAIQAFIVYSYVMCEFSTLAVMAYDRHVAICQPLDYHAKMNRFSCSILLVLCWLIPFLNMFIAVFLSNKLVPCKNH--IDKLYCDNW : 179
OR125-4   : DS--YVIPSYMCAVQTFVIYSSLLCDCTTVTVMAYDRLVAICQPLNYHNKLNKFSCSVFLSFCWILPFGYVFICVVLSNRKKLCKYH--IKKLYCDNW : 179
OR128-13  : DT--HLISREACGLQSFVIYSYITNENTILMLMALDRFVAISKPLQYHNIITPRLLALLFLLNWAYSMLCIGVIAVFTSGLKMCGNK--LWKVYCQNY : 176
OR128-7   : DA--HSISREACVLQSIVINSYIANENTILMLMALDRFVAISKPLQYHNIITPRFLALLFLLNWAYSMFCFCVVAIFSSGLKMCGNK--LWKVYCHNY : 176
OR128-9   : DT--HLISRGACILQSIVINSYITNENTILMLMALDRFVAIIKPLQYHNIITPRFLALLFLLNWAYSMLCISVIVIFTLELKMCGNK--LWKVYCHNY : 176
OR128-8   : DT--HLISREACGLQSFVIYSYITNENTILMLMALDRFVAISKPLQYHNIITPRLLALLFLLNWAYSMLCIGVIAVFTSGLKMCGNK--LWKVYCQNY : 176
OR128-6   : DT--HSISREACFLQSFVIYTYITNENTILMLMALDRFVAISKPLQYHNIITKRFLTVLYLSNWAYSVLCIGIAAIFTAGLKLCGNK--LWKVFCHNY : 176
OR128-10  : DT--HLVPREACLLQAFVIYSYASNENTILMLMAFDRYVAICKPLQYNSIMTPRNLSVLIALSWIYPMLCVGIAAILNARLTMCGNK--LWKVYCHNW : 176
OR128-1   : ET--HSISREACFFQSFVIFTYAANEFSILMIMAFDRFAAICKPLHYHSIVRPRFLACVIVTNLTFPMILLGVAGLLTTKLRMCGNK--LFKVYCHSY : 176
OR128-2   : ET--HSISREACFFQSFVIFTYAANEFTILMLMAFDRFAAICKPLRYHSIVRPRFLACLIVINLTFPMILLGVSALLTTKLKMCGNK--LFKVYCHSY : 176
OR128-3   : ET--HSISREACFFQCFVIFTYAANEFSILMLMAFDRFAAICKPLRYHSIVRPRFLACLIVTNLTIPMILLAVGSLLTTKLRMCGNK--LTKVYCHTY : 176
OR128-4   : ET--HSISREACFSQSFVIFTYVANEFSILMLMAFDRFAAICKPLRYHSIVRPRFLACLIVINLNCIMILLCVAALLTTKLRMCGNK--LFKVYCHSY : 176
OR128-5   : ET--HSISREACIFQCFVIFTYAANEFSILMLMAFDRYVAICKPLHYHSIVRPRFLACLIVLNLTVPMTLIGVGSLLTTNLKMCGNK--LFKVYCHTY : 176
OR127-1   : DT--NAISRPACFVQIFTIYSYAMSEYTILTLMAYDRYVAICNPLKYHKIMSLRLTARYMAMASFCVVFSMALVIFFSAKLPLCGTD--VTRLYCSNW : 179
OR122-2   : SKT-PEVPLTVCHAQIFALYSYAQVEFCNLAMMSYDRYVAICHPLEYHRIMSPARTMTLIALGWSLEIMILSFSFSVNVQLKLCGNV--LDKVWCDQY : 180
OR122-1   : SKT-PEVPLTVCHAQIFALYTYGQVEFCNLAMMSYDRYVAICHPLEYHRIMSPARTMTLIALGWSLQIIIFSLSFSVNVQLKLCGNV--LDKVWCDQY : 180
OR121-1   : ND--NTISLVYCYLQIFCLYTYAMIEFCNLAVMSFDRYVSICYPLQYNRIMTPVRVGLLIALVWIYCFIQFFIFLSFNFKSQLCGNV--MEKVWCDNY : 180
OR120-1   : DI--KDMLYTDCIIQVFCINVYGTCAYALLTLMAYDRYVSICKPLQYHNIMTPGKIRLLLFIAYFVPICSLGIQVYLTSRLPVCRYT--INKLFCDNL : 179
OR115-14  : EPSERYITFVECVIQAYFAHLNGTASHTVIMIMAFDRYVAICNPLRYPAIMTSNMVVKLSVAAWAVAAVLVGILIGLSVRLSHCRSV--IQGSICDNP : 182
OR115-2   : DPSKCYITFVECAIQAYFTHLNGTASHTVIMIMAFDRYVAICNPLRYPAIMTSNMVVKLSIAAWGVAAVLVGILIGLSVRLSHCRSV--IQGNVCDNA : 182
OR115-13  : DPSERHITFVECVIQAYFAHLNGTASHTVIMIMAFDRYVAICNPLRYPAIMTSNMVVKLSAAAWGVAVVLVGILIGLSVRLSHCRSV--IQNYICDNA : 182
OR115-12  : DPSERYITYIECVLQAFFTHLYGTTSHTILMIMAFDRYVAICNPLRYPTIMNNKMIVKLSAGAWGAAIVLVGILIGLSVRLSHCRSV--IENHVCDNP : 182
OR115-10  : EPSERYITYVECVIQAFFGHLYGTTCHTILMIMAFDRYVAICNPLRYPAVMTSNMVVHLSAAAWGVAVVLVGILIGLTVRLSFCRSV--IENPFCDNA : 182
OR115-11  : NPSERYITYVECALQAFFIHLNGTACHTILMIMAFDRYVAICNPLRYSAIMSNKMVIKLSAGAWISMSVMVGIMISLSVRLSHCRSL--IQNHFCDNA : 182
OR115-9   : EASERYISYTECVVQAYFVHVFTAACHYVLMIMAFDRYVAICNPLRYTAIMTNKMVIKLSASAWGLAIIVVTVMIGLTLRLSHCRST--IENPFCDNA : 182
OR115-7   : EASERYISYAECAIQAYFVHVFTAACHYVLMIMAFDRYVAICNPLRYTAIMTNKMVIGLSALAWGLAIIFVTIMVGLSLRLSRCRYK--IENPFCDNA : 182
OR115-4P  : EASERYITYVECVVQAYFVHIFAAASHYVLMIMAFDRYVAICNPLRYSAIMTNKMVVKLSVSAWGLSVFIVSIMLGLTIRLSRCRYK--IENPFCDNA : 182
```

Figure S1

```
                                    TM3                                       TM4
OR115-1   : DSSERYISYAECVVQAYFVHLFAIACHYVLIIMAFDRYVAICNPLRYTAIMTNKMVVKLSASAWGLSIFMVSILLGLTIRLSRCRSY--IENPFCDNA : 182
OR115-6   : EASERYMTYVECVVQAYFVHIFAAASHYVLLIMAFDRYMAICNPLRYTAIMTNKMVVKLSALAWGWSVISVTILISLTLRLSRCRSN--IENPFCDNA : 182
OR115-8   : EASQRYMTYTECVVQAFFVHVFASVSHSVLIIMAFDRYVAICKPLRYAAIMSNKMVVRLSALAWGVSVIAVTVMIALSLRLSRCRSY--IENPFCDNA : 182
OR115-5   : QTSERSMTYVECVVQAYFVHIFTTASHTALVTMAYDRQVAICNPLLYTAVMTDKMVVKLSATAWTLPIIVVAVMIGLTVRLSRCRSK--IENPFCDNA : 182
OR115-3P  : DTLQRYITYMEGVLKPYFGHILISTCHYVPVIMVFVRLAAFCNPLRYSAIMTNKMVIKLSASAWGLAILLVTIMLGLTIRLSHCRSK--IENPFCDNA : 183
OR116-1   : ET--NNMYYPLCVLQGFLLHMYGGGVMFVLAAMAFDRYIAICMPLRYNALMTPRAVMTVVALVWGLVFLIICVLFSLHGLHSRCRSH--IVSLFCDNP : 181
OR116-2   : ET--NKTYYPLCVLQGFLLHMYGGGVLFVLAAMAFDRYIAICMPLRYNALMTPRAVMGIVALVWGLDLFFIILLFALQSKYSRCRSD--ILNVFCDNP : 181
OR117-1   : ET--NDVYYPTCVLQAFLIHLYGGGTLFLLAAMSLDRYIAICNPLRYNSIMTPSRIFGLIAFAWGLDFAVITVLFSLQARLVKCKSF--ITNVYCDNP : 183
OR118-1   : QD--RSISYSACLLQGFFVHLNGGASYLILTAMAYDRYITICCPLRYGAIMTTNNLLRIIGMWLFNVVAITVICVLLLPYRICQTH--MTDLVCYKT : 181
OR118-2   : QD--TSISYPACLLQGFIVHLYGGASYVILTAMAYDRYIAICCPLRYGAIMNSNNLIKIITGMWVFNFTVITVLFSLLLPYKICRTH--MTDLFCYNP : 181
OR118-3   : QD--RSITYSACLTQGFFIHFYGGASHVILMVMAFDRYIAICLPLRYGAIMTPSNLMRIISVMWFIHFVIIFVLFCLLMPHRICQTY--MADLICYNP : 181
OR119-1   : DT--RTMSFSVCITQAFFIHVYAVSAVFILAAMAYDRYVAICQPMRYTTIMTNIHLAKIISLVWLSNLILMAVLFILLLRLPRCRSF--LNHPYCDNP : 183
OR119-2   : DT--RTMSFSVCITQAFFIHVYAVSTVFILTAMAYDRYVAICQPMRYTTIMTNIHLAKIISLVWLSNLTLMAVLFILLLRLPRCRSF--LNHPYCDNP : 183
OR132-5   : ----LPIPKSICTLLVFISHCTFRNAPLTLALMSLERYVAVCFPLRHCTIATPKRTGIGIGIIWFLSTINIITDIILALILNPNF----ADFIFCTLE : 181
OR132-1   : ----LPIHKSICTLLVLISYCTFFNAPLTLALMSLERYVAICFPLXHCTIAHPKRTGIAIGIIWFLSSVNVVIDIIIALINNPSYL---AEIAFCTLE : 182
OR132-2   : ----YPIHKSICTLLVFTSTLAFINAPLTLALMSLERYVAICFPLRHCTIATPKRTGIAIGIIWFLSTINVIIDIIIALVNNPNYL---ADIAFCTLE : 181
OR132-3   : ----YPIHKSICTLLVFITHCAFRNAPLTLALMSLERYVAICFPLRHCTIATPKRTGIAIGIIWFLSSINVIIDIIIALVDNPYVL---AEIEFCTLQ : 181
OR132-4   : ----YPIQKSICTLLVFITHCAFRNAPLTLALMSLERYVAICFPLRHCTIATPKRTGIAIGIIWFLSSVNVIIDIIIALVNNPYVL---AEVAFCTLE : 181
OR133-1   : ----PFVMKIVCLILLVAATALYQVSTLNLALMSLERYVAICFPLRHAEITTYGRTNMAIGVIWMISWIQSLSEIVIFYSIDTTNI---AVNLFCSRT : 175
OR133-2   : ----IFIFKNICILLLAAAQSLYQVALLNLALMSLERYVAICFPLSHAXXXXXXXXXXXXXXVVWVIGLIQCLSEMIIFYSIDSTNT---VMNLFCSRT : 177
OR133-3   : ----ILIMKNVCLLLLVSATALYQISTLNLSLMSLERYVAICFPLRHVELTSFERTRLAIGVVCIISMIQPLSETIIFYCIDTTNI---AMHLFCSRT : 175
OR133-4   : ----ILIMKNVCLLLLIAATSLYQISTLNLSLMSLERYVAICFPLRHAEFTSFERTHIAIGVICFIGMLQSLSETIIFYCIDTTNI---AMNLFCSRT : 175
OR133-6   : ----ILIMKNVCLLLIIAATSLYQVATLNLSLMSLERYVAICFPLRHAEFTSFERTHIAIGVVCVISMIQPLSETIIFYCIDTTNI---AMHLFCSRT : 175
OR133-5   : ----ILIMKNVCLFLLIAATYLYHISILNLSLMSLERYVAICFPLRHAEFTSSERTHIAIGVICILGMIKSLSEIIIFYCIDTTNI---EMSLFCSRT : 175
OR133-7   : ----ILIMKNVCLLLLIAATALFQVSTLNLALMSLERYVAICFPLRHAEFTSFKRTHTAICVVWVLGLIQCLSEMIIFYSFDNTNI---SMNLFCSRT : 175
OR133-8   : ----ILIFRNICLALVIAGKALFHVSILNLSLMSLERYVAICFPLRHAEITNFERTHTAICVVWVLGLIQCLSEIIIFYSIDPANT---VLNSFCSRI : 175
OR133-9   : ----FLIIRNICLLLLVSAKALFHVSILNLSLMSLERYVAICFPLRHAEFTSFKITHTAIVVVWVLGLIQCLSEVIIFYSFDSANT---VLNSFCTRI : 177
OR137-7   : ----HNVPVPFCVLLLLITSVTHRNTPLTLAGMAIERYIAVCKPLHHHQICTVRWTYMLISLIWSVGILPGLTDIFLLLINGPLAL---FT-TICTSV : 171
OR137-1   : ----NNVPVPFCVTLLIIASTAYKNTPLTLAGMAVERYIAVCKPLHHHQICTVRRTYILISLIWSVGVLPGLAELILLLIIRPLAV---FTRTQCASV : 172
OR137-2   : ----QNVPIPFCVTLLIISSATHKNTPLTLAGMAIERYIAVCKPLHHHQICTVRRTYILISLIWSVGVLPALADLILLSIVRPLTIS--STVTSCSAS : 176
OR137-3   : ----PNVPVPFCVTLLLISSTTHKNTPLTLAGMAIERYIAICKPLHHNQICTVRRTYILISLIWSVGVLPSLADLILLSIVRPLAF---YTSALCSTS : 172
OR137-8   : ----QNVPFPFCVILLIIASKTHKNSLLTLAGMSIERYIAICKPLHHYQICTVRRTYILISLIWSVGVLPGLADFIVLLLVDPSIF---TKTTVCTTA : 172
OR137-4   : ----QNVPFSFCVILLLIVAKTQTNTLLTLGGMSIERYIAICKPLHHHQICTVRRTYILISLIWSVGVLPGLADFILLLLIDPSII---AKATVCTPA : 172
OR137-5   : ----RNVPIPFCVVLLIIASKTHKNSVLTLAGMSVERYIAICKPLHHHQICTVRRTYILISLIWSVGVLPGLADFILLSVIDPFFY---TKDTICSTS : 172
OR136-2P  : ----PLVNASLCCILLVLGTTTFMITPLNLAGMAIERFIAICKPLHHSQICTPQRTYIFICLLWIVGVIPSLADIVILFSIQPMSFF--RSSVPCYPV : 170
OR136-1   : ----PVVNASLCYILMSLGKVFFMITPLNLAGMAIERFIAVCKPLHHSQICTPQRTYVFFCLLWGIAAIRSVVDIMISLLTQPISFF--RSFVPCYPT : 180
OR135-2   : ----PNMNVAICCFFVLIASNVYKNTPLNLAGMAIERFVAICYPLHHAQI-TVRNTKILIGIIXLIGAIPGLLDLFVVLILNPLTFF--NSSCICHQQ : 173
OR135-1   : ----PNLNVAICCVLVLIASNVFKNTPLNLAGMAIERFVAVCYPLHHAQICTVQNTKILIGIIWLMGALPGMLDFLVVLALRPLSFF--TSNRICFLQ : 170
OR140-1P  : RA--PSNGSNCCAPEICFLSILGKNTPLNLACMAIERYSAICKPLHHLQICTVRRTYMLICFIGTASALPALSDIFIAISIRTASFF--STSNLCHSA : 171
OR134-1   : ----YTISVGVCCFFILVAVFTTRSTPVNLAGMAIERYIAICYPLRHAQICTVHRAYVLIGVIWFVSVVPDITDLFVTLATEPLSFF--QTSVFCLRQ : 174
OR129-1   : ----VQFAIIYCAPLLFFSTATFLNTPLILSTMSLERYVAIFYPLRRPAMWRSEWIWTIIVSLWIISCILPTVDFILMRLKPNVNIL--STPVLCKTT : 176
OR131-1   : ----LIIHNISCYVFCTVMSVLSICSPVTLVAMCLERYVAICLPLRHASISSPKNIINGLLIVWGVSSVIPLFVFIGSFAYTPPNAM--NSYVVCSIE : 173
OR131-2   : ----LFIHIISCYIFCTVMALLSICSPVTLVAMCLERYVAICLPLRHASISSPKNTINGLLIIWGVSSVIPLFIFIVSFTYTPPNAM--NSYVVCSND : 173
OR130-1   : ----VLLPLQFCIPVCMFIETVAVSAPLTVGAMCVERYVAICLPLRHHAISTSRRAFMVILMIWIVSSINPFIDMFILVSTASHEYL--TQLTHCHYE : 178
```

Figure S1

```
                                   TM5                                                  TM6
                       ___________________________                          ___________________      _
              200         *         220         *         240         *         260         *         280         *
OR106-12  : SMIRAAC-VD-PEP-YFFIPAMLNMWLLCGQFPFILCTYAVLAYSVTKLAN--NSSKKQMINTCLSHIIVLSSFYAPKLVSSLLTRI-GVV-----LT : 269
OR106-11  : SMIRAAC-VD-PEP-YFFISAMLNMWLLCGQFPFILCTYAVLAYSVTKLAN--NSNKKQLINTCLSHIIVLSSFYAPKLVSTLLTRI-GVV-----LT : 268
OR106-1   : SMIRAAC-VN-PEP-YFFLPAMINLWWFCGLFPFVMCTYAVLAYSVTKLSN--NSSRKQMINTCLSHLVVLLSYYAPKIVSALLTRI-GVV-----LT : 267
OR106-2   : SMIRAAC-VN-PEP-YFFLPSMINLWWFCGLFPFVMCTYAVLAYSVTKLS---NSSRKQMINTCLSHLIVLLCFYAPKLVSNVLTRI-GVV-----LT : 268
OR106-3   : SMIRAAC-VN-PEP-YFFLPAMINLWFFCGLLPFVICTYAVLAYSVTKLS---NSSRKQMINTCLSHLIVLFSFYAPKLVSYLLTRI-GVV-----LT : 268
OR106-4   : AVVRAAC-VD-PEP-YFAISTIYSMVPLCGQFPFILCTYAVLAYSVTKLS---NSSRKQMINTCLSHLIVLLCCYAPKVVSYLLTRI-GVV-----LT : 268
OR106-6   : AVVRAAC-VD-PEP-YFAITTIYSMVPLCGQFPFILCTYAVLAYSVTKLS---NSSRKQMINTCLSHIIVLLSFYVPKIVYILLTRI-GVV-----LT : 268
OR106-8   : SLVRAAC-VD-PEP-YFAISTMYSMVTLCGLFPFILCTYAVLAYSVTKLA---NSSRKQMINTCLSHIIVLFSFYVPKIVYILLTRI-GLV-----LT : 268
OR106-9   : SLVRAAC-VD-PEP-YFAISTVYSMVTLCGPFPFILCTYAVLAYSVTKLA---NSSRKQMINTCLSHIIVLFSFYLPKIVYILLTKI-GVV-----LT : 267
OR106-10  : SLVRAAC-VD-PEP-YFAISTVYSMVTLCGLFPFILCTYAVLAYSVTKLA---NSSRKQMINTCLSHIIVLFSFYLPKIVYILLTRI-GLV-----LT : 267
OR106-7   : ALVRFSC-VD-PEP-YFAISTIFSMWPLCGQFPFILCTYAFLAYSVTKLS---NSSRKQMINTCLSHIIVLLSYFAPKIVYILLTRI-GVV-----LT : 268
OR106-5   : SLVRAAC-VD-PEP-YFVISTIYSMVPLCGLFPFVMCTYAFLAYSVTKLS---NSSRKQMINTCLSHLIVLLSYYAPKVVTILLTRI-GVV-----LT : 267
OR108-1   : PVLRVAC-SN-TTS-QWNFATILSILFLAVPLCFIFLTYMFILYAVFRMNN--NQRRFKALATCTEHLILVSLYFFPIAIIFSLGFF-GVI-----IN : 270
OR108-2   : PVLRVAC-SD-TAS-QFNFGTASNILFVIIPFAFIFLTYAGILYAVFRMKN--NQSRFKALATCTEHLILVALFFFPIVIIFSLGLF-GYI-----IN : 275
OR108-3   : PVLVLAC-SD-ITQ-QLNYATAATILFIGVASIFIFFTYIGILRAVFRMKS--SQSRFKALATCTEHLVLVCLYFVPIVIIFNLTFF-GII-----WS : 274
OR110-1   : PIYRSAC-NN-NFP-SSVIASLFPAVILGFPAFFIIFSYTCIAVTLFRITT--PQDRQRATKTCMAHLILVAVFYVPITFTYAFWSF---------ID : 271
OR110-2   : PIYRSAC-SN-NFP-SSVIGSLYPIVILGLPVFFIIFSYTCIAVTLFRVTT--PQDRQRATKTCTAHLILVAVFYLPITITYSLASI---------IN : 270
OR109-1   : PICGLAC-ND-ITI-NILIGKTSYGLLICTPLILIFISYFCISVALLRIAH--GAERIKALKTCTSHFMLVAMYYLPVLTNIIASAT--TP-----LH : 271
OR109-2   : LLNKLAC-ND-NSI-NILMIKICFGVMIGMPLMLIFVSYFCISLALFKIAH--GADQAKAIKTCTSHLMLVAISYIPYLSSHIAFLT--SH-----IQ : 275
OR109-4   : LINRLAC-ND-NSV-NIMMVKINFGLLICTPLILIFISYFCISLALFKIAH--GVDRAKAIKTCTSHLMLVAIFFIPFLSNQIAILT--TS-----VH : 275
OR109-3   : LINRLAC-ND-NSL-NILMIKINFGLLLCAPLILIFVSYFCISLALFKIAH--GADRVKAIKTCTSHLMLVVVGYTPLISNQIAVLT--AP-----MP : 275
OR109-5   : LIYKLAC-ND-ISI-NLLMGKMTFGLLMCTPLILIFLSYFFIALALLKIAH--GGARIKAMKTCTSHLMLVAIGYFPLISNQIAALT--TS-----ID : 272
OR109-6   : IIYKLAC-ND-NSI-NLMMGKISFGLLMCTPFMLIVISYFFIALALLKISH--GVDRIKAMKTCTSHLMLVAIGYLPLISNQIAALT--TS-----ID : 275
OR109-9   : IIYKLAC-ND-NSI-NLMMGKISFGLLMCTPFMLIVISYFFIALALLKISH--GVDRIKAMKTCTSHLMLVAIGYLPLISNQIAALT--TS-----ID : 275
OR109-7   : TVYRLAC-ND-NSI-NILMGKLGNNLLVFTPLILVFISYFCISLALFKIAH--AVDRTKAIKTCTTHLMLVAIFYIPILSNNIAALT--TP-----FT : 272
OR109-10  : TVYRLAC-ND-NSI-NILMGKLGNDLLVFTPLILVFISYFCISLALFKIAH--AVDRTKAIKTCTTHLMLVAIFYIPILSNNIAALT--TP-----FT : 272
OR109-8   : PVFRLAC-ND-NSM-PILMGKLCFGILICMPPILIFVSYFCISLALLKIAR--GVDRNKAIKTCTSHLMLVAIFYLPILSNNIAYAT--TY-----IN : 272
OR109-11  : PVFRLAC-ND-NSL-PILMGKLCFGILICMPPILIFVSYFCISLALLKIAR--GVDRNKAIKTCTSHLMLVAIFYLPILSNNIAYAT--TY-----IN : 275
OR111-1   : PVYRLAC-SD-ISK-NKIMAYVISAMYIIAPMVVIVFSYLGIFLALIKITT--WEGRLKALKTCVSHLLLVGIFFLPLFCTYLAQLL--LS-----LN : 275
OR111-2   : PVFRLAC-ND-NSM-NKIMAYVISVLYILAPMVVIVFSYLAIFLALIKITT--WEGRLKALKTCVSHLLLVGIFFLPVCCTYIAQVL--LY-----LT : 275
OR111-3   : PIFRLAC-ND-IKI-NEFFAFFISILYLTMPMVIIALSYLNIFLALIKITT--WEGRLKALKTCVSHLLLVGIFFLPLLCTYIAQVL--LA-----LT : 275
OR111-4   : PLFRLAC-ND-ARF-SEFFAYFISALYITMPLVIIVLSYLHIFVALIKITT--WEGRLKALKTCVSHLLLVGIFFLPIYCTYIAQVL--IP-----LT : 275
OR111-5   : PVYRLAC-ND-FNL-NKSVGFVITSLFLIAPMIIIFLSYLVIFLALSKITT--WERRLKALKTCVSHLLLVGVYFFPICFTYLAQLL--LA-----LT : 275
OR111-10  : PVYRLAC-ND-NSI-NRFMGSFITCLYLVVPLAIIILSYIGIFLALNKITT--WESRLKALKTCVSHLLLVGIYFLPMSCTYIAAWL--LA-----LA : 275
OR111-7   : PMFRMAC-ND-NNI-NKIMGFLYTTLYLIAPMLVIFLSYLGIFLVVSKIAT--WERRLKALKTCVSHLLLVGIYFLPIFFTYLTSLL--LF-----ST : 275
OR111-6   : PVYRIAC-ND-FSI-NKFMAFLITGLYLVIPLFLIVLSYLGIFLALTKITT--FEGRIKALKTCVSHLLLVGTLFLPLFCTYFAQLL--LS-----LS : 275
OR111-9   : PVLRLAC-ND-STV-NKIITNVIAVMSLVAPVILIVLSYLGIFFALSKITT--WEGRLKALKTCVSHLLIVGLFFLPVICIFIASST--ST-----LT : 270
OR111-11  : PVYKLAC-ND-NSI-NSFLGKLSTALYLVAPLIIIFLSYVGVFLALSKITT--WEGRLKALKTCVAHLLLAGSFFVPIFSIYIASIM--NS-----LT : 275
OR111-8   : PVYRLAC-ND-NSI-CASMAQLSAALYVVAPVIIIVLSYLGIFLALSKITT--WEGRLKALKTCVSHLLLVGSFFLPIICIFIATST--NS-----LS : 270
OR107-1   : PVYRLAC-TD-TTY-NRQLGTVKTMITLLGPLFFIVFTYVIVVIAVMRIAS--VTQRWKAFHTCLTHMMLVMLYYMPVIIAYVLGNL-RLV-----QN : 274
OR104-1   : SMNQLAC-AD-ASF-YNLIAFGVAMFVLLVPLSFIIFSYASILVSVLQIAN--AKGRLKALSTCATQLTIISIYYVPRFAVYITSNVPNAQ-----MD : 274
OR104-2   : SINQLAC-AD-ASN-ANFIAFCLIMFVLLVPFAFIIFSYACILVSVLQIAN--AKGRLKAFSTCATQLTIISIFFVPRFVVFSSNNL---------PS : 271
OR105-1   : SITNLAC-AE-NSK-QLLVALCMALLVLLLPLVCIIYSYCHIIASVMRLSS--SQSRWKSFATCSTQLCIITLFYMPRFTVYLTTFL-QIQ-----IS : 270
OR103-1   : SITSLAC-TN-RAP-YSIPAFVLAMVALLTPLAFIVFSYCAIILAVLRISS--TQARLKTFSTCSPQLIIIALYFLPRCFIYLSSNI-GIY-----FS : 270
OR103-5   : SITTLAC-TD-RSL-YSIPALIYAFLILLVPFAVIIFSYCSIFIAVMRISG--SQGRMKTFSTCSPQLIIIALYFIPRILNFLSSNI-GLK-----FS : 270
OR103-2   : SITTLAC-TD-RSL-YSIPALIYAFLILLVPFAVIIFSYCSIFIAVMRISG--SQGRMKTFSTCSPQLIIIALYFIPRVFNYLSSNI-GLK-----FS : 270
OR103-3   : SVTTLAC-TN-RSL-YSIPALIYALAVMAWSIAIIAFSYCSIFKAVMRISG--SQGRMKTFSTCSPQLIIIALHFLPKGLIYLFGNI-GIK-----FD : 270
OR103-4   : SVTTLAC-TD-RSL-YSIPALIYALVLLMGSFAIIVFSYCSIFIAVMRISG--SQGRMKTFSTCSPHLMIIALYFIPKCFLYLFGNI-GVK-----FS : 268
OR102-1   : SIIKLSC-GD-IGQ-KKFYSFSFAMFVLLGPLIFIIFSFTAIIISVFRISD--TQARFKTFSTCTPQLLIICLYYVPRCVVYVHDVT--IA-----IS : 268
OR102-3   : VVIKLSC-RD-IRQ-KLYLTFACTVIIVVGPPIFILCSFIGIFTSVFNISN--SQARYKTFSTCFPQLFLICLHFLSKLVVYVYDVT--VT-----MS : 278
OR102-4   : VVIKLSC-RD-VRQ-KLLLTLAWALIVVLGPSLFVLFSFIAIFMFVFNISD--NQTRYKTLSTCTPQLFIICLYFVPKIVLYVYDIT--VT-----MS : 278
OR102-5   : VVIKLSC-RD-VRQ-KLLLTLAWAMIVVLGPSLFVLFSFIAIFMFVFNISD--NQTRYKTLSTCTPQLFIICLYFVPKFVLYVYDIT--VA-----LS : 269
OR102-2   : SISKLAC-GD-AAT-MKVTSTAIAMFVLWGPLSFILFSYVSIIISVSKISK--SEGRYKTFSTCTPQLLIICLYYLPRTFVYITNIS-GYE-----LS : 277
OR101-1   : SVLTLAC-VD-TTR-NSFFALALALIGLLLTFLLILISYVYIGKAMRRMS---MAQKLKAGATCVSHLIVVFISYCCAFFVYISYRV--SK-----FD : 260
OR113-1   : ALVQLAC-GD-ISA-NNIVGLLSAFLIAATDCILITVSYIVMFFYVLKS----GTANMKALNTCVTHLIVLSISLTSALTAFLSYRI-RNN-----LS : 269
OR113-4   : ALVQLAC-GD-TSV-NNIIGLVSAFHIPTADFIVITLSYVVIFTSVLKS----GKAHAKALNTCITHLIVLTFSLTSALTAFLSYRI-KNN-----IS : 269
OR113-2   : ALVQIAC-GD-TSV-NNIVGLLCAFLITTVDFILITVSYVVIFFSVFRS----GKAHLKAINTCVTHIIAMTLSLISALTAFLLYRI-RNN-----IS : 269
OR113-3   : ALVQLAC-GD-ISI-NNIVGLLTVFFVPATDLILITASYVVMFTSVFKS----GETRMKALHTCITHLIVLSVALTSAMIAFLSYRI-RND-----IS : 269
OR114-1   : SVVSIAC-GD-VSK-NYAAGTV-AFSIVTGDCVFIMCSYIVIFVIIFRSPP--GDSRQKAINTCSTHLMVMVVAYCSCVVAYVGYRV--SS-----IP : 268
OR112-1   : ALVSLAC-DS-IAQ-NQTMGLIVIICFVGIDTSVIFFSYVKILHVVLGTAA--GEDRWKAFHTCGTHLMVMICFYFVGSVTFLSRNL-NIP-----IP : 272
OR124-3   : SLMKLAC-KQ-DFF-QGIFNGFLYISMSVL-LVFVLFSYLKIILACRKS----KVSREKFYSTCMPHLITFLNTS-CGISDSLCTHFNVET-----LS : 264
OR124-2   : SLLNLAC-KQ-DFF-QGTFNGFVYISMGVF-FVFVLFSYFKIILACRKS----KVSREKFYSTCIPHLISFLNTT-CGLSDSVCTQFKVET-----LS : 264
OR124-1   : SLLNLAC-KH-DIF-EGIVNGFIFIIMSFL-FVFVLLSYLKIILACRKS----KVSREKFFSTCIPHLISFLNTT-CGLSDSLYTKFNIDT-----LS : 264
OR124-4   : SLVNLGC-KP-DIS-YGIFNGWMYGSVIIL-IGFVLISYFKIILACRKS----KVSREKFYSTCMPHLIIFLNYIGCSFFDSSQTELRAKSQY---VS : 267
OR123-1   : VLERLVC-GV-DTA-QFVFNGILTCAVNAL-VWFVFLSYINILVACKHS----VQNHKKFISTCLPHLIAFLNYVFWSLFSTLYELFGISS-----LS : 264
OR126-1   : SVVKLSC-FS-TTV-NNVVGYVIIIVYFGH-AVLIFCSYIYLVVKCRKS----TESRHKFIQTCVPHLLALLNVTVALLFDVLYSRYGSKS-----LP : 268
OR126-2   : SVVKLSC-FS-TTV-NNVIGFVIIIIYFTH-AVLVFCSYMYLIRKCRKS----IEGRHKFIQTCVPHLLSLINVTTAFLFDVLFSRYGSKS-----VP : 268
OR126-3   : GVAKLSC-FS-TTL-NNVFGYIVIIAYVGH-AILIFCSYIYLIRKCQKS----IESRHKFMQTCVPHMISLLNVTVAFLFDVLFSRYGSKN-----VS : 268
OR126-5   : AVAKLSC-FS-TTI-NNVFGYIVILTYFGH-AVLIFCSYIHLIKKCMKS----IEVRHKFIQTCVPHLLSLINVTAALLFDVLYSRYGSRN-----LP : 268
OR126-4   : AVAKLSC-VS-TTV-NNVFGFIVILTYFGH-AVLVFCSYIYLMRKCRKS----IEDKNKFIQTCVPHILALMNVTIAMLFDVLFTRYGTKN-----VP : 268
OR125-1   : SIVKLSC-VS-SFV-NNVFGYGIAVIFFSF-VVLIIFSYIKLVAACKAS----LENRKKFWQTCLPHIFSLINFTFAFLFDFMYNRYGSKD-----IP : 264
OR125-2   : SVVKLSC-AS-SFV-NDVYGYIIGVVSISF-AVFIIFSYIKLVAACKAS----LENRKKFWQTCLPHIFSLINFCIAITFDFMYSRYGAKD-----VP : 264
OR125-8   : SVVKLSC-AS-SFV-NNVYGYIVTVIFISC-AVIIVVSYIKLIAACKES----LENRKKFWQTCLPHLFSLMNLIFTLLFDVTYSRYGSND-----IP : 264
OR125-3   : SMVKLSC-ES-TDT-NNIYGLISISFHFGI-FIVIIFSYVKLVTACKSS----LECRKKFWQTCMPHVVSLVNFSVAVLFDTMYSRYGSSD-----FP : 264
OR125-5   : SIVKLSC-GS-TVI-NNIYGFVFISLYVVL-VIVIIVSYIKLIIACKSS----LECRRKFLQTCVPHVISLLNLTVAALFDNFSNRFGSDD-----VP : 264
OR125-6   : SIVKLSC-ES-TVI-NNIYGFVFISFYFVL-VMVIVVSYIKLIIACKSS----LECRRKFLQTCVPHVISLINLTVAALFDNLSNRFGSDD-----VP : 264
OR125-7   : SIVKLSC-ES-TVI-NNIYGFVFISLYFVL-VIVIIVSYIKLIIACKSS----LECRRKFLQTCVPHVTSLINLTVAILFDTLCNRFGSDD-----VP : 264
OR125-4   : SIVKLSC-ES-HDM-NSILGLFVIAFYSCL-CVFIFFSYVKLIIACKAS----LECRRKFWQTCVPHMFTVINYMFSAFFDAMYGRYGAGD-----IS : 264
OR128-13  : EVVKLSC-VN-SII-INIMGLYLLITTVIIPLSLIIYSYVKILIICQRSS---SQFMRKSFQTCVPHIVVLLNFSVSVVSEITLSRVVNLN-----MS : 263
OR128-7   : EVVKLSC-VN-NII-INIMGLYVLATTVIIPLSLIIYSYVKILIICRRSS---SQFMRKSFQTCVPHIVVLLNFSVSVVSEITLSRVVNLN-----MS : 263
OR128-9   : EVVKLSC-VN-SII-VNIMGLYMLITTVIIPLSLIIYSYVKILIICQRSS---SQFMRKSFQTCVPHIVVLLNFSVSVVSEITLSRVVNLN-----MS : 263
OR128-8   : EVVKLSC-VN-SII-INIMGLYLLITTVIIPLSLIIYSYVKILIICRRSS---SQFTRKSFQTCVPHIVVLINVSVSVISEITLSRVVNLN-----MS : 263
OR128-6   : EIAKLSC-VN-SLI-VNIIGLYVLATTVIIPLSLIIYSYVKILIICRRSS---PQFKKKSFQTCVPHIVVLLNFSVSVISEITLSRVVNLN-----IS : 263
OR128-10  : EIVKLSC-AS-TII-NNVFGFFIVTTTVIMPLSFILYSYVKILIICRKSS---PEFRRKAYQTCIPHIVILLNFSAALFCEVTLSRVQNLE-----LP : 263
OR128-1   : EIVKLSC-DN-IAI-NNAFGLFILIITTIIPLSLISFSYVKIIIICQRSS---AQFKGKAFQTCIPHIVVLLNFTIAVICDTTLSRVVNLQ-----IP : 263
OR128-2   : EVVKLSC-DN-IAI-NNAFGLFIVIITTIIPISSILFSYVKILIICQRSS---AQLKGKAYQTCIPHIVVLLNFTIAVTCDVTLSRVANLQ-----IP : 263
OR128-3   : EVVKLSC-DN-IAI-NNAFGLFILIITTIIPLGLISFSYVKILIICQRSS---AQFKGKAFQTCIPHIVVLLNFTMAVTCDVTLSRVANLQ-----IP : 263
OR128-4   : EVLKLSC-DN-ILI-NNAFGLLLVIITTIIPLGLILLSYVKILIICQRSS---AQFKGKAYQTCIPHIVILLNFTIAVTCDTTLSRVVNLQ-----IP : 263
OR128-5   : EVVKLSC-DN-IVI-NNAFGLFTLITTSVIPIISILFSYVKILIICQRSS---AQFKGKAFQTCIPHIVILLNFTIAVTCDITLSRVVNLQ-----IP : 263
OR127-1   : SVVRLSC-GT-STLSNNILGFFVTTATVFLPAFFILYTYIRILIICQKSS---KEFKGKALQTCLPHIVSFVNYSIASFCDIALSRNDSDK-----IK : 267
OR122-2   : MLVKLAC-SD-TTP-NNIIGITGIPLAIGPPLLIIFFSYAKILRVCLKFT---RQKAKKSLYTCSLHLITLLNFTVGCSFELIHSRFGKLP-----IP : 267
OR122-1   : MLVKLAC-SD-TTP-NNIAGMTGIAVTIGAPLLLILFSYAKILRVCLKFK---GQTAQKSLYTCILHLISLLNFAVGCSFELIQSRFGKLP-----MP : 267
OR121-1   : LLVKLAC-SK-SSL-NNIYGIYLAVVTAVIPLALIFYSYTRIIKICLSFS---KEAKKKALSTCTPHIISLLNFTLGWLFEILQSRFDDIK-----MP : 267
OR120-1   : AVVNLSC-AK-NPW-ADLYGICLVILFVVLPLIFVVFSYVQILFVSLKAS---VSAQRKALKTCTPHLITFINFSLASLFSVIYNRF-NFS-----LS : 265
OR115-14  : SLFKLSC-EN-TVV-NNVYGLTFTVVLFTSSVVCVALTYLRIALICFKSKN--KATNSKAIKTCSTHLTVYIIMLISGSVTILLHRF---P-----VY : 267
OR115-2   : SLFKLSC-EN-TVV-NNVYGLTFTVVLFTSSIVCVALTYLKIAMVCFKSKN--KATNSKAVKTCSTHLTVYIIMLISGSVTIFLHRF---V-----TY : 267
OR115-13  : SLFKLSC-EN-TVI-NNVYGLTFTVVLFTSSMGSIVLTYLKIALICFKSKN--KATNSKAIKTCSTHLTVYIIMLISGSTAISLHRF---P-----VY : 267
OR115-12  : SLFKLSC-EN-TFI-NNVYGLTFTVVLLTSSLGWVVLTYLRIAMVCIKSKH--KATNSKAIKTCTTHLTVYIITIIFGFVPIFLHRF---S-----EY : 267
OR115-10  : SLFKLSC-ES-TAI-NNIYGLSFTVVLLTSSLGSIALTYLRIAIVCFKSKN--KATNSKAIKTCSTHLAVYLIMMVSGLTTITLHRF---P-----EL : 267
OR115-11  : SLFKLSC-ES-TEI-NNIYGLTFTVSVIVVSMGSISLTYLRIAIVCISSKN--K-RNKKAITTCSTHLIVYTIMLVSGFVAVFLHRF---S-----EY : 266
OR115-9   : SLFKLSC-EN-VSI-NNAFGVVYSVIVACLSAISIFITYVKIATVCLASKN--KALNSKAMKTCSTHLAVYLIMFVSGAIFIFLHRF---P-----EY : 267
OR115-7   : SLFKLSC-EN-VSV-NNVFGLVYTVIVLSLSALSIFVTYVKIATVCVTSKN--KALNSKAIKTCSTHLAVYLIMFVTGAVFIFLHRF---P-----EY : 267
OR115-4P  : SLFKLSC-EN-VSI-NNVFGLIYTVIVFTFSLGSVFITYTKIAAVCITSKN--KALNSKAIKTCGTHIAVYIILFASCATMVFLHRF---P-----EY : 267
```

Figure S1

```
                              TM5                                          TM6               _

OR115-1   : SLFKLSC-ES-VII-NNIFGMVYSVIVFTLSLGSLFVTYGKIASVCITSKN--KSLNSKAIKTCSTHIAVYLIMFISCASFIFLHRV---P-----EY : 267
OR115-6   : SLFKLSC-ED-VVV-NNVYGICTTILVSVFSLGSVFITYAKIATVCITSKN--KSFNRKAIQTCGTHIAVYLIMFVSSVIMVFLHRS--PP-----QY : 268
OR115-8   : SLFKLSC-EEGVVI-NNLFGIFSTVVMFTFSLGSVFVTYGKIAASFRNRKH--NSFNSKAMKTCGTHIALYLIMFVSCAIMIFLHRS---P-----QH : 268
OR115-5   : SLYKLSC-EN-VAI-NNIFGIVYSVAALIISVALMVIAYARIVTACIISKS--KALNSKAIKTCSSHIAVYLIMFVSCAIMIFFHRF---P-----EY : 267
OR115-3P  : SLFKLSC-ES-VVI-NNIYGIVYTVAVLCFSAVSIFITYVKIAAVCKTSKS--KALNSKAVKTCSTHLAVYLIMFFSGATMTFLHRF---P-----EY : 268
OR116-1   : SLLKLAC-GD-TTI-NNIYGLFLTAIMQIITISIQAFSYVKILITCLTTRR--SDAKSKAINTCVAQLVIFITFEIVVTFYLLSHRF--SN-----VN : 267
OR116-2   : SLLKLTC-SN-TIV-SNIIGLFNTAMIQIISVSIQAFSYVKILITCLATSK--SDAKNKAINTCVSQLVIFIMFEIVATFTILSHRF--TS-----VN : 267
OR117-1   : SLVLLSCGGD-LSV-NNIYGLFLTAFIQITSVFIQMFSYGCIVITCVKQTH--ADSRVKALNTCVAQFTTFILFEMVTAVAIISYRI--PD-----FP : 270
OR118-1   : PLMKLLC-DG-IEV-NNIYGLCCIIFYHGLALSVVAFTYIHILITCVTNKQ--SDARVKALQTCGTHLVVFLLLEINTLFPLLAHRS--EI-----IP : 267
OR118-2   : SIMKLMC-ED-TTV-NNIIGLISLVLYHCIALSVVAFTYIHILITCVTNKQ--SDAKMKALQTCGTHLVVFLFLEFNTLFPLVAHRS--ES-----VP : 267
OR118-3   : SLMKIMC-ED-TTV-NNMYGLFILSLYHSLPLSVVAFTYIHILVTCVTNRQ--SDAKMKALQTCGTHLVVFLFLEFNTLFPLIAHRA--ES-----IP : 267
OR119-1   : SLLQLVC-AD-TTI-NNIYGLLMTAVSQVCSLGLIMYSYLRILIACFRNKR--SDTRSKALQTCGTHLVVFGLFECLGLFTIISYRI--KD-----IS : 269
OR119-2   : SLLQLVC-AD-TTI-NNIYGLLMTAVCQAFTVGLIIYSYLHILIACFRNKR--SDTRSKALQTCGTHLVVFVLFECLGLFTIISYRI--KD-----IS : 269
OR132-5   : KLFVFKW--------QIDKNQWFDVIYFASVALIIIFTYIMIMITARSVSSD-KDSAKKALKTVMLHLIQLGLCLTSFLYVTIERTLYMMTGSDSSLF : 270
OR132-1   : KLFIAKW--------QIDKSQGFDVLYFVSVAVTIIFTYIMIMITARSVSAD-KESAKKALKTVMLHLIQLGLCLTSFLYATIERTLYMVIGSGSPLF : 271
OR132-2   : QLYIGKW--------QVDKAQVFSVFYFVSVALIIIFTYISIMITARSVSSD-KESAKKALKTVMLHLIQLGLCLTSFLYATIERTLFIMIGSGSPLF : 270
OR132-3   : KLYIAKW--------QVDKAQWFDVLYFVSVAVIIIFTYIMIMITARSVSSD-KDSAKKALKTVMLHLIQLGLCLTSFLYATIELILYMVIGSTSPLF : 270
OR132-4   : KLYIAKW--------QVDKAQWFDVLYFVSVAVIIVFTYIMIMITARSVSSD-KDSAKKALKTVMLHLIQLGLCLTSFLFATIEIIIYKMIGGTSPLF : 270
OR133-1   : TLFRLQI--------YKNLDVAFTCVYFVSVCFIIIFTYASIAAVAKTASGD-KASAKKATKTVLLHLIQLGLCAASILIGVIQEVIYVFTSYA--TI : 262
OR133-2   : TLFRLQI--------YKKLEIAFTCIFFVTVCFIIIFTYASIAAVAKTASGD-KASAKKANKTVLLHLIQLGLCAASILVGVIQEVIYVYTDYM--TS : 264
OR133-3   : TLFRLQI--------YNKLDIAFTSMLFVLVSVVIIFTYASITAVAKTAACD-KMSAKKANKTVLLHLLQLGFGVTSILFGVVQEVIYVYADFV--TA : 262
OR133-4   : TLLRLQI--------YNKLDIAFTSMYFALASFVIIFTYASISAVAKTAACD-KMSAQKANKTVLLHLIQLGLGASSILAGVIQEVIYVNTDLM--TA : 262
OR133-6   : TLLRLQI--------YNKLDIAFTCMLFVVVWFIIIFTYASISAVAKTAACD-KLSAKKANKTVLLHLLQLGLGASSILAGVIQEVIYVNTDLM--TA : 262
OR133-5   : TLFRLQI--------YNKLDIAFTSMFFTLVSFVIIFTYASIAAVAKTAACD-KMSAKKANKTVLLHLIQLGLCALSILAGVIQEVIYVNTDLM--TA : 262
OR133-7   : TLFRLQI--------YKKFDIAFTCIFFVLVSFVIIFTYASISAVAKTAACD-KLSAKKANKTVLLHLIQLGLGASSILVGVIQEAIYVYTDFL--TA : 262
OR133-8   : ALFKLQI--------YKSIDIAFTCIFFVLVSFVIIFTYASIATVAKTAACD-KLSAKKANKTVLLHLIQLGLGAMSILVGVVQEAIYISIDYI--TA : 262
OR133-9   : ILFRLQI--------YKSIDIAFTCIFFVLVSFVIIFTYASIAAVAKTAACD-KLSAKKANKTVLLHLIQLGLGAMSILVGVVQEAIYLSTNYL--TA : 264
OR137-7   : ILYNTPS--------HAELSKISNGIYSSIVWLILVYTYCQVMVKAKRSSTN-KSSAKKAQSTILLHEXQLLLCMLSYITPVLDKIYAPLQT-E--QQ : 257
OR137-1   : TVYSTPY--------HVELTTFMNGIYSSIVWLILVYTYCQVLVKAKRASTN-KSSAKKAQNTILLHGAQLLLCMLSYITPVLDRLYSPLVP-E--QQ : 258
OR137-2   : ILYITPY--------HEVQNKCMNGLYSSVVCLILVYTYCQVLITAKKASTN-KSSAKKAQSTILLHGAQLLLCMLSYISAVVDKIFVPLSP-I--DR : 262
OR137-3   : NLYSTPY--------HEEQSKITHGIYTTVVWVILVYTYCRVLIAARKATAD-KSSAKKAQNTILLHGVQLLLSMMSNITPVIDKIYAQLLP-T--LR : 258
OR137-8   : KLYYTPY--------HEELSKYMTGIYTCVVCLILIYTYCRVLITARKASTD-KSSAKKAQSTILLHGAQLLLCMLSYITGSIEVMYAQLLP-A--DR : 258
OR137-4   : KLYNTPY--------HEELKKYMSSIYASAVLLILIYTYCRVLITARKASTD-KSSAKKAQSTILLHGAQLLLCMLSYITGSIEAMYIQLSP-A--DW : 258
OR137-5   : RLYNTPY--------HEELSKYMTGIYSSGVWLILIYTYCRVLITARKASTD-KSSAKKAQSTILLHGAQLLLCMLSYITGYLELMYAQLLP-A--DR : 258
OR136-2P  : SLFPSKA--------HKDRTTASQVIYMSVVWIILFHTYCRVLFTAKRAIS--KGSVNKARSTILLHGVQLLLCMLSYIAPVLDMILYSFFSCF--TV : 256
OR136-1   : YMFPSKA--------HDDHNTASQVICMSMVWIIICYTYCRVLFAARKAVS--KGSASKARSTILLHGVQLLLCMLSYVTPFMYTLIIPLFP-L--QR : 265
OR135-2   : SVFSFEY--------NIISSAVFNIGYMCSVWVLLFYTYFKVLLSAKAAASE-PGQAQKAMRTILLHGFQLLLCTLSLLTTFLDGVLATLFP-Y--NQ : 259
OR135-1   : NVFDFEY--------HVISHAVFNIVYMCSVWVLLFYTYFKVLFSAKAAASE-PAQAQKARRTILLHGVQLLLCTLSLLTTVLDGALTSRFP-D--YR : 256
OR140-1P  : FVYNSWE--------HSIKETVVNVVYLSLVWISLIITYFNVLSAANAASAD-RTSAKKGRNTIFLHAVQLLLCMMSYVTQVVSSALVERFH-E--SR : 257
OR134-1   : NVFKDPV--------LLYKRQAFDGIYFSLVFLTLLCTYLKIMFTAHSHSTE-NTSAKRATNTILLHGLQLIMCLLSYISPSVEGILNIIFPG---RV : 260
OR129-1   : ILNASPI--------QALFKVSLNGIFFAVVAVVILFTYIRILLETRRMRQD-RTTVRKALHTVLLHGLQLLLSMMAFSQPIAELLLVQRVSL---SQ : 262
OR131-1   : VMLQVKW--------LADTRATSQQLLFVIMLCIVGSTYIKIMIAARSASATNKKSTYKGLRTVILHGIQLILGMMQFITPYTEMSLWKIDVM---LF : 260
OR131-2   : VMFQVKW--------LAEMRALSQQLLFVIMLCIVGSTYIKIMVAAKSASAENKKSTYKGLRTVILHGLQLILGMMQLITPYIDILTLKVDIM---LF : 260
OR130-1   : IMIPEKI--------RVFARGLLYIVGLVVILIIEIFCYVMIYLAARAASGDNKKSASKGQRTISLHILQLFLCTAEVMCPYIESVVMQYDIQ---SY : 265
```

Figure S1

```
                      TM7           ⇓
              300         *         320         *         340         *         360         *
OR106-12 : LTERNAILITASLVSPLINPTVYCTRTKEIRKRLANVFLHRKTVPNPLKSISKGSSS-------------------- : 326
OR106-11 : LTERNAILITASLVPPLINPTVYCIRTKEIRKRLSNVFLHRKTVPNP------------------------------ : 315
OR106-1  : LTERNAILIISSLIPPLINPTVYCTRTKEIRKRLADVFLHRKVAPNNLKVLA------------------------- : 319
OR106-2  : LTDRNAILIISSLLPALINPVVYCTRTKEIRKRLADVFLHKKIVPGHFKVIS------------------------- : 320
OR106-3  : LTERNAILIISSLLPALINPVVYCTRTKEIRKRLADVFLHKKVVPGQFKVIS------------------------- : 320
OR106-4  : LTERNAILIVVSLVPSLINPIVYCSRTKEIRKILSDVLMRKKISPSNF----------------------------- : 316
OR106-6  : LTERNAIAIVSSLVPPLINPTVYCTRTKEIRKRVSDVFLHSKKIVPRDFKSFPNTLSS------------------- : 326
OR106-8  : LTERNAVVIVSCYVSLLVNPTVYCIRTKGIRKRITDIFLYTQKITK------------------------------- : 314
OR106-9  : LTERNAVLIVSCFVSLLVNPTVYCIRTKEIRKRITDIFLYTQKITK------------------------------- : 313
OR106-10 : LTERNAVLIVSCFVSLLVNPTVYCIRTKGIRKRITDIFLYTQKITK------------------------------- : 313
OR106-7  : LTERNAIMIVSSLVPPLINPTVYCTRTKEIRKRLLDIFLRKKIAPNHLKISSVNLLS-------------------- : 325
OR106-5  : VTDRNAILIAASLVPPLINPTVYCTRTKEIRRRLSDFFLSTKILPNNFK---------------------------- : 316
OR108-1  : PSVRTVCLSLSSVLPPCANPVIYSLKTKEIRSKIVSLLNLRLSVHPLKNVH-------------------------- : 321
OR108-2  : SNVRSVCLSMTSLLTPCVNPILYSLKTKEIRSRVYFLLPQRLSVHPLKSAQ-------------------------- : 326
OR108-3  : PNVGLVCLSLSSLLPPCVNPVIYSLKTKEIRSRVYSLFTRRLFVHPLKNGH-------------------------- : 325
OR110-1  : TNTRIINLSLTSALPPMLNPIIYTFMTEEFMVAVKKRLFKRRNIFPSWK---------------------------- : 319
OR110-2  : TNTRIINLSLTSALPPMLNPIIYTFKTEEFMVSVKKLLKRKIIFPYLK----------------------------- : 318
OR109-1  : PNVQIINNSLTQAIPPMLNPIIYTLKTEEVMQSIKDLYRRSKVNITKQRQMKFASMVN------------------- : 329
OR109-2  : PNTRLINHSLTQTISPMLNPIIYTLKTEEVMQSIKDLYKSSKVSSTVQLKCRRNSKLKLQV---------------- : 336
OR109-4  : LNTRLINSSLTQAIPPMLNPIIYTLKTDEVMQSVKELYKRKVNTTTERRMKWNSRNSKQKIQV-------------- : 338
OR109-3  : ANIRLINNCLTQVIPPMLNPIIYTLKTDEVMQSIKELYNGRNVKNIGQMKCTRRYSKQK------------------ : 334
OR109-5  : PNTRIFNNSLRQVIPSMLNPIIYTLKTEEVMESMKDLYKRRKINTAIERKMKCERGN-------------------- : 329
OR109-6  : PNTRIFNNSLRQVIPSMLNPIIYTLKTEEVMESIKDLYKRRKINTAIKRI--------------------------- : 325
OR109-9  : PNTRIFNNSLRQVIPSMLNPIIYTLKTEEVMESIKDLYKRRKINTAIKRI--------------------------- : 325
OR109-7  : SDIQIINNSLTQTIPPMLNPILYTLKTDEVMQSVKELYKRSRMNTIRVSKTKC------------------------ : 325
OR109-10 : SDIQIINNSLTQTIPPMLNPIIYTLKTDEVMQSVKELYKRSRMNTIRVSKTKC------------------------ : 325
OR109-8  : PNARLINNSLTQTIPPMLNPIIYTLKTDEVMQSIKELYKRSRVYATMEQNRKCHGRKLNDFKVFGALDFKC------ : 343
OR109-11 : PNARLINNSLTQTIPPMLNPIIYTLKTDEVMQSIKELYKRSRVYATMERNRKCHGRK-------------------- : 332
OR111-1  : PNARVISTSLSYAIPPMLNPIIYVLNTGEIKDLIRKMLKSRSAPIRENISK-------------------------- : 326
OR111-2  : PNARIISTSFSYAVPPVLNPIIYVLNTAEIKDIIKKMIKNKSAPIRENISK-------------------------- : 326
OR111-3  : PNARVISTSLSYAIPPMLNPIIYVLNTAEIKYIIRKLFKRRLRSVSDNISK-------------------------- : 326
OR111-4  : TNARIIGTSLSYAVPPLLNPIIYVFNTAEIKDILRKVCKKRLTPVGGNISK-------------------------- : 326
OR111-5  : PNARVISTSLAYVVPPMLNPIIYVLNTAEIKNLLQKMFSKRSAPVREDISK-------------------------- : 326
OR111-10 : PNARVITTSLAYTISPMLNPIIYVLNTAEIKDLLRKMFKNKSAPSKDNIAK-------------------------- : 326
OR111-7  : SNSRVISTSLAYAIPPMLNPIIYVLNTAEIKNTIRKGFKNRSLPIETVSK--------------------------- : 325
OR111-6  : PNARVICTSLAYTIPPMLNPIIYVLNTAEIKDIIWKVFKNKSVAVESISK--------------------------- : 325
OR111-9  : PNVRIISTSLSFTLPPMLNPIIYVLNTAEIKDTIRKKFKKRIFT--------------------------------- : 314
OR111-11 : PNARVLSTSLSYVLPPTLNPILYVLNTAEIKQLIKKIIKNRSTKTVHVAS--------------------------- : 325
OR111-8  : LNARVISTSLAHALPPMLHPIIYVLNTAEIKDIIIKIVRNKCLPVRKNILKLRK----------------------- : 324
OR107-1  : VDLLTAILTVSVTVPAMLNPIIYSLKTDELKERMVKLFRQSKVAQQIIKTEVKALQQ-------------------- : 331
OR104-1  : KAEKIALVMFYSLLPPLMNPFIYFIRIREIRQVFLKCCAQRKRMISSSTTVSK------------------------ : 327
OR104-2  : NNEKIALVMFYSLLPPLMNPFIYFMRIREIRQVFFKFCTQRIMINLKG----------------------------- : 319
OR105-1  : KDFRILLVLIYCLVPPLVNPFIYCLRTQEIRMIVSRWVSRQQALRVACKINVITL---------------------- : 325
OR103-1  : TDLRLAIIMMYSLFPPMFNPLIYCLRTKDVKECLVKRLKISTNVKFNLTKVDISSVSS------------------- : 328
OR103-5  : TDLRLIMIMMYSVLPPLINPIIYCLRTEEVKKVLRQQFKNKQIGNPLQLL--------------------------- : 320
OR103-2  : TDLRIIMIMMYSVLPPLINPIIYCLRTEEVKKVLRQHFSKETNR--------------------------------- : 314
OR103-3  : TDLRLIIIIMSSQLPPMINPLIYSLRTEEDKKILKRPFQNKLIGFPLQLL--------------------------- : 320
OR103-4  : TDLRILINVMSNLLPPMINPLIYCLRTEEITKILKRPFQNKQIGIPLQLL--------------------------- : 318
OR102-1  : PGIRVMLIMWYTLIPPIVNPMIYCFRTKEIKDAIKKKLKDRKVNVQI------------------------------ : 315
OR102-3  : PSFRIAITLCSCLLPPVVNPMIYCFKTKEIKDAIRIKMRHKIVSMPVKMM--------------------------- : 328
OR102-4  : PSFRIAITLCSCLLPPVVNPMIYCFKTKEIKDAIRMKMRHKIVSMPVK----------------------------- : 326
OR102-5  : PSFRIALSLCFCLLPPVVNPMIYCFKTKEIKDALKMKIRHASVNQVIL----------------------------- : 317
OR102-2  : NDTRMVVSMMYSLIPAVINPFIYCFRTKEIKEAIFKRFKKNKVDRKISLG--------------------------- : 327
OR101-1  : PEVRIMIALLYSVLTPLLNPIIYSLRNKELQDAMKRAFCKCAVRSEVARKTVPSVA--------------------- : 316
OR113-1  : SNNRIFISTVYLFFPSCLHPLIYGWRTKEIRQTFLQFIHKAHIFPLEK----------------------------- : 317
OR113-4  : SNNRVFISIVYLFLPCCFHPLIYGWRTKEIRQTFLNFVVNAKVFPKI------------------------------ : 316
OR113-2  : SSNRIFISIMYLLFPSCLHPLIYGWRTKEIRQTFLKFINSANVFPLKK----------------------------- : 317
OR113-3  : SNNRIVISVMYLCFPSCFNPIIYGWRTKEIRQMFLKFIKNKLYPLK------------------------------- : 315
OR114-1  : RDIRVLCTMSYLLFTNFFNPVIYGIRTKEIKAQIVKYLSKNKVGSY------------------------------- : 314
OR112-1  : IDVNTFLGVMYIVFPASVNPIIYGVRTKEIRTALFKIFKNKINKVSIVKVSTIRN---------------------- : 327
OR124-3  : RVVYTFLSIINLTLPPFVNPLIYGIKLGPIRAKIFNIFKGQRLNEL------------------------------- : 310
OR124-2  : HVVYTFLSIINLTVPPIVNPVIYGIKLGPIRAKLLYIFRKLRINNL------------------------------- : 310
OR124-1  : HVEHTFLSIIILTVPPFLNPLIYGIKLGPIRAKILYIFQRPKIK--------------------------------- : 308
OR124-4  : HFVHMFLSVIFLTLPPIVNPVIYGIKLSPIRAKILYIFHHLSISTL------------------------------- : 313
OR123-1  : QEFKNFLSVTFMIIPPLINPVIYGIVLTPIRTKAKHVFQSFRPKHID------------------------------ : 311
OR126-1  : QDLRNFMSLEFLLVPPMLNPLIYGLNLTRLRREVMRCFLKK------------------------------------ : 309
OR126-2  : QSLRNFMALEFLLLPPILNPLIYGLKLTAVRQEVMRLFSKRQM---------------------------------- : 311
OR126-3  : QNLRNFMALEFILIPPFLNPLIYGLNLTAVRQEVIRLFSRKQLEISK------------------------------ : 315
OR126-5  : QSVRNFMALEFLLVPPILNPLIYGLNLTAVRQQVIRLFYKKQVVISD------------------------------ : 315
OR126-4  : QSLRNFMAMEFLLVPPILNPLIYGLKMKTVRQEIINMFFRKTTENI------------------------------- : 314
OR125-1  : ESLRHFLALELVIVPPLFNPLMYGLNIRAVRKRVFTSCGAAKVNILEKVHKFIMH---------------------- : 319
OR125-2  : ESLRHFLALELVIVPPLFNPLIYGLNIRVLRKLAFTSCNAGR----------------------------------- : 306
OR125-8  : ESLRSFLALELVIVSPVFNPLIYGLNVRAIRERVFTSCAESTVNVPGARKRVQQNISHFCDSKISCKVLCCIVFLSK : 341
OR125-3  : DDLRNFLALEIIIVPPLINPVIYGLNLKEIRNRVLKLCTNILKKS-------------------------------- : 309
OR125-5  : ANLQFFLALEMIVVPPFVNPVIYGLKLTEVRKRIFKKFMKVTKQ--------------------------------- : 308
OR125-6  : ANLKFFLALEMIVVPPLVNPVIYGLKLTEVRKIIFKKCMNVIKQMIKNEVHCA------------------------ : 317
OR125-7  : VNLRFFLALEMIVVPPLVNPVIYGLKLTEVRKRILKRCMNVIKIDKA------------------------------ : 311
OR125-4  : DSFQNFLALEMLIVPPLVNPIVYGLKLQEVRKQIFKSFVQIKK---------------------------------- : 307
OR128-13 : VGLSVFLSLEFLLIPPILNPLVYGLSLSDIRKKNICLRNGCR----------------------------------- : 305
OR128-7  : VGLSVFLSLEFLLIPPILNPLVYGLSLSDIRKKNICLRNVCR----------------------------------- : 305
OR128-9  : VGLSVFLSLEFLLIPPILNPLVYGLSLSDIRKKNICLRNGCR----------------------------------- : 305
OR128-8  : VGLSVFLSLEFLLIPPILNPLVYGLGLSDIRRKIFASARYPCRLLKV------------------------------ : 310
OR128-6  : VGLSVFISLEFLLIPPILNPLVYGLSLSDIRKKNICLRKLTK----------------------------------- : 305
OR128-10 : IGLSIILSLEFLIVPPILNPLIYGLNFPEIRKKIMCIVKTCK----------------------------------- : 305
OR128-1  : VGLSVFLSLEFLIIPPILNPLIYAFNLPDIRKKMISLIKPFR----------------------------------- : 305
OR128-2  : VGLSVFLSLEFLIIPPVLNPLIYAFNLPDIRKKMVSLIKPLR----------------------------------- : 305
OR128-3  : VGLSVFLSLEFLIIPPILNPLIYAFNLPDIRKKMISLVKP------------------------------------- : 303
OR128-4  : VGLSVFLSLEFLIIPPVLNPLIYAFNLPDIRKKMISLIKPLK----------------------------------- : 305
OR128-5  : VGLSVFLSLEFLIIPPVLNPLIYAFNLPDIRKKMISLIKPVKF---------------------------------- : 306
OR127-1  : I-LTIIFSVEFLVIPPVLNPLIYGLNLPEIRKKIACLFRRSKIGHFIE----------------------------- : 314
OR122-2  : AVVRVILSLYFLVCPPLINPIMYGVSLTNIKKALKKCIGV------------------------------------- : 307
OR122-1  : AVVRVILSLYFLVCPPLFNPIMYGVRLANIKKALKKMICM------------------------------------- : 307
OR121-1  : ALIRVIISVYFLMCQPLLNPILYGVRLKNIRLACKNFVLSKIGIA-------------------------------- : 312
OR120-1  : KEVNVFMSVHFILIPPLLHPLIYGIRTKEINNSIAKIFRRQIFALGFNLKTEQRCNEALFITSK------------- : 329
OR115-14 : AETRKLAAIILHVIPPALNPIIYGVQSKEIRQKILKLFRNQINAK-------------------------------- : 312
OR115-2  : AETRKLAGIIFHVIPPGLNPIIYGIQSKEIRQKILKLFRNQVNAK-------------------------------- : 312
OR115-13 : AETRKLADIIFHVIPPGLNPIIYGIQSKEIRQKIFKLFRNDLNAK-------------------------------- : 312
OR115-12 : TESRKFGNVMFHVVPPGLNPIIYGLQAKEIKDKMLKLCVKKVNPS-------------------------------- : 312
OR115-10 : SDSRKLSSIIKHIVPPCLNPIIYGLQVKEIRQRLFKLISKSKVNFM------------------------------- : 313
OR115-11 : YENRRIASIVFSVVPPGLNPLIYGLQVKEIRQKLFKFCK-------------------------------------- : 305
OR115-9  : AESRKLGSIMFHIVPPGLNPLVYGLQTKEIRQRFVKLCRRKTSTSLM------------------------------ : 314
OR115-7  : SDNRKLASIMFHIVPPGLNPLVYGLQTKEIRQKVVKLWCRKKSMIT------------------------------- : 313
OR115-4P : SDNRKLASIMFHIVPPGLNPLVYGLQTKEIRQKLVKFWTRNKVNNKIIKS--------------------------- : 317
OR115-1  : SDTRKLASIMFHIVPPGLNPLVYGFQTKEIRQKFVKYLCRK------------------------------------ : 308
```

Figure S1

```
                     TM7          ⇓

OR115-6   : SDSRKLASIMFHIVPPGLNPLVYGLQTKEIRQKTVKFCRSTKRLMSN------------------------------ : 315
OR115-8   : SENRKLASIMFHIAPPGLNPLVYGLQTKEIRQNTSKLCSRNKVNVK------------------------------- : 314
OR115-5   : SENRKLASIMFHIVPPGLNPIVYGLQTKEIRQIIVKRWWKNAVDKL------------------------------- : 313
OR115-3P  : SDNRKLASIMFHIIPPGLNPLVYGLQTKEIRQKMTNLWWRKX----------------------------------- : 310
OR116-1   : ANLQKIMGMLIFLVPPIMNPIVYGLNSHEIRKHILKTFHRKTSV--------------------------------- : 311
OR116-2   : ADLQKIMGMLIFLVPPLLNPIVYGLNSHELRNNLLKVIHRKIAHM-------------------------------- : 312
OR117-1   : PTAQRVCGLMIYAVLPVVNPVIYGLKMKDIRIALFVVLRKRKVVPGRKTDVVKIRK--------------------- : 326
OR118-1   : AFLRRVFSISNFIFPPLVNPLIYGFKTKEIRLKVFNSFKSKINSI-------------------------------- : 312
OR118-2   : AYLRRVFSITVFVFPPIINPLVYGFKTKEIRQKMLTCFKRKMTSL-------------------------------- : 312
OR118-3   : AHLRRVFSISVVIFPPIVNPLIYGFKTKEIRQKIENFYKRKIIRVH------------------------------- : 313
OR119-1   : IHLRKFIAVAAMILPPTMNPVIYGLRTKEIRVKGIKFFHRKVFVS-------------------------------- : 314
OR119-2   : IHLRKFIAVAAMILPPTVNPVIYGLRTKEIRVKGIKFFHRKVFVS-------------------------------- : 314
OR132-5   : INLRYLNYIIVLILPRCLSPLIYGMRDEAVWPLFKYFFCYRSGKVKPSVNVH------------------------- : 322
OR132-1   : INLRYLNYIIVLILPRCLSPLIYGMRDEAVWPLFKYFVCFRSSKIKPSVNVH------------------------- : 323
OR132-2   : INLRYLNYFVVLILPRCLSPLIYGMRDEAVWPLFKYFFCYRSGSIKPSVNVH------------------------- : 322
OR132-3   : INLRYLNYFVVLILPRCLSPLIYGMRDEAVWPLFKYYFCYRSGKVKPFVNVH------------------------- : 322
OR132-4   : INLRYLNFFVVLIIPRCMSPLIYGMRDEAVWPLFKYYFCYRSGKVKPSVNVH------------------------- : 322
OR133-1   : ISFMFFFFVVFMIFPRCLSPLIYGLRDQAFSSLFKYYFAFGLKKPSKCNTEMHLGGGS------------------- : 320
OR133-2   : LHVMYFCFVVFMIFPKCLSPLIYGLRDQAFSCLFKYYFTFGLKKQNKCNTEIHLVGGG------------------- : 322
OR133-3   : MNVMYVGFIVFLIFPKCLCPLIYGMRDQAFSCLFIYYFTFCKGGTVKPATTEN------------------------ : 315
OR133-4   : INVMYFCFVVFIMFPKCLSPLIYGMRDQAFSCIFLYYFTFGKGGTVNPVIKKQHA---------------------- : 317
OR133-6   : VNVMYFCFLVFIIFPKCLSPLIYGMRDQAFSCIFMYYFTFGKGGTVNPVIKEQHA---------------------- : 317
OR133-5   : LHAIYVCFLVLIMFPKFLSPLIYGTRDQTFSCIFIHYFTFGKGGTVKPVIIEQCLVAII------------------ : 321
OR133-7   : LNVMYFCFLVFIIFPKCLSPLIYGMRDQAFCCLFKYYFTFGKGKIKPVTIELHV----------------------- : 316
OR133-8   : VNVMYICFVVFVILPKCLSPLIYGMRDQVFFCLFKYYFTFGRDKVKPVIIELHV----------------------- : 316
OR133-9   : INVMYFCFVVFIVFPKCLSPLIYGMRDQAFCCLFKYYFTFGKGKVKPVLIELHV----------------------- : 318
OR137-7   : KKINFFNYLLTSILPRLLTPLIYGVRDKQFFNHIKALLTCKLSAVNVDPIKQ------------------------- : 309
OR137-1   : RRIRILNYLLTNILPRLLTPLIYGVRDKQFYNHIKALFLCKLSVVNVNSIKQ------------------------- : 310
OR137-2   : ARLTFLNYLLTNILPRLLTPLIYGVRDKHFNKHMKGLLSCRLHAVKVGSVKR------------------------- : 314
OR137-3   : SKITFFNYLFTNLLPRLLSPLIYGVRDKHFLKYLKGLFLCRLFHVKVQPAKS------------------------- : 310
OR137-8   : SKLLFCNYLLTNLLPRLLTPMIYGVRDKVFYSHMKGIVACKLVIVKVESTKG------------------------- : 310
OR137-4   : SKLLFCNYLLTNLLPRLLTPMIYGVRDKVFYSHMKGIVACKLVIVKVESTKG------------------------- : 310
OR137-5   : SILLFCNYLLTNLLPRLLTPLIYGVRDKVFYSHMKGIVACKLVIVKVESTKQ------------------------- : 310
OR136-2P  : TKITFFNYLITNIMPRLLSPLIYGVRDQRFLKQLKEYFTCKVAIVKILPSKL------------------------- : 308
OR136-1   : TKIAFCIYVFTNIMPRLLSPLIYGVRDQKFVKQMKEYFTCKVFIVKVVPSKL------------------------- : 317
OR135-2   : LLIMFCSFVLTSIFPRLPSPLVYGSRDQRFKKYMKSSLMCGSDNRTIKPSH-------------------------- : 310
OR135-1   : QIIYYCTFILTSILPRLLSPLIYGLRDQTFKKYMKSSFMCSTKTFVSPEK--------------------------- : 306
OR140-1P  : TTILFLLFLLTNILPRLLSPLIYGLRDQKFLERIKVFYSCKHVKTQIITIKKTAV---------------------- : 312
OR134-1   : LEIRFANYLIVYILPRFLSPIIYGVRDKKFRKYLRRYFVCGVSTMETRVECKDGD---------------------- : 315
OR129-1   : ADMSFLYYVCFNLFPRFLSPLIYGIRDEKLKSYIRKVLPCYGARVDPHKLETK------------------------ : 315
OR131-1   : LNVRYSNFILFWILPRSLSPLVYGLRDKKFYYALKYYAFCGVFVVSKHKVRDIKISRRKRC---------------- : 321
OR131-2   : INVKFSNFMLFWIFPRCLSPLVYGLRDKKFYNALKYYAFCGIYVYKKHKIKDSKTIGGALSISIHN----------- : 326
OR130-1   : LTVRFINFLAFSITSRAVSPLVYGFRDEKFYTAMVYFIRCKNNTISQLHE--------------------------- : 315
```

Figure S1
