# Supplementary material for: The odorant receptor repertoire of teleost fish
Source: BMC Genomics. 2005 Dec 6;6:173. doi: 10.1186/1471-2164-6-173 (PMC1325023; doi:10.1186/1471-2164-6-173)
Supplement: Additional File 3 — Figure S2. Multiple sequence alignment of fugu OR amino acid translations. [file 1471-2164-6-173-S3.pdf]

```
                                  ⇓          TM1                           TM2
                   10        20        30        40        50        60        70        80        90       100       110
          ....|....|....|....|....|....|....|....|....|....|....|....|....|....|....|....|....|....|....|....|....|....|
OR.scaffold_1026.1   ----------MENYTFNSFTLQLEGLKVTE-VSMYPVFFFFLVSYVFIMLSNIAIIALIFIDSSLHQPMYLLFCNLPFNDVLGNSILIPRLLVDLLNPPSERLITYYECV
OR.scaffold_8298.1   MNFCFSGFRLMDNYTFNSFTLQLEGLKVTE-VSVYPVFFFFLVSYIFIIVANIAIVALIFIDSSLHQPMYLLFCNLPFNDVLGNSILIPHLLMDLLRPPSERLITYYECV
OR.scaffold_1026.2   ----------MENYTFNSFTLQLEGLKVTE-SSVYPVFFFFLVSYIFIMLSNIGIVALIFIDSSLHQPMYLLFCNLPFNDVLGNSILIPRLLMDLLHPPSERLITYYECV
OR.scaffold_4563.2   ----------MENFTYNSFILQVEGLTLTE-GSVYPAFFLLLFCYLFIIVANVGIATLIFIDSSLQQPMYLLFCNLTFNDILGNSILIPRLLVDSLRPPSERLITYYECV
OR.scaffold_4563.1   ----------MNNWTLNSDVLLLEGLKVTR-RSSFPAFLLLLLIYIFIMVSNMSLMFLILMERTLREPMYLLFCNMSVNDVFGATTIIPRLLRDIFLPSSERFILYNHCA
OR.scaffold_142.1    ---------MENRSFGLSSELTLDTFLIPP-QGKYPIFFLGVTIYCFGVFCNMTLLTLIILQRNLHKPMYFILFSLPLNDLIGITAMLPKVLSDIVMET--NRVHYGLCV
OR.scaffold_142.2    -----------------------------------------------------------------------------------------------XLS------------
OR.scaffold_142.3    ---------MYANISLPVSMVQLENLNLPH-IYVYPTLIITTLLYMVTMFCNMMVLLAIARCKELHQPMFILLFNLPISDMIGTTAFLPQLLWSIVTQY--RSIPYTACI
OR.scaffold_142.5P   ----------MDDLYNISSVLELRSFNLPP-EAVFPAFLFATLSYMVILFCNLVLISVIVLNKSLHQPMYMILLNLPVNDLIGSSALFLQVIKDILTKS--RTIHYSSCV
OR.scaffold_3630.2   -----------MDNRANVTYITVEGYVELQ-KYRYLYFVIMLTAYILIICSNSTIVFLICFHRNLHEPMYVLIAALLINSVLYSTAIYPKLLIDFLSE--EQTITYSVCL
OR.scaffold_5510.1   -----------MDNRANVTYIIVEGYVELQ-KYRYLYFVIMLTAYILIICSNSTIVFLICFHRNLHEPMYVLIAALLINSVLYSTAIYPKLLIDFLSE--EQTITYSVCL
OR.scaffold_8617.1   -----------MDNRANVTYITIEGYVELQ-KYRYLYFVIMLTAYILIICSNSTIVFLICFHRNLHEPMYVLIAALLINSVLYSTAIYPKLLIDFLSE--EQTITYSACL
OR.scaffold_5887.1   -----------MDNRANVTYITVEGLCGTA-EIQISVLVIMLTAYILIICSNSTIVFLICFHRNLHEPMYVLIAALLINSVLYSTAIYPKLLIDFLSE--EQTITYSVCL
OR.scaffold_4429.1   -----------MDD-PKVTYITFDAYVEMQ-KYRYLYFVIMLSAFVTILYSNGTVVYVIYAHENLHQPMYVFIAALLVNSLLYSTAVYPKLLVDSLSD--RQVITYPVCL
OR.scaffold_4429.2   -----------MDEGVNVTVITLGGFTHVQ-QYGYIFFCLILLMYLLMVFCNCSIIGLILSQPNLQQPMYVLIAALLINSVLCSTAVHPKLLMDLVSE--NQTASPSFCH
OR.scaffold_7743.1   -----------MNS--STVLITFTTYSSFG-VFRGPLVTCILVLYVSVLCVNILLVVVIYTEPRLHRPMYVLLAHLALSGLVGSTSVCPTILRHLLLS--RQVTSLRGCL
OR.scaffold_117.1    -----------MDNFTTPPYFNLTMFLNIG-QYRYLAFVLCFILYASIVFANVIIIVVISRENALHEPMYIFIMCLSVNSLYGSAGFFFRFLKDLLSS--THLISRWGCF
OR.scaffold_2346.5   -----------MENNSASFHFNLTMFVNIG-HYRYLAFVFCLLLYSFMISANFVIILVVSRERTLHEPMYIFIALLSVNSLYGSTGFFPRFLMDLLLD--THFMSRSACF
OR.scaffold_2346.4   -----------MGN-ETVLYFNLTMFIMIG-NVRYLAFAFCLLTYALIVFSNVTMIWIITQERSLHEPMYIFIAFLSVNSLYGSAGFFPRFLMDLLSD--VHLISYAACY
OR.scaffold_2346.6   -----------MDNASDVTMFTLSGFNVII-NYRFLLFALTFVCYCVIVQVNVTLIVAIIVDKSLHEPMFIFLCNLCINSLYGTAGFYPKFLIDILST--SHVISYAGCL
OR.scaffold_3763.1   -----------MDNASEVTIFTLSGFNGIV-NYRFLLFAVTFVCYCVIVQVNVTLIVAIIVDKSLHEPMFIFLCNLCINSLYGTAAFYPKFLIDILST--SHVISYVGCL
OR.scaffold_3763.2   -----------MDNASEVMIFTLSGFNGIV-NYRFLLFALTFVCYCVIVQVNVTLIVAIIVDKSLHEPMFIFLCNLCINSLYGTAAFYPKFLIDILST--SHVISYVGCL
OR.scaffold_4133.1   --MVTTLSHLVMDNASDVTMFTLSGFNGII-NYRFTLFALTFVCYCVIVQVNVTLILTIIMDKSLHEPMYIFLCNLCINSLYGTAGFYPKFLIDILST--SHIISYAGCL
OR.scaffold_6828.1   ----MINSYFVMDNTSEVTIFTLSGFNVIV-NYRFTLFALSFVCYCVIVQVNVTLILAIIVDKSLHEPMFIFICNLCINSLYGTAGFYPKLLIDILST--SHVISYAGCL
OR.scaffold_6765.1   -----------MDNASDVTMFTLSGFNGIV-NYKFTLFALTFVCYCVIVQVNVTLILTIIMDKGLHEPMYIFLCNLCINSLYGTAGFYPKFLIDILST--SHVISYAGCL
OR.scaffold_123.1    ----------MSDNTSVVNMFTLSGLGGIT-NYKITLFVFTFLYYCVILQVNLTVILTIIVDKSLHEPMYIFLCNLCINGLYGTTGFYPKFLIDILST--SHVISYVGCL
OR.scaffold_117.2    ----------MMENVSAVTLLTLSGLDFKL-EYRIVLFLLTLLWYMMIIFGNVLLIVIILVDKKLHEPMYIFLCNLCINSLYGTIGFYPKFLLDLLS---SPVISYAGCM
OR.scaffold_123.2    ----------MMDNASAVRIFTLSGLNETM-NYRIAIFSVTFLYYCVVLFVNISLIAIIFLDENLHEPMYIFLCSFCINGLYGASGFYPKFLFDLLSF--SHEISYEGCL
OR.scaffold_2346.2   ---MFLTETHKMTNKTN--IFYLSGLNDLT-TNRGLIFTFIFLCYSLIWIVNGALILVIILEEKLHEPMYIFLCNLCINSLYGTAAFYPKFLYDLVTN--NHTISYAGCI
OR.scaffold_2346.3   -----------MPNRSSIVVFSLSGFNATV-KYRNTLFALTFLCYFLIIVVNICLILTLIIIEKKLHEPMYIFLGSLCFNGLYGATGFYPKFLSDLLSD--DHLISRVGCF
OR.scaffold_5287.1   -----------------------------------------------------------------XKPIYIFLCNLCFNGLCGASCFYPKLLHDLLAD--SQVISYGGCL
OR.scaffold_2346.1   -----------MENKTKNVYFVLAAYGDIG-ELKYLYFGIMLFWFMSIWMVNSVLILIIYKDKRLHEPMYIFVCHLFVNEISGSTSLYPLMLSQMLSD--THKVTVPWCF
OR.scaffold_7149.1   -----------MENKTETMYFVLAAYGDTG-ELKYLYFGIMLFWFMSIWMVNSVLILIIYKDKRLHEPMYIFVCHLFVNEISGSTSLYPLMLSQMLSD--THKVTVPWCF
OR.scaffold_5511.2   -----------MLNSTSVFYFTLGAYFPVG-NLRYLYFLLTGLLYVVVVVVNVSLIVVICMNRSLHEPMYVLLCSLFVNELYGSTALFPFLLLQILSD--VHTVSASFCF
OR.scaffold_119.1    ------------MDNTTVLTFTMTAYAVMK-NYKSGIFTVFLLLYLLILTLNLVILMLIHQTKELHQPMNVFTFLLCLNQVYGSTALLPAVMALLLST--TYEMPVKCCL
OR.scaffold_5287.2   ------MLDGSLGQNFSHSTFSFRGFPELH-CYRRLLALPFSVSYLSVLLGNCLLVYVICSVEHLHSPMYLLICSLCVVDVLAATAILPSMLLSLLFD--VDTISLASCL
OR.scaffold_142.6    ----------MIDNVTGTESFFILGFPGLAPEYYRPVSALFLIIYLVIAFGNIFILTFVMCQKSLQKPMYLVFCHLALIDLTFSTVTIPKIISKYWFS--GGDISFYGCF
OR.scaffold_142.7    ---------MLLTNVTRIQYFYIVGFLGLSPDYYGHVSAALFLVLSAIVVGNVFILVFVKCERSLHKPTYLIFCHLASTDLLFGLVTLPKIISKYWFG--DSLISFGGCF
OR.scaffold_3154.1P  -------------------------------------------------------XVETCFPCKCPNP-FQAPKNSDIIFLIN-------HKKVFLLK--DGYISYNSCL
OR.scaffold_3905.1   -MELFNAAPGKNVSFVHPPYFIISGLKGIP--HIKFYYVFLFFVYIVSVLGNSVVMAVICLDHNLRTPKYVAVFNLAFVDLFGNSALVPKLLDIFLFD--HRHIPYNDCL
OR.scaffold_4479.1   ------------------------------------------------------------------------------------------MIKTFLLK--DNFFPFNICL
OR.scaffold_4479.2   --MFLLRTVSNGSIIIHPPGFYVVGFEVFP--HIRFYFIFLAFVYVVTVFFNCLLIYVIVSNRCLHTPKFLAVVNLAVIDIVLNSTAIPSMIKTFLLK--DNFFPFNICL
OR.scaffold_6269.1   --MFLLRTVSNGSIIIHPPGFYVFGFEVFP--HIRFYFIFLAFVYVVTVFFNCLLIYVIVSNRCLHTPKFLAVVNLAVIDIVLNSTAIPSMIKTFLLK--DNFFPFNICL
OR.scaffold_4208.2   --------------------------------------------------------------MFYMNPRYILFIHLVINDLIQVNVTIMLFVISYTIYR----INICMCW
OR.scaffold_4208.3   ------------------------XTPRLKFTSVDPGEAGSHRLITRCGLTCCVCPRCPVPQVFYTNPRYILFFHLVVNDMIQVMLTVMLFIISYVLYK----INVSFCG
OR.scaffold_4208.1   -------------------------------------------------------------XIFNSNPRYILFIHLVINDMLQLFLSTFLHVITYVFVR----VNVSFCS
OR.scaffold_11306.1  ---------------------------------------------------------------------------MVINDAIQLTVTTLLFILSYVLYK----INVAVCC
OR.scaffold_59.1     -----------MDEIQPLVNFTVEQQYQGLLERMMFSSLTTVPCCAFLYINGIMLFSLRSKTVFCETSRYILLFNLLFADSIQMVFSQVLYILATCLIK----MSYPLCG
OR.scaffold_6030.1   -----------MNEIWSLVNVTVEQQYQGLLERMMFSSLTTVPCCVFLYINGIMLFSLRSKTVFCETSRYILLFNLLFADSIQMVLGQALYILAVCLIK----MTYPVCG
OR.scaffold_144.1P   -----------MDEIQSFDNVTVEQQYRVFLERVMFSSLTTVPCCVFLYINGIMLFSLKSKTVFCETSRYILLFNLLFADSIQMALSQLLHICSCLFAX----MSYPVCG
OR.scaffold_7573.1   -----------------------------------------------------------------------------------MALSQVLYILAACLIK----MSYPVCG
OR.scaffold_6392.1   ---------------MSDNASVAGGVSLQARIFEQIIITQFIVSIFVSVNLLLIATVFSNHVFLSTMRYILFVITLVSDSVFLILSNLLLILYNIYFT----MQVSLCI
OR.scaffold_7903.1   ---------------MSDNASVAGGVSLRVQIFERIIITQLIVSIFVSDNLFLIATVFSNQVFLSTMRYILFVITLVSDSVFLIIANLLLILHSTYFT----IEVSLCI
OR.scaffold_26.3P    ---------------MSDNASVAGGVSLRARIFEHSLLHSSLCQFSFMINLLLIATVFSNQVFLSTMRYILFVITLVSDSISLIIANLLLILHSTYFT----IEVSLCI
OR.scaffold_6818.1   ---------------MAANSSEPAGQSSARKINSRVILVQVLVLVFLCINFLLIVTFSMRHFFYTTMRYILFAVTLLSDSVLLLLTDVMLILSYFAVT----IEMWVCV
OR.scaffold_217.1    ------------------MNVTTVDAPAPPSHATLKALLSVLPCLFFFYINAVMMVALFQKPQLLESCRYILFGHLLLTESLQLLFSVLLYLFALTKT----AMLSYICI
OR.scaffold_26.4     -------------------------------------------------------------XADQDTSRYILXAHKMINDTLQVLSSVLLFLFVMAQVK----VALIYCV
```

Figure S2

```
                                TM3                 TM4                                     TM5
                          120       130       140       150       160       170       180       190       200       210       220
                      ....|....|....|....|....|....|....|....|....|....|....|....|....|....|....|....|....|....|....|....|....|....|
OR.scaffold_1026.1    AQAFTSHMFGTTSHTILMIMAFDRYVAICNPLRYTVIMSHRMVIKLTVSAWAVAFVLVGILLGLTIRLNRCR-TLITNPYCDNASLFKLSCDSVVIN-----NIYGLMFT
OR.scaffold_8298.1    AQAFTTHMFGTTSHTVLIIMAFDRYVAICNPLRYAAIMNHRMVIKLTISAWAVAFVLVGILLGLTIRLNRCR-TLITNPYCDNASLFKLSCDSVVIN-----NIYGLTFT
OR.scaffold_1026.2    VQAFTTHMFGTTSHTVLMIMAFDRYVAICNPLRYAAIMNHKMVIKLTVSAWAVAFVLVGILLGLTIRLNRCR-TLIANTFCDNASLFKLSCDSVVIN-----NIYGLTFT
OR.scaffold_4563.2    VQAFTTHMFGTTSHTVLMIMAFDRYVAICNPLRYAAIMNHRMVIKLTVSAWAVAFFLVGILLGLTIRLNRCR-TLIKNPYCDNASLFKLSCDSVVIN-----NIYGLTFT
OR.scaffold_4563.1    LQAFCNNYHASVSHTVLMIMAFDRYMAICNPLHYSSIMTNRMMLNLSAWAWGSVFIMVAILIGLSVRLSRCR-WIVENPFCDNATLFKLSCESILIN-----NIYGLAYT
OR.scaffold_142.1     FQAFILHMYGGGILFILAAMSFDRYVAICMPLHYNAIMVPRVVVFIISLVWGLDFSLILLLFVLQMRLPHCK-SVIMNVFCDNPSLLKLTCANTTIN-----NVVGLFNT
OR.scaffold_142.2     -SVFLLACLPG-----LDPACPQRFDPACL----------------------------LLFVLQMRLPHCK-SVIMNVFCDNPSLLKLTCANTTIN-----NVVGLFNT
OR.scaffold_142.3     TQAFLIHMYGTGNLLILSIMAYDRYIAICFPLRYNEIMSPLTLVKMILSVWIVTISVIATMFLLHRQYEICR-TNIVDFYCNNPSLLKIMCGETTVS-----SYYGLMVI
OR.scaffold_142.5P    TQAFFIHIYAGGTVFILTAMAYDRYIAICNPLKYNTIMTHAHIFRIITCLWLTNVALMSVLFFLLLRLPRCR-SVLTHPYCDQPSLLALVCADVTVN-----NIYGLLTV
OR.scaffold_3630.2    FQWFMFYSLGGSEFLLLAVMAYDRYVAICKPLQYSAIMRRRTVTIFLLLAWIAPTAQVAVPTIMNANRKLCN-FIFKGIICNSTVFN-LHCQRS-----ETFNIYGLMVL
OR.scaffold_5510.1    FQWFMFYSLGGSEILLLAVMAYDRYLAICKPLQYSAIMRRRTVTIFLLLAWIAPTAQVAVPTIMNANRKLCN-FIFKGIICNSTMFN-LHCQRS-----ETFNIYGLMVL
OR.scaffold_8617.1    FQYFMFYSLGASEFLLLAVMAYDRYLAICKPLQYSAIMRRRTVTIFLLLAWIAPTAQVAVPTIRNANRKLCN-FIFKGIICNSTVFN-LHCQRS-----ETFNIYGLMVL
OR.scaffold_5887.1    FQWFMFYSLGGSEFLLLAVMAYDRYLAICKPLQYSAIMRRRTVTIFLLLAWIAPTAQVAVPMIRNANRKLCN-FIFKGIICNSTVYN-LHCQRS-----ETFNIYGLMVL
OR.scaffold_4429.1    FQWFMFYSFGGSEFLLLAVMAYDRYVAICKPLQYSAIMRRRTVGIFLAVSWFLPFCCNAAAVALSARQKLCR-FSINGILCSSTVHS-LHCQDSNHTLRVAMSAYGLVLL
OR.scaffold_4429.2    LQFFLYYWLAGAEFLLLACMAFDRYVSICQPLHYHRIMRNRTLGSLLLTSWLVPACQAAAMAALSSRLQLCR-WTLDGIFCNNSLYK-LHCATP-----RAVSVYGVVAL
OR.scaffold_7743.1    TQVFFINVYAGSIYCFLALMAYDRYVSICKPLLYHAVMRPVRVRLMVALVYLVLSSTAAVQVYLTSTLALCR-HSVGKLVCDSLAISNLSCERT-----TLISVYGLCCA
OR.scaffold_117.1     AQIYVIYTYASYELTLLSIMAYDRYVAVCQPLHYHNKITKRSVMRLILLASLYPAFSVAACVYLSSMLPLCG-NAIPKVFCANWPVVKLSCVPTWLN-----NLIGMLVS
OR.scaffold_2346.5    TQIYVFYTYISFEMTILGIIAYDRHVAICNPLHYHRKMTHKTVVLLASSACLIPVFTIAVGLYLSVRLPLCG-NEIKRVFCGNWNVVKLSCVSTIVN-----NIYGMFVT
OR.scaffold_2346.4    SQIYVIYTYATYEVTILSIMAYDRCIAVCQPLHYSRKLTPKTVCALSVFAWVFPAFNLSVTIIMLVRIPLCG-NNVQKVYCASWNIVKLSCVSNFNN-----NTVAMMWV
OR.scaffold_2346.6    VQSLVVNSSVCAEVSFLALMAYDRYVAICQPLVYHSVMTIQRICLLVFVAWILPVGQLLITTMLTSTLRLCG-SQISRIYCVNYVIRRLGCTPSIT------TVFSPAFT
OR.scaffold_3763.1    VQSLVVNSSACADFSFLALMAYDRYVAICRPLVYHSVMNPQRMCLLVFAAWILPLGQMLTTIISTSTLRLCG-SHISRIYCVNYVIRRLECTPSIT------TVIFPAFI
OR.scaffold_3763.2    VQSFVVNSSACTDFSFLALMAYDRYVAICQPLVYHSVMNPQRMCLLVFVAWILPVGQLLITILITSTFRLCG-SQISRIYCVTYVIRKLECSPSLTVI----TVFSPAFT
OR.scaffold_4133.1    VQSLVVNSSACAVFSFLALMAYDRYVAICQPLVYHSVMNPQRMCLLVFAAWILPVGQLLITITLTSTLRLCG-SQISRIYCVNYVIRRLECTPSIT------TVFSPAFT
OR.scaffold_6828.1    VQSFAVNSSACADFSLLVLMAYDRYVAICRPLVYHSVMNPQRVSALVFAAWILPLGQIFITTTSTSTLTLCG-SHLVRMYCINYVIRRLECTMSIT------TAFFSVFI
OR.scaffold_6765.1    VQSLVVHSSVCADFSILVLMAYDRYVAICQPLVYHSVMNPQRVSALVFAAWMLPLTQFIVITVSTSTLRLCG-SQISKLYCVTYVIRKLQCSSSIS------TVIFPAFI
OR.scaffold_123.1     LQAFVLHSSACADFSILVLMAYDRYVAICRPLAYHSVMNPQRVYLLIFITWLIPFCSLFFNTITTSRYRLCG-SHIQRIYCVTYMIAKLSCSASIA------NAVLAYIN
OR.scaffold_117.2     LQGYVIHSSTCGDFSILALMAFDRYVAICRPLVYHSVMTRPRVSLLVFLSWFIPLHCMFMNSATLLGVKLCS-SHINKVYCVNWMIVTIACSPPKV------NSFIGYFN
OR.scaffold_123.2     LQAFVMYPFACCEMSILAVMAYDRYLAICQPLQYHSFMTRRRISVLVCSSWLLPLCIFSLSIFLTSTLNLCG-SKIQKLFCVNWVIYELACSESNTLA----NNIISHAT
OR.scaffold_2346.2    LQVFVIYSYASTDFSILALMAYDRYLAICRPLEYHSVMTKHKVVLLVCFSRLVPWLCHTVLIIMTSLLQLCD-SHIDKLYCANWSIVKLSCNSITA------NNIVGYIV
OR.scaffold_2346.3    VQIYVIYSNAKIDYSILVVMAYDRYVAICRPLEYHCVMSKQNIVLLLGLSWLVPLCCETLVISLSSSLELCG-SHIDKLYCENWAVVKLACGATTA------NDIVGMVL
OR.scaffold_5287.1    TQLFSVYCYVFCEFTSLTVMAYDRYLAICRPLQYHTLMTVQKVLQLLLLTWSFSLLETSVGTVLTARLPLCK-HHLPRIFCTNWEVVKLSCFDSNTTI----NNIYGFIL
OR.scaffold_2346.1    LQMCYIYTSASLEFCSLAAMAYDRYVAICYPLQYSLIMNTERVCKIILGVWGYSVMNFLVLFSFFIHLDFCG-NVINKVFCDYHLIIQLACSVSAID-----KISDLIFA
OR.scaffold_7149.1    LQMCYIYTSASLEFCSLAAMAYDRYVAICYPLQYSLIMNTERVCKIILGVWGYSVMNFLVLFSFFIHLDFCG-NVINKVFCDYHLIIQLACSVSAID-----KISDLIFA
OR.scaffold_5511.2    LQIFCVYTYGAIEYMNLAVMSYDRYLAICHPLQYHTRMTCHRAFVWIAALWFYSVVKFLITLSLNMRLALCR-NLINSLYCQNYLVVKLACSDTRIN-----NIYGLFGI
OR.scaffold_119.1     TQVYFLHTYASLEFCVLALMAYDRYLAICRPLHYHTIMSHSTMRKLVTLSVVYPNVAFLCYYSLTLQLTFCR-RFIPKLYCVNMEVVKNSCSIPAHI-----NIVGLLLI
OR.scaffold_5287.2    TQMFFTHFLSSLESTLLLAMALDRYVAICQPLRYARIINLSMLARLLLFTLVRSGSIMAALVGLASSLRFCGSNTIQHCYCDHMALVSLACGSTERN-----SAIGLVVI
OR.scaffold_142.6     AQMFFVHYLGSVTSFILLVMALDRFISICFPLRYTALVSKKVISALCGIAWFIPVSLLVAVIVQALALPYCKSNVIVQCFCDHISITNLACGEGVQS----VRVGTLCIA
OR.scaffold_142.7     VQMYFVHFLGATHSFVLMVMALDRFIAVCMPLRYTVFITSATVSVLCGISWFMPMSWMVGIVLHALSLPYCRSNIIVQCYCDHIAITALGC-ENVRD----VQVVAFGLA
OR.scaffold_3154.1P   TLFFFLFLSLSMQALNLVVLAYDRLIAIMFPLHYPMMVTNRFMVSFIAVLWFFAIILVLISAALLTRLSFCESVVINSFFCEHGPLXKLGC-NDISP----SLIYGTLVS
OR.scaffold_3905.1    TFLFFCYTCLSMQALNLVALAYDRLVAIIFPLHYQMRVTHRIMMSLIASFWAFIIVAVFIAVGLITRLSFCKSVVIHSYFCDHGQIYRLAC-NDHFP----SYVVSCLYP
OR.scaffold_4479.1    LQMFVYYSFITLESFALAILAYDRLIAICFPLQQNSINTLRSMSCIVAVTWSYSLGLGAFSTGIMTRLSFCKSVTVRSYFCDYAPTFRLAC-NDYTL----HWAVASTAS
OR.scaffold_4479.2    LQMFVYYSFITLESFALAILAYDRLIAICFPLQQNSINTLRSMSCIVAVTWSYSLGLGAFSTGIMTRLSFCKSVTVRSYFCDYAPTFRLAC-NDYTL----HWAVSSTAS
OR.scaffold_6269.1    LQMFVYYSFIILESYALAILAYDRLIAICFPLQQNSINTLRSMSCIVAVTWSYSLGLVAFSTGIMTRLSFCKSVTVRSYFCDYAPTFRLAC-NDYTL----HWAVSSTAS
OR.scaffold_4208.2    VFMLLAVVATENSPLNLACMALECYSAVCFPLHHLQVWTIRRTLTLILLMWTTTTVSATSDLVITLATEHLT-IVHSQVFCLRHTAFPLDVITT----------KRDVTY
OR.scaffold_4208.3    VFILLALITTENTPLNLACMALECYIAVCFPLRHGQICTIRRTLLLIGLIWTTSMFSVLPDLFVTLATQPLD-FFYSQVFCLRETAFPNPHIIR----------KRDITY
OR.scaffold_4208.1    IILVTIILTTLNTPLNLASMAIERYIAICLPLRYGHICTVRKTYGLIGLMWVLSVFAILPDFFALLTTQPLT-FFHSPVICLREQVFRSAYSVV----------NREVSH
OR.scaffold_11306.1   VFILLAVFTTRNTPINLASMAIERYISICEPLRHAQICTVRRTYIVISLIWFMCVAPDITDLFVTLATESLS-FFHQSVFCLRHNIFKDPVLAH----------KRQAFD
OR.scaffold_59.1      IVNVVGVITSDISPLTLVVMSLERYVAVCYPLRHAAVITIRNTGLMIYVIWAFSLLSGLIR-VSLVNYPELK-GLQMEEFCSSITLFVGPLSDT----------YDRAYT
OR.scaffold_6030.1    ILTALGVLTSNISPLTLVVMSLERYVAVCYPLRHAALITIGNTGLMISVIWAFSLLSVLIQ-VSMVDYPEME-SLQMEVYCSSITLFVGPLSDT----------YDKAYT
OR.scaffold_144.1P    ILSVVSVLTSDISPLTLVVMSLERYVAVCYPLRHAAIITIRNTVLIISVIWAFSLLSGLIR-VSLVDYPDLK-SLQMEVYCSSITLFVGPLSDT----------YDRAYT
OR.scaffold_7573.1    ILSVVGVLTSDISPLTLVVMSLERYVAVCYPLRHADVITIRNTVLMIFVIWAFSLLSGLIR-VSLVDYPELE-SLQMEEFCSSITLFVGPLSDT----------YDKANT
OR.scaffold_6392.1    IFYLIAVSVCVVTPVTLTAMTLERYVAICLPLRHPELCSLHNTQKCILIILTVSSVPCFIILSTFIAAASSS-VYTQHKRCSMEMFVSLPWQNH----------FKFAVY
OR.scaffold_7903.1    VFYLIVCLSVFVTPVTLTAMTLERYVAICLPLRHPELCSLHNTQKCILIILTVSSVPCFIILSTFIAAASSS-VYTQHKRCSMEMFVSIPWQNH----------FKFAVY
OR.scaffold_26.3P     VFYLIVSLSVFVTPVTLTAMTLERYVAICLPLRHPELCSLHNTQKCILMILTVSSVPCFIILSTFIAATSSS-VYTQHKRCSMEMFVSIPWQNH----------FKFAVY
OR.scaffold_6818.1    TLYVVLALYTFVTPVTLTVMTLERYVAICLPLRHSELCSMRRALHCILLVHGVSFLPCAVVLSMLFASASYN-FYTQVKICSVEILIIHQWQSH----------LRSAIS
OR.scaffold_217.1     SVILFASLTLTMSPLNLAVMSLERYIAIYCPLKHVHIVTSRTTGVAIAFIWTTGSLNSLTHWGLFISLKNTS--FIVPRFCNRGNLFPLPIFSS----------INDAFT
OR.scaffold_26.4      PLLFLSTTTFQNTPLIILATMSLERYVAIFYPLQRPAAWRSDRIWAIILSLWLISSIFPIINYSIGKYDPSVS-VLYTRVLCKVSTVNSSPVQVL----------FKAAVS
```

Figure S2

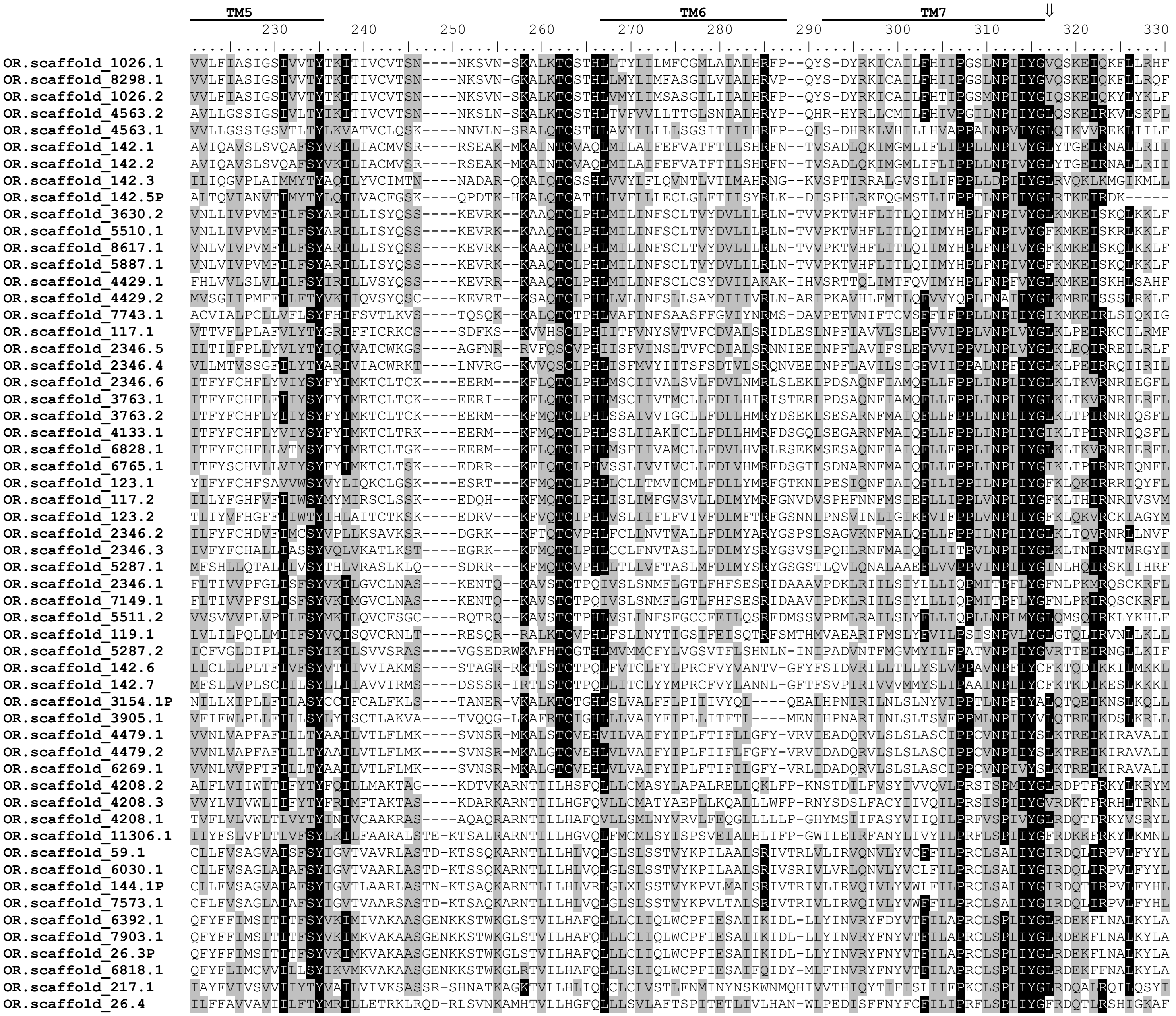
TM5
TM6
TM7
230 240 250 260 270 280 290 300 310 320 330
OR.scaffold_1026.1 VVLFIASIGSIVVTYTKITIVCVTSN----NKSVN-SKALKTCSTHLLTYLILMFCGMLAIALHRFP--QYS-DYRKICAILFHIIPGSLNPIIYGVQSKEIQKFLLRHF
OR.scaffold_8298.1 VVLFIASIGSIVVTYTKITIVCVTSN----NKSVN-GKALKTCSTHLLMYLIMFASGILVIALHRVP--QYS-DYRKICAILFHIIPGSLNPIIYGVQSKEIQKFLLRQF
OR.scaffold_1026.2 VVLFIASIGSIVVTYTKITIVCVTSN----NKSVN-SKALKTCSTHLVMYLIMSASGILIIALHRFP--QYS-DYRKICAILFHTIPGSMNPIIYGIQSKEIQKYLYKLF
OR.scaffold_4563.2 AVLLGSSIGSIVLTYIKITIVCVTSN----NKSLN-SKALKTCSTHLTVFVVLLTTGLSNIALHRYP--QHR-HYRLLCMILFHIVPGILNPIIYGLQSKEIRKVLSKPL
OR.scaffold_4563.1 VVLLGSSIGSVTLTYLKVATVCLQSK----NNVLN-SRALQTCSTHLAVYLLLLLSGSITIILHRFP--QLS-DHRKLVHILLHVAPPALNPVIYGLQIKVVREKLIILF
OR.scaffold_142.1 AVIQAVSLSVQAFSYVKILIACMVSR----RSEAK-MKAINTCVAQLMILAIFEFVATFTILSHRFN--TVSADLQKIMGMLIFLIPPLLNPIVYGLYTGEIRNALLRII
OR.scaffold_142.2 AVIQAVSLSVQAFSYVKILIACMVSR----RSEAK-MKAINTCVAQLMILAIFEFVATFTILSHRFN--TVSADLQKIMGMLIFLIPPLLNPIVYGLYTGEIRNALLRII
OR.scaffold_142.3 ILIQGVPLAIMMYTYAQILYVCIMTN----NADAR-QKAIQTCSSHLVVYLFLQVNTLVTLMAHRNG--KVSPTIRRALGVSILIFPPLLDPIIYGLRVQKLKMGIKMLL
OR.scaffold_142.5P ALTQVIANVTIMYTYLQILVACFGSK----QPDTK-HKALQTCATHLIVFLLLECLGLFTIISYRLK--DISPHLRKFQGMSTLIFPPTLNPIIYGLRTKEIRDK-----
OR.scaffold_3630.2 VNLLIVPVMFILFSYARILLISYQSS----KEVRK--KAAQTCLPHLMILINFSCLTVYDVLLLRLN-TVVPKTVHFLITLQIIMYHPLFNPIVYGFKMKEISKQLKKLF
OR.scaffold_5510.1 VNLLIVPVMFILFSYARILLISYQSS----KEVRK--KAAQTCLPHLMILINFSCLTVYDVLLLRLN-TVVPKTVHFLITLQIIMYHPLFNPIVYGFKMKEISKRLKKLF
OR.scaffold_8617.1 VNLVIVPVMFILFSYARILLISYQSS----KEVRK--KAAQTCLPHLMILINFSCLTVYDVLLLRLN-TVVPKTVHFLITLQIIMYHPLFNPIVYGFKMKEISKQLKKLF
OR.scaffold_5887.1 VNLVIVPVMFILFSYARILLISYQSS----KEVRK--KAAQTCLPHLMILINFSCLTVYDVLLLRLN-TVVPKTVHFLITLQIIMYHPLFNPIVYGFKMKEISKQLKKLF
OR.scaffold_4429.1 FHLVVLSLVLILFSYIRILLVSYQSS----KEVRR--KAAQTCLPHLMILINFSCLCSYDVILAKAK-IHVSRTTQLIMTFQVIMYHPLFNPFVYGLKMKEISKHLSAHF
OR.scaffold_4429.2 MVSGIIPMFFILFTYVKIIQVSYQSC----KEVRT--KSAQTCLPHLLVLINFSLLSAYDIIIVRLN-ARIPKAVHLFMTLQFVVYQPLFNAIIYGLKMREISSSLRKLF
OR.scaffold_7743.1 ACVIALPCLLVFLSYFHIFSVTLKVS----TQSQK--KALQTCTPHLVAFINFSAASFFGVIYNRMS-DAVPETVNIFTCVSFFIFPPLLNPIIYGIKMKEIRLSIQKIG
OR.scaffold_117.1 VTTVFLPLAFVLYTYGRIFFICRKCS----SDFKS--KVVHSCLPHIITFVNYSVTVFCDVALSRIDLESLNPFIAVVLSLEFVVIPPLVNPLVYGLKLPEIRKCILRMF
OR.scaffold_2346.5 ILTIIFPLLYVLYTYIQIVATCWKGS----AGFNR--RVFQSCVPHIISFVINSLTVFCDIALSRNNIEEINPFLAVIFSLEFVVIPPVLNPLVYGLKLEQIRREILRLF
OR.scaffold_2346.4 VLLMTVSSGFILYTYARIVIACWRKT----LNVRG--KVVQSCLPHLISFMVYIITSFSDTVLSRQNVEEINPFLAVILSIGFVIIPPALNPFIYGLKLPEIRRQIIRIL
OR.scaffold_2346.6 ITFYFCHFLYVIYSYFYIMKTCLTCK----EERM---KFLQTCLPHLMSCIIVALSVLFDVLNMRLSLEKLPDSAQNFIAMQFLLFPPLINPLIYGLKLTKVRNRIEGFL
OR.scaffold_3763.1 ITFYFCHFLFIIYSYFYIMRTCLTCK----EERI---KFLQTCLPHLMSCIIVTMCLLFDLLHIRISTERLPDSAQNFIAMQFLLFPPLINPLIYGLKLTKVRNRIERFL
OR.scaffold_3763.2 ITFYFCHFLYIIYSYFYIMKTCLTCK----EERM---KFMQTCLPHLSSAIVVIGCLLFDLLHMRYDSEKLSESARNFMAIQFLLFPPLINPLIYGLKLTPIRNRIQSFL
OR.scaffold_4133.1 ITFYFCHFLYVIYSYFYIMKTCLTRK----EERM---KFMQTCLPHLSSLIIAKICLLFDLLHMRFDSGQLSEGARNFMAIQFLLFPPLINPLIYGIKLTPIRNRIQSFL
OR.scaffold_6828.1 ITFYFCHFLLVTYSYFYIMRTCLTGK----EERM---KFLQTCLPHLMSFIIVAMCLLFDVLHVRLRSEKMSESAQNFIAIQFLLFPPLINPLIYGLKLTKVRNRIERFL
OR.scaffold_6765.1 ITFYSCHVLVIYSYFYIMKTCLTSK----EDRR---KFIQTCLPHVSSLIVVIVCLLFDLVHMRFDSGTLSDNARNFMAIQFLLFPPLINPLIYGIKLTPIRNRIQNFL
OR.scaffold_123.1 YIFYFCHFSAVVWSYVYLIQKCLGSK----ESRT---KFMQTCLPHLLCLLTMVICMLFDLLYMRFGTKNLPESIQNFIAIQFILIPPILNPLIYGFKLQKIRRRIQYFL
OR.scaffold_117.2 ILLYFGHFVFIIWSYMYMIRSCLSSK----EDQH---KFMQTCLPHLISLIMFGVSVLLDLMYMRFGNVDVSPHFNNFMSIEFLLIPPLVNPLIYGFKLTHIRNRIVSVM
OR.scaffold_123.2 TLIYVFHGFFIIWTYIHLAITCTKSK----EDRV---KFVQTCIPHLVSLIIFLFVIVFDLMFTRFGSNNLPNSVINLIGIKFVIFPPLVNPIIYGFKLQKVRCKIAGYM
OR.scaffold_2346.2 ILFYFCHDVFIMCSYVPLLKSAVKSR----DGRK---KFTQTCVPHLFCLLNVTVALLFDLMYARYGSPSLSAGVKNFMALQFLLFPPILNPLIYGLKLTQVRNRLLNVF
OR.scaffold_2346.3 IVFYFCHALLIASSYVQLVKATLKST----EGRK---KFMQTCLPHLCCLFNVTASLLFDLMYSRYGSVSLPQHLRNFMAIQFLIITPVLNPIIYGLKLTNIRNTMRGYI
OR.scaffold_5287.1 MFSHLLQTALILVSYTHLVRASLKLQ----SDRR---KFMQTCVPHLLTLLVFTASLMFDIMYSRYGSGSTLQVLQNALAAEFLVVPPVINPIIYGINLHQIRSKIIHRF
OR.scaffold_2346.1 FLTIVVPFGLISFSYVKILGVCLNAS----KENTQ--KAVSTCTPQIVSLSNMFLGTLFHFSESRIDAAAVPDKLRIILSIYLLLIQPMITPFLYGFNLPKMRQSCKRFL
OR.scaffold_7149.1 FLTIVVPFSLISFSYVKIMGVCLNAS----KENTQ--KAVSTCTPQIVSLSNMFLGTLFHFSESRIDAAVIPDKLRIILSIYLLLIQPMITPFLYGFNLPKIRQSCKRFL
OR.scaffold_5511.2 VVSVVVPLVPILFSYMKILQVCFSGC----RQTRQ--KAVSTCTPHLVSLLNFSFGCCFEILQSRFDMSSVPRMLRAILSLYFLLIQPLLNPLMYGLQMSQIRKLYKHLF
OR.scaffold_119.1 LVLILPQLLMIIFSYVQISQVCRNLT----RESQR--RALKTCVPHLFSLLNYTIGSIFEISQTRFSMTHMVAEARIFMSLYFVILPSISNPVLYGLGTQLIRVNLLKLL
OR.scaffold_5287.2 ICFVGLDIPLILFSYIKILSVVSRAS----VGSEDRWKAFHTCGTHLMVMMCFYLVGSVTFLSHNLN-INIPADVNTFMGVMYILFPATVNPIIYGVRTTEIRNGLLKIF
OR.scaffold_142.6 LLCLLLPLTFIVFSYVTIIVVIAKMS----STAGR-RKTLSTCTPQLFVTCLFYLPRCFVYVANTV-GFYFSIDVRILLTLLYSLVPPAVNPFIYCFKTQDIKKILMKKL
OR.scaffold_142.7 MFSLLVPLSCIILSYLLIIAVVIRMS----DSSSR-IRTLSTCTPQLLITCLYYMPRCFVYLANNL-GFTFSVPIRIVVVMMYSLIPPAAINPLIYCFKTKDIKESLKKKI
OR.scaffold_3154.1P NILLXIPLLFILASYCCIFCALFKLS----TANER-VKALKTCTGHLSLVALFFLPIIIVYQL----QEALHPNIRILNLSLNYVIPPTLNPFIYALQTQEIKNSLKQLL
OR.scaffold_3905.1 VFIFWLPLLFILLSYLYISCTLAKVA----TVQQG-LKAFRTCIGHLLLVAIYFIPLLITFTL----MENIHPNARIINLSLTSVFPPMLNPIIYVLQTREIKDSLKRLL
OR.scaffold_4479.1 VVNLVAPFAFILLTYAAILVTLFLMK----SVNSR-MKALSTCVEHLVLVAIFYIPLFTIFLGFY-VRVIEADQRVLSLSLASCIPPCVNPIIYSLKTREIKIRAVALI
OR.scaffold_4479.2 VVNLVAPFAFILLTYAAILVTLFLMK----SVNSR-MKALGTCVEHLVLVAIFYIPLFIIFLGFY-VRVIDADQRVLSLSLASCIPPCVNPIIYSLKTREIKIRAVALI
OR.scaffold_6269.1 VVNLVVPFTFILLTYAAILVTLFLMK----SVNSR-MKALGTCVEHLVLVAIFYIPLFTIFILGFY-VRLIDADQRVLSLSLASCIPPCVNPIVYSLKTREIKIRAVALI
OR.scaffold_4208.2 ALFLVIIWITIFYTYFQILLMAKTAG----KDTVKARNTIILHSFQLLLCMASYLAPALRELLQKLFP-KNSTDILFVSYIVVQVLPRSTSPMIYGLRDPTFRKYLKRYM
OR.scaffold_4208.3 VVYLVIVWLIIFYTYFRIMFTAKTAS----KDARKARNTIILHGFQVLLCMATYAEPLLKQALLWFP-RNYSDSLFACYIIVQILPRSISPIIYGVRDKTFRRHLTRNL
OR.scaffold_4208.1 TVFLVLVWLTLVYTYINIVCAAKRAS----AQAQRARNTIILHAFQVLLSMLNYVRVLFEQGLLLLP-GHYMSIIFASYVIIQILPRFVSPIVYGLRDQTFRKYVSRYL
OR.scaffold_11306.1 IIYFSLVFLTLVFSYLKILFAARALSTE-KTSALRARNTILLHGVQLFMCMLSYISPSVEIALHLIFP-GWILEIRFANYLIVYILPRFLSPIIYGFRDKKFRKYLKMNL
OR.scaffold_59.1 CLLFVSAGVAISFSYIGVTVAVRLASTD-KTSSQKARNTLLLHLVQLGLSLSSTVYKPILAALSRIVTRLVLIRVQNVLYVCFFILPRCLSALIYGIRDQLIRPVLFYYL
OR.scaffold_6030.1 CLLFVSAGLAIAFSYIGVTVAARLASTD-KTSSQKARNTLLLHLVQLGLSLSSTVYKPILAALSRIVSRIVLVRLQNVLYVCLFILPRCLSALIYGIRDQLIRPVLFYYL
OR.scaffold_144.1P CLLFVSAGVAIAFSYIGVTLAARLASTN-KTSAQKARNTLLLHLVRLGLXLSSTVYKPVLMALSRIVTRIVLIRVQIVLYVWFILPRCLSALIYGIRDQTIRPVLFYHL
OR.scaffold_7573.1 CFLFVSAGLAIAFSYIGVTVAARSASTD-KTSAQKARNTLLLHLVQLGLSLSSTVYKPVLTALSRIVTRIVLIRVQIVLYVWFILPRCLSALIYGIRDQLIRPVLFYHL
OR.scaffold_6392.1 QFYFFIMSITITFSYVKIMIVAKAASGENKKSTWKGLSTVILHAFQLLLCLIQLWCPFIESAIIKIDL-LYINVRYFDYVTFILAPRCLSPLIYGLRDEKFLNALKYLA
OR.scaffold_7903.1 QFYFFIMSITITFSYVKIMKVAKAASGENKKSTWKGLSTVILHAFQLLLCLIQLWCPFIESAIIKIDL-LYINVRYFNYVTFILAPRCLSPLIYGLRDEKFLNALKYLA
OR.scaffold_26.3P QFYFFIMSITITFSYVKIMKVAKAASGENKKSTWKGLSTVILHAFQLLLCLIQLWCPFIESAIIKIDL-LYINVRYFNYVTFILAPRCLSPLIYGLRDEKFLNALKYLA
OR.scaffold_6818.1 QFYFLIMCVVILLSYIKVMKVAKAASGENKKSTWKGLRTVILHAFQLLLSLIQLWCPFIESAIFQIDY-MLFINVRYFNYVTFILAPRCLSPLIYGLRDEKFFNALKYLA
OR.scaffold_217.1 IAYFVIVSVVIIYTYVAILVIVKSASSR-SHNATKAGKTVLLHLIQLCLCLVSTLFNMINYNSKWNMQHIVVTHIQYTIFISLIIFPKCLSPLIYGLRDQAIRQILQSYI
OR.scaffold_26.4 ILFFAVVAVIILFTYMRILLETRKLRQD-RLSVNKAMHTVLLHGFQLLLSVLAFTSPITETLIVLHAN-WLPEDISFFNYFCFILIPRFLSPLIYGFRDQTLRSHIGKAF

Figure S2

```
                             340       350       360       370       380       390       400       410
                        ....|....|....|....|....|....|....|....|....|....|....|....|....|....|....|....|....|
OR.scaffold_1026.1      FHQKVLQSK----------------------------------------------------------------------------
OR.scaffold_8298.1      FHQKVFAVKINYLVAPLVH------------------------------------------------------------------
OR.scaffold_1026.2      QARKIMSSY----------------------------------------------------------------------------
OR.scaffold_4563.2      MHMKSLSFS----------------------------------------------------------------------------
OR.scaffold_4563.1      TRNSKTASIIQH-------------------------------------------------------------------------
OR.scaffold_142.1       KKRSISV------------------------------------------------------------------------------
OR.scaffold_142.2       KKRSISV------------------------------------------------------------------------------
OR.scaffold_142.3       KRNVFLK------------------------------------------------------------------------------
OR.scaffold_142.5P      -------------------------------------------------------------------------------------
OR.scaffold_3630.2      GKKGLF-------------------------------------------------------------------------------
OR.scaffold_5510.1      GKKRFVLTCVYTLHHK---------------------------------------------------------------------
OR.scaffold_8617.1      GKKGLF-------------------------------------------------------------------------------
OR.scaffold_5887.1      GKKRFVLTCVYILHHK---------------------------------------------------------------------
OR.scaffold_4429.1      HLEPQLNASV---------------------------------------------------------------------------
OR.scaffold_4429.2      C------------------------------------------------------------------------------------
OR.scaffold_7743.1      QRILQQP------------------------------------------------------------------------------
OR.scaffold_117.1       HNPK---------------------------------------------------------------------------------
OR.scaffold_2346.5      TRHNIII------------------------------------------------------------------------------
OR.scaffold_2346.4      RLKH---------------------------------------------------------------------------------
OR.scaffold_2346.6      CRKS---------------------------------------------------------------------------------
OR.scaffold_3763.1      CRKR---------------------------------------------------------------------------------
OR.scaffold_3763.2      CRKSM--------------------------------------------------------------------------------
OR.scaffold_4133.1      CMKSL--------------------------------------------------------------------------------
OR.scaffold_6828.1      CRKS---------------------------------------------------------------------------------
OR.scaffold_6765.1      GKEI---------------------------------------------------------------------------------
OR.scaffold_123.1       WTKHVCI------------------------------------------------------------------------------
OR.scaffold_117.2       HMKRKVALQSKK-------------------------------------------------------------------------
OR.scaffold_123.2       SRK----------------------------------------------------------------------------------
OR.scaffold_2346.2      KGKG---------------------------------------------------------------------------------
OR.scaffold_2346.3      CR-----------------------------------------------------------------------------------
OR.scaffold_5287.1      SHKAEH-------------------------------------------------------------------------------
OR.scaffold_2346.1      LGHKINIFM----------------------------------------------------------------------------
OR.scaffold_7149.1      LGHKINIFM----------------------------------------------------------------------------
OR.scaffold_5511.2      S------------------------------------------------------------------------------------
OR.scaffold_119.1       MRHKIPLTKLSKTVRQS--------------------------------------------------------------------
OR.scaffold_5287.2      KLQVKKLIKVSPV------------------------------------------------------------------------
OR.scaffold_142.6       RNTGFGMKVNIYS------------------------------------------------------------------------
OR.scaffold_142.7       FSRISTNLKPG--------------------------------------------------------------------------
OR.scaffold_3154.1P     KFRVQTKIV-RKL------------------------------------------------------------------------
OR.scaffold_3905.1      KLGRKSKTTPRKLPNLPQTAACKC-------------------------------------------------------------
OR.scaffold_4479.1      RKNKIKVIKF---------------------------------------------------------------------------
OR.scaffold_4479.2      RKNKIKVMKF---------------------------------------------------------------------------
OR.scaffold_6269.1      RKSKIKGTKF---------------------------------------------------------------------------
OR.scaffold_4208.2      LARSS--------------------------------------------------------------------------------
OR.scaffold_4208.3      LCRPPRAPEEPAQT-----------------------------------------------------------------------
OR.scaffold_4208.1      LCKMGLGGH----------------------------------------------------------------------------
OR.scaffold_11306.1     QIRRCGVPRPEVAPVNTL-------------------------------------------------------------------
OR.scaffold_59.1        CCRLTLSVFPAKGYK----------------------------------------------------------------------
OR.scaffold_6030.1      CCRLTLSVFPSKGYK----------------------------------------------------------------------
OR.scaffold_144.1P      CCQLKLQSFCLKATNRPFLQTLICMYFYICLLQCFCGDINPLINENKWSTIDPIMRPTEFSHHNEIVFGFYFV------------
OR.scaffold_7573.1      CCRLNYQSFRVKATNRPFLQTLIYMYYHTCFLRCLCGVRELRVAAVAYFCLSYPEISFVTRGNIFPFSEIKQTVMLCLSQGQGQ-
OR.scaffold_6392.1      LCGLYKKKIGFT-------------------------------------------------------------------------
OR.scaffold_7903.1      LCGLYKKKIDYX-------------------------------------------------------------------------
OR.scaffold_26.3P       LXXXXXXXXXXXXXXXXXDFLPLSLICVRATFX----------------------------------------------------
OR.scaffold_6818.1      LCGLYKKRLDLFDE-----------------------------------------------------------------------
OR.scaffold_217.1       PFRFTAAARTFASS-----------------------------------------------------------------------
OR.scaffold_26.4        LCCSNRGRPCVRRKQCDRVFAF---------------------------------------------------------------
```

Figure S2
