# Supplementary material for: The odorant receptor repertoire of teleost fish
Source: BMC Genomics. 2005 Dec 6;6:173. doi: 10.1186/1471-2164-6-173 (PMC1325023; doi:10.1186/1471-2164-6-173)
Supplement: Additional File 4 — Figure S3. Multiple sequence alignment of tetraodon OR amino acid translations. [file 1471-2164-6-173-S4.pdf]

```
                                  ⇓ TM1                                  TM2
                        10        20        30        40        50        60        70        80        90       100       110
                ....|....|....|....|....|....|....|....|....|....|....|....|....|....|....|....|....|....|....|....|....|....|
OR.SCAF12348.1    ---------MDNTS-ALRTFTLSGINETMN-YRITIFSLTLLYYCVVLFVNITLIVIIIHDENLHEPMYVFLCSFCINGLYGATGFYPRFLFDLLSSS--HEISYEGCLL
OR.SCAF7360.1P    ---------MDNTS-ALRTFTLSGINETMN-YRITIFSLTLLYYCVVLFVNITLIVIIIRDENLHEPMYVFLCSFCINGLYGATGFYPRFLFDLLSSS--HEISYEGCLL
OR.SCAF11981.1    ------------------------------------------------------------------XFLCSFCINGLYGATGFYPRFLFDLLSSS--HEISYEGCLL
OR.SCAF8981.2     ---------MNNTS-VVTMFTLSGFDSITE-HRFILFALTFVFYCVIVQVNVTLIVIIIIDKSLHEPMFIFICNLCINALYRTAGFYPKFLMDILSDS--HVISYAGCFV
OR.SCAF8981.3     ---------MNNTS-VVTMFTLSGFDSITE-HRFTLFALTFVCYCVIVQVNVTLILIIIIDKSLHEPMFIFLCNLCINALYGTAGFYPKFLMDILSTS--HVISYAGCLV
OR.SCAF13663.2P   ---------MDNAS-EVTILTLSGFNSIVN-FRVTLFVVKCLYYCVIKKANVTLIVAIIVDKSLHEPMFIFIXNLCINALYGTAGFYPKFLMDILTDS--HVITYVGCLT
OR.SCAF7360.2P    ---------MDNVS-RVNMFTLAGFGEITN-YRFPLFVLTLLHYCVIWMANMTIILTILLDKKFHEPMYIFLCNLCINSLYATAGFYPKILSDILTAS--RVISYVGCFL
OR.SCAF14677.3    ---------MWNGS-DV-LLVLQGLNASRG-QRQVYFGLALSAYLLTILINLLLVLTVWLHRSLHKPIYIFLCNLCLNGMCGASSFYPKLLRDLLAEA--QVVTHGGCLA
OR.SCAF12097.1    ---------MENSS-ASFYFNLTMFVNIGH-YRYPAFVFCLLLYSFMISANLVIILVVSRERTLHEPMYIFVALLSVNSLYGSTGFFPRFLSDLLVDV--HLMSRPECFT
OR.SCAF12097.3    ---------MENSS-ASFYFNLTMFVNIGH-YRYPAFVFCLLLYSFMISANLVIILVVSRERTLHEPMYIFVALLSVNSLYGSTGFFPRFLSDLLVDV--HLMSRPECFT
OR.SCAF12097.2    ---------MENSS-XSFYFNLTMFVNIGH-YRYPAFVFCLLLYSFMISANLVIILVVSRERTLHEPMYIFVALLSVNSLYGSTGFFPRFLSDLLVDV--HLMSRPECFT
OR.SCAF12097.4P   ---------MENSS-XSFYFNLTMFVNIGH-YRYPAFVFCLLLYSFMISANLVIILVVSRERTLHEPMYIFVALLSVNSLYGSTGFFPRFLSDLLVDV--HLMSRPECFT
OR.SCAF14321.1    ---------MDNST-NPPYFNLTMFFNIGQ-SYNLAFVLFFILYLSIVFANVIIIVVTARENALHEPMYIFIMCLAVNSLYGSAGFFLRFLRDLLSSS--HLISRPECFV
OR.SCAF14370.1    ---------MDNST-NPPYFNLTMFFNIGQ-SYNLAFVLFFILYLSIVFANVIIIVVTARENALHEPMYIFIMCLAVNSLYGSAGFFLRFLRDLLSSS--HLISRPECFV
OR.SCAF14328.1    ---------MDNST-NPPYFNLTMFFNIGQ-SRYLAFVLFFILYLFIVFANVIIIVVTARENALHEPMYIFIMCLAVNSLYGSAGFFLRFLRDLLSSS--HLISRPECFV
OR.SCAF14339.1    ---------MDNST-NPPYFNLTMFFNIGQ-SRYLAFVLFFILYLFIVFANVIIIVVTARENALHEPMYIFIMCLAVNSLYGSAGFFFRFLRDLLSSS--HLISRSECFT
OR.SCAF14339.2    ---------MDNST-NPPYFNLTMFFNIGQ-SRYLAFVLFFILYLFIVFANIIIIVVTARENTLHEPMYIFIMCLAVNSLYGSAGFFLRFLRDLLSSS--HLFSRSECFT
OR.SCAF14534.1P   ---------MDNST-NPPYFNLTMFFNIGQ-SRYLAFVLFFILYLFIVFANVIIIVVTARENALHEPMYIFIMCLAVNSLYGSAGFFLRFLRDLLSSS--HLISRPECFV
OR.SCAF14328.2    ------------------------------------------------------------XKLISKPMYIFIMCLAVNSLYGSAGFFLRFLRDLLSSS--HLISRSECFT
OR.SCAF14703.1    ---------MDN-T-TVLTFTLTAYAVMKN--KSGFFTLFLLLYLLIITLNLILLTLIQQTKKLHQPMNVFTFLLCLNELYGSTAFLPPTMSLLLSKT--YEIPVKHCLA
OR.SCAF14677.2    --------MMVNSTLSSLPIVLEAYLALGR-WKYLGFVLLLLLYVLTVASNLLLTAVICLSRSLHQPMYVLLCSLFVNELYGSTALVPFLLLQILSDL--HAVSAPFCFL
OR.SCAF3073.1     --------MMVNSTLSSLPIVLEAYLALGR-WKYLGFVLLLLLYVLTVASNLLLTAVICLSRSLHQPMYVLLCSLFVNELYGSTALVPFLLLQILSDL--HAVSAPFCFL
OR.SCAF14677.4P   ---------MQNAS-AVFSFILGAYLPLGN-LRYLYFVLTALLYAVVVVLNTSLVAVICLSRSLHQPMYVLLCSLFVNELYGSTALFPFLLLQILSDL--HAVSAPFCFL
OR.SCAF14677.6    ---------MQNAS-AVFSFILGAYLPLGN-LRYLYFVLTALLYAVVVVLNTSLVAVICLSRSLHQPMYVLLCSLFVNELYGSTALFPFLLLQILSDL--HAVSAPFCFL
OR.SCAF14677.5P   ----------MNSS-QVSYFLLGAYENLGA-YSYLLFTLVLLFYLLIVASNLLLTAVICLNRSLHQPMYVLLCSLFVNQLYGSTALFPFLLLQILSDL--HAVSATFCFL
OR.SCAF14677.7    ----------MNSS-QVSYFLLGAYENLGA-YSYLLFTLVLLFYLLIVASNLLLTAVICLNRSLHQPMYVLLCSLFVNQLYGSTALFPFLLLQILSDL--HAVSASFCFL
OR.SCAF8332.1     ---------MENRS-ETEYFVLAAYGDVGE-FKYLYFSIMLLWFVSIWVVNVVLILVIYIDKQLHEPMYIFLCNLSVTEISGSTSLYPLMLSQMLSDT--HRVTVPFCFL
OR.SCAF15134.2    ---------MEP-RANVSYITVDGYVEVHR-YRYLYFVLMLTAYLLILCTNSTVVYLICLHRDLHQPMYVLIAALLLNSVIYSSTIYPKLLLDFLSEE--QTITYSACLF
OR.SCAF15134.3    ---------MEP-RANVSYITVDGYVEVHR-YRYLYFVLMLTAYLLILCTNSTVVYLICLHRDLHQPMYVLIAALLLNSVIYSSTIYPKLLLDFLSEE--QTITYSACLF
OR.SCAF15134.1    ---------MEP-RANVSYITVDGYVEVHR-YRYLYFVLMLTAYLLILCTNSTVVYLICLHRDLHQPMYVLIAALLLNSVIYSSTIYPKLLQDFLSEE--QTITYSACLF
OR.SCAF14677.1    ---------MN---SSTVLLTFTIYSSLGS-FSLELAVCVLLLYVSGLCVNLLLVLVICADARLHRPMYVLLVHLTLSGLLGSSTVTLSLVRHLLSQA--ES-SLSACLS
OR.SCAF11408.5    ---------MENYTYNSFMLQMEGLKVQ-KNHTNLVFFMLFMSYIFGLLVNAGILVLIFIDKNLHQPMYLIFCNLLVSDIMGSSNVIPRLMLDLLRPPSERLITYYECVV
OR.SCAF11408.3    ---------MENFSYNSFILQVEGLTLE-EESVYPVFFLFLFCYLFIIVANVGITALIFIDSSLHQPMYLLFCNLSCNDILGNSILIPRLLLDLLRPPSERLITYYECVV
OR.SCAF15002.3P   --------------------------------------------------------------------------------XIGITAMLLKMLSDIATET--NRVHYGLCVL
OR.SCAF15002.4    ---------MDGLYNVSSVLELRRFELP-PEAVLPAFLFAALSYMIILFCNLVLISVIALNKSLHQPMYLILLNLPVNDLIGSSALFLQVLRELLTKS--RTMHYSSCVT
OR.SCAF15002.2P   -------MSSNISFSALTLLHLEKLNLS-HIYIYPTLIFVTLLYVVMILCNMMVLLAIAWCKELHQPMFILLFSLPISDMIGATAFFPQLLWSIATQN--SSIPYAACFI
OR.SCAF15002.1    -------MSSNISFSALTLLHLEKLNLSSHIYMYLMLIFVTLLYVVMILCNMMVLLAIAWCKELHQPMFILLFSLPISDMIGATAFFPQLLWSIATQN--SSIPYAACFI
OR.SCAF14601.1    MGLFNAAPGKNISFVHPPYFIISGLMGIP--HIRFYYIFLFFVYIVSVLGNSSVMAVICLDRSLRTPKYVAVFNLAFVDLFGNSALVPKLLDIFLFDH--RRIPYNDCLT
OR.SCAF15001.1    -MLFLQTVSNDTVIIHPPGFYVIGFETFP--HIRFYFIFLAFVYVVTLLFNCLLIYVIASHPCLHTPKFLAVVNLAVIDIVLSSSTIPSMIRTFLLKE--NFFPFNLCLL
OR.SCAF15002.5    --------MTNN-VTGTNSFVILGFPGLAPRYYGPVSALFLIIYLAIVLGNVFILTFVMCHKSLQKPMYLVFCHLALIDLMFGTVTLPKIISKYWFGN--GDISFHGCFT
OR.SCAF15002.6    --------MTNTTITTVKYFIITGFPGLPPKHHSLVSALLFLLFLAILLGNVLILALIMYNDALWKPKYCILFHLAMTDLLFGFVTLPKVIAVYWWKA--VVSPFAACFT
OR.SCAF14677.8    -----MLDGSPGHNLSHSTFIFRGFPELHR-YRRLLVLPFSATYLLVLLGNSLLVYVICNMEHLHSPMYLLICSLCVVDILAATAIFPSMLLSLLFDM--DKISLASCLT
OR.SCAF10960.3    ----------MNASAANASG-TAAFRDSFSKAVTKNAVVLVLGLFINYINRGLIHTFCKHQVFYTNSRYVLFIHLVINDMLHVNLTMVLFLISY--TVY--RVNVCVCWL
OR.SCAF10960.2    ----------MSAPDANVSV-VVEYRDSLSKAVTKNVIVVVLSISINYVNVGLVQTFHRHQVFYSNPRYILFFHLVVNDMIQVTLTVLLFVISY--VLY--KIEVSVCCV
OR.SCAF10960.6    ------------------X-AAALRDSSASALVKNLLVVLVWLVISGINGSLVATFFRHQELYEDPRYILFIHMVVNDAVQLSTTTALFVLSY--TLY--RIQVSVCCV
OR.SCAF10960.5    ----------MNLSQTNGTAGGSARRDSLLTALLKNLMVTCLCLLINYVNGVQIHTFRKHQVFYSNPRYVLFIHLVVNDMLQVSLSSLLHVLSY--VFL--TVSASFCAV
OR.SCAF10960.4    ----------MNLAWGQNQT--LAAPQALRAG--SNAAVLFLCITINYINAAMVHTFHKHHVFRTSPRFILFIHLVVNDMIQLSISSTLFLLST--TVR--TVSVHVCCL
OR.SCAF14536.1    ----------MDKVLSSSNVTDELQNRGTVERLMFSFLTTVPCCIFFYINAIMLFTLRSKTVFCETSRYVLLFNLLLADTIQMVTSQLLYILAA--CLI--RLTYPVCGF
OR.SCAF14536.4    ----------MDKVLSSSNVTDELQNDRTVERLMLSFLTTVQCCIFFYINAIMLFTLRSKTVFCETSRYVLLFNLLFADSIQMVTSQLLYILAA--CLI--RLTYPVCGF
OR.SCAF14536.2    ----------MDKMRSLSNVTAEQQYQGLLERLMLSFLTTVPCCIFFYINTIMLFTLRSKTVFCETSRYVLLFNLLFADTLQMVLCQVLFILAN--CWI--KMSYPVCGF
OR.SCAF14536.3    ----------MNKNQTLTNLTAEP---WLLEKILSATLITVQCCIFFYINAIMLFTLRSKTVFCETSRYVLLFNLLFADSTQMVLSQVLYILAT--CSL--KMSYPVCGF
OR.SCAF11324.1    --------------------------------------------------------------------------------XLHVVDSSVLYILAV--SLM--RLTYPVCGF
OR.SCAF14536.5P   ----------------MDNVTAEQQYQGLLERLMLSFLTTVPCCIFFYINAIMLFTLRSKTVFCETSRYVLLFNLLFADSTQMVLSQVLYICGYY-VLL--KMSYPVCGF
OR.SCAF14825.1    ----------------MDNVTAEQQYQGLLERLMFSFLTTVPCCIFFYINAIMLFTLRSKTVFCETSRYVLLFNLLFADTIQMVLSQVLYILAA--CLM--RLTYPVCAF
OR.SCAF14448.1    -------------MKNLTENLGNTTFNKSLSVIIKVCVVIPFFCLFLGCIAIMLHIFACHRQFLDSSRYILFAHMLINDTLQVLSSVLLFLFAM--GQV--KLALVYCIP
OR.SCAF14955.1    -------------MKNLTENLGNTTFNKSLSVIIKVCVVIPFFCLFLGCIAIMLHIFACHRQFLDSSRYILFAHMLINDTLQVLSSVLLFLFAM--GQV--KLALVYCIP
OR.SCAF12434.1    -----------------XQILFER-----------VLITQILAFMFISTNLVLLATVFSKPVFYSTMRYILFTITLVSDSITLIISDLLLLNVFSVP----LQVSLCII
```

Figure S3

```
                            TM3                                  TM4                                     TM5
                          120        130        140        150        160        170        180        190        200        210        220
                    ....|....|....|....|....|....|....|....|....|....|....|....|....|....|....|....|....|....|....|....|....|....|....|....|
OR.SCAF12348.1      QAFAVHSFACCEMSILAVMAYDRYVAICQPLRYHSIMTRRR-LAALVGSSLAQCPFVSFPLGIFVTSTLKLCG-SKLQKLFCVNVMIYELACSKSDTLANNIIAHATIQI
OR.SCAF7360.1P      QAFVMYSFACCEMSILAVMAYDRYVAICQPLRYHSIMTRRR-LAALVGSSLAHATCFTFPXVYLXPPRXKLCG-SKLQKLFCVNVMIYELACSKSDTLANNIIAHATIQI
OR.SCAF11981.1      QAFVMYSFACCEMSILAVMAYDRYVAICQPLRYHSIMTRRR-LAALVGSSWL-VPFFIFSLGVSVTSTLKLCG-SKLQKLFCVNWMIYELACSKSDTLANNIISHLTTLF
OR.SCAF8981.2       QSLVVTSSTCVDFSFLVLMAYDRYVAICRPLVYHSVMNTQR-VSVLVFAAWF-VPVVQMLVIIISTSTIRLCS-SKISRVYCVTYAKRRLECSVS--ITTVLFPAFMITF
OR.SCAF8981.3       QSLVVTSSTCNDFFFLVIMGYDRYVAICRPLVYHSVMNTQR-VSVLVFAAWF-VSVGQMIITIIITSTLRLCG-SEISRIYCVNYAIRRLECSAS--ITATVLPAFMIAI
OR.SCAF13663.2P     QSLVVHSYVCADFTLLVLMAYDRYVAIXRPLVYHYXXXILRGFLSCVFAAWF-VPVGQIFIIIISTSSLKLCS-SKISRLYCVTYAKRRLECSVT--ITTILFPAFMMTF
OR.SCAF7360.2P      QSYVLYSSICAEFSLLVLMAYDRYVAICRPLVYHSVMTSRR-VCLLVFIAWL-VPLYMMFMSTIALSRNKLCG-SHIPKIYCINDFIDKLSCSAS--VTYPLIXGIIYIS
OR.SCAF14677.3      QLFGIYCYVFCEFTSLTVMAYDRYLAICRPLQYGSVMTVQK-VLQLLLTWS-FSLLETAVGTFLTARLPLCR-HRLPKIFCTNWEVVKLSCLGANTTVNSIYGLILTIS
OR.SCAF12097.1      QIYVFYTYVSFEMTILGIIAYDRHVAVCSPLHYHTKMTRKT--VVLLAASACILPLFTIALGLFLTVRLPLCG-NKIQRVLCGNWNIVKLSCVSTI-GNNIYGIFLT-VT
OR.SCAF12097.3      QIYVFYTYVSFEMTILGIIAYDRHVAVCSPLHYHTKMTRKT--VVLLAASACILPLFTIALGLFLTVRLPLCG-NKIQRVLCGNWNIVKLSCVSTI-GNNIYGIFLT-VT
OR.SCAF12097.2      QIYVFYTYISFEMTILGIIAYDRHVAVCSPLHYHTKMTRKT--VVLLAASACILPLFTIALGLFLTVRLPLCG-NKIQRVFCGNWNIVKLSCVSTI-VNNIYGMFLTIVT
OR.SCAF12097.4P     QIYVFYTYVSFEMTILGIIAYDRHVAVCSPLHYHTKMTRKT--VVLLAASACILPLFTIALGLFLTVRLPLCD-NKIQRVFCGNWNVVKLSCVSTN-VNNIYGMFMT-TF
OR.SCAF14321.1      QIYVIYTYGSFELSFLSVMAYDRYVAVCQPLHYHNKITKKT--VILLIGLAVTIPAIIVTPFVYLSSTLPLCG-NAIPKVFCANWLVVKLSCVPTG-LSDLFGMLVTTPV
OR.SCAF14370.1      QIYVIYTYGSFELSFLSVMAYDRYVAVCQPLHYHNKITKKT--VILLIGLAVTIPAIIVTPFVYLSSTLPLCG-NAIPKVFCANWLVVKLSCVPTG-LSDLFGMLVTTPV
OR.SCAF14328.1      QIYVIYTYGSFELSFLSVMAYDRYVAVCQPLHYHNKITKKT--VILLIGLAVTIPAIIVTPFVYLSSTLPLCG-NAIPKVFCANWLVVKLSCVPTG-LSDLFGMLVTTPV
OR.SCAF14339.1      QIYVIYTYGSFELSFLSVMAYDRYVAVCQPLHYHNKIRTRA--VILLIALAVTIPAISVTPFVYLSSTLPLCG-NAIPKVFCANWYVVKLSCVPTG-LNDLVGMVLTTPV
OR.SCAF14339.2      QIYVIYTYASYELTFLSVMAYDRYVAVCQPLHYHNKITKRT--VILLIGLAVTIPAITVTPFVYLSSILPLCG-NAIPKVFCANWHVVKLSCVSTG-LNNLLGMLVTTPV
OR.SCAF14534.1P     QIYVIYTYASYEFTFLSVMAYDRYVAVCQPLHYHNKITKRA--VILLIAVAVTFPVSSVVSCVYLSSTLPLCG-NAIPKVFCANWPVVKLSCVSTG-LNNLVGMLLTTTT
OR.SCAF14328.2      QIYVIYTYASYELTFLSVMAYDRYVAVCQPLHYHNKITKRT--VILLIAVAVTFPVSSVASCVYLSAVLPLCG-NAIPKVFCANWPVVKLSCVSTG-LNNLVGMLLTTTT
OR.SCAF14703.1      QVYVLHSYASLEFSFLALMAYDRYLAICYPLHYHGIMSHSR--MGKIIALAVAWPNIIFLCYYSITLQLTFCG-RFIPKLYCVNMEVVKNSCSMP-AYISIVGLLLILVL
OR.SCAF14677.2      QIFCVYTYVNVEFCTLAVMAYDRFVAICQPLQYHLRMSLSR--TLLLVFVLWLYSFLKCLSTLALSLQLQRCG-VAINSLYCHNYLVVKLSCSDTR-VNNIYGLLGIVVS
OR.SCAF3073.1       QIFCVYTYVNVEFCTLAVMAYDRFVAICQPLQYHLRMSLSR--TLLLVFVLWLYSFLKCLSTLALSLQLQRCG-VAINSLYCHNYLVVKLSCSDTR-VNNIYGLLGIVVS
OR.SCAF14677.4P     QIFCLYSYAQVQFCSLAVMAYDRYLAICQPLQYQQLMTCTR--ASVWVAALWVYSFVKFLITLWLNIRLPLCG-NLINSLYCHNYLVVKLSCSDTR-VNNIYGLVGTVLT
OR.SCAF14677.6      QIFCLYSYAHVQFCSLAVMAYDRYLAICQPLQYQQLMTCTR--ASVWVAALWVYSFVKFLITLWLNIRLPLCG-NLINSLYCHNYLVVKLSCSDTR-VNNIYGLVGTVLT
OR.SCAF14677.5P     QIFCVHSYGGVEFMTLAAMAYDRYAAICQPLQYQRLMSSSR--LGLLLAAAWWYPSVFAVVLLWLRSPLQLCG-NTIHKVYCGSHAVVKLSCSGST-ASNAYGLVATFSV
OR.SCAF14677.7      QIFCVHSYGGVEFMTLAAMAYDRYAAICQPLQYQRLMSSSR--LALLLAAAWWYPSVFAVVLLWLRSPLQLCG-NTIHKVYCGSHAVVKLSCSGST-AVNAYRLVATFSV
OR.SCAF8332.1       QMSYIYTSASVEFCSLAAMAYDRYVAICHPLQYSLIMSTGR--VCKIILAVWGYSFFTFLVLLLFLIHLDLCG-NVINKVFCDYYRIIQLACSVSL-IEKMSDMYFAFLT
OR.SCAF15134.2      QCFAFYSLAGSEFLLLAVMAYDRYVAICTPLRYPAVMRRRR--VAALLLLAWVAPAFQTAVPTITNANKKLCH-FVFKGMICNSTAY-DLLCQRSQ-TLNIYGLIVLVNL
OR.SCAF15134.3      QCFAFYSLAGSEFLLLAVMAYDRYVAICTPLRYPAVMRRRR--VAALLLLAWVAPAFQTAVPTITNANKKLCH-FVFKGMICNSTAY-DLLCQRSQ-TLNIYGLIVLVNL
OR.SCAF15134.1      QYFAFYSLAGSEFLLLAVMAYDRYVAICTPLRYPAVMRRRR--VAALLLLAWVAPSILVVVPTITNANKKLCH-FVFKGIICNSTIY-DLLCQRSQ-ILNIYGLIVLVNL
OR.SCAF14677.1      QVFFTNVYGSSMFCLLALMAYDRYVSICKPLLYHAIMRPGR--LRLLLALLYLLLSSSSAVQVYLTSTLPLCQ-HSLDKLLCDSLAISKLSCQRNS-LVGLYGLCCAACV
OR.SCAF11408.5      QAFTTQLYGTTSHTVLMIMAFDRYVAICNPLRYAAIMSPRM--VVKLTLFAWGSAFVLVGVLLGLTIRLNRCR-TLIRNPFCDNASLFKLSCES-VAINNIYGLTFSAVL
OR.SCAF11408.3      QAFISHMFGTTSHTVLMIMAFDRYVAICNPLRYASIMSPRM--VVKLTLFAWGSAFVLVGVLLGLTIRLNRCR-TLITNPFCDNASLFKLSCES-VAINNIYGLTFTALL
OR.SCAF15002.3P     QAFFLHMYGGGILFILAAMSFDRYVAICMPLRYNSIMVPRA--VVCIIFLVWGVDLSLIMLLFILQIRLPRCK-SVIMNVFCDNPSLLKLTCGN-TTVNNVIGLFNTAII
OR.SCAF15002.4      QAFFIHIYAGGTVFILTAMAYDRYIAICNPLKYNTIMTHAH--VFRIITCLWLTNLAILSVLFFLLLRLPRCR-SELTHPYCDNPSLLALMCAD-VTVNHIYGLFTVAIT
OR.SCAF15002.2P     QAYLLHVYGTGNLVILSVMAYDRYIAICFPLRYHEILSPLT--LVKMILSVWVITLSVIGSVFLLHKQYKICR-TNIVDFYCNNPSLLKLMCGE-TNVSNYYGLVSIVLV
OR.SCAF15002.1      QAYLIHVYGTGNLMILSVMAYDRYIAICFPLRYHEILSPLT--LVKMILSVWVITLSVMGSVFLLHKQYKICR-TNIVDFYCNNPSLLKLMCGE-TNVSNYYGLVFIVLV
OR.SCAF14601.1      FLFFCYTCLSMQALNLVALAYDRLVAITYPLHYQVRVTHRF--MISMIASFWAFIIVAVLIAVGLITRLSFCKSVVVHSYFCDHGQIYRLACNDHFPNYVVSCLYP-VFI
OR.SCAF15001.1      QMFTYYSFATLESLALAILAYDRLIAICFPLQHNSINTLWS--MSWIVAVTWCYSLGLAAFSASIMTWLSFCKSVEVYSYFCDYAPTFRLACNDNTLHWAVSSTAS-VLN
OR.SCAF15002.5      QMFFVHYLGSVTSFILLVMALDRFISICLPLRYTALVSTKF--ISALCGIAWFFPVSLMVTIIVQALALTFCEPKLIVQCFCDHISITNQACGEGVQSVRVSTLCIALFC
OR.SCAF15002.6      QMYFVHSLGAVHSLILLMMSLDCFVAIWFPFRYAVVVTNKT--IIIACNLCWILTFIRMMGIVLHALTLPYCNLNIIPQCYCDHVSVMQLACGENVEYVKKVAFGNSMVT
OR.SCAF14677.8      QMFFTHFLSSLESTLLLAMALDRYVAICQPLRYARIINFSM--LVRLLVFTFVRSSSIMATLVGLASSLTFCGSNTIQHCYCDHMALVSLACDS-TEKNSAAGLAVIICF
OR.SCAF10960.3      LLLPALITTENTPLNLAAMALECYVAVCLPLRHAQICTVRR--TRTLILLMWTTTVCSSASDLLLTLATERLA-IVRSQVFCLRHTAFPHPVIV------KKRDATYSLF
OR.SCAF10960.2      FILLALFTTENTPLNLAAMALECYVAVCRPLRHAQICTVRR--TLLLIALIWTTSCVSILPDLLVTLATQPLG-FFRSRVFCLRETAFPNPHII------RKRDVTYIAY
OR.SCAF10960.6      FILLAVFTTRNTPLNLAAMALECYVAVCRPLRHAQICTARR--TLLLIALIWLLCVTPDVTDLFFTLATEPLS-FFHQSVFCLRSNIFKGPVLA------RKRTAFDVLY
OR.SCAF10960.5      ALLATMLTTQNTPLNLAAMAVERYLAVCLPLRYAHICTLRR--TYRLVGAMWAAGLLAVLPDLFFLAATRPRR-FFGSRLVCS-MEVFGEARGA------MRKDAWHAAF
OR.SCAF10960.4      LVVPAVATTQNTPLNLSLMATECCLAVCAPLRYAGVCSVLR--TRLSIALMWSVSLLSVLPDVVLLWAAEPPD-FRRSRILCNRDTLFRRAESQ------SRRDVSHILL
OR.SCAF14536.1      LNVIGVLTSDISPLTLVVMSLERYVAVCFPLRHAAVITTRN--TGLAIFAIWAFSFFSSLIRVSLVDFSELEN-LQM-KEFCSSLTVFVGPLSD------TYDRASTCLL
OR.SCAF14536.4      LNVIGVLTTNISPLTLVVMSLERYVAVCFPLRHAAVITTRN--TGLAIFAIWAFSFFSSLIQISLVDFPELEN-LQM-KEVCSGLTVFVGPLSD------TYDRAFTCLL
OR.SCAF14536.2      LNVIGVLTSDISPLTLVVMSLERYVAVCFPLRHAAVITTRN--TGLAIFAIWAFSLFSSLIRISLVDFSELEN-LQM-KEFCSGLTVFVGPLSD------TYDRASTCLL
OR.SCAF14536.3      LNVIGVLTTNISPLTLVVMSLERYVAVCFPLRHAAVITTRN--TGLAIFAIWAFSLLSVFVRVGLVDFPELGS-LQM-KEVCSVFNVFVGPLSD------TYDRAFTCLL
OR.SCAF11324.1      LNVIGVPTSNISPLTLVVMSLERYVAVCFPLRHAAVITVRN--TGLAIFAIWALSLLSVFIRVGLVDFSELGS-LQM-KEVCSVFTFFVGSLSD------TYGRASTCLL
OR.SCAF14536.5P     LNVIGVLTTNISPLTLVVMSLERYVAVCFPLRHAAVITTRN--TGLAIFAIWAFSLLSVFIRVGLVDFSELGS-LQM-KEFCSSLTVFVGPLSD------TYGRASTCLL
OR.SCAF14825.1      LNVIGAFTNTISPLTLVVMSLERYVAVCFPLRHAAVITTRN--TGLAIFAILGFQFAKYFCSSWFGRFFRTEE-PAD-ERNLHSFNVFVGPLSD------TYNKASTCLL
OR.SCAF14448.1      LLFLSTTTFQNTPLILATMSLERYVAIFYPLQRPAAWRSDR--IWAIILSLWLISSAFPIISYSAGDRDPGVK-VLYTPVLCKDYTVNSSPVQV------LFRATVSILF
OR.SCAF14955.1      LLFLSTTTFQNTPLILATMSLERYVAIFYPLQRPAAWRSDR--IWAIILSLWLISSAFPIISYSAGERDPGVK-VLYTPVLCKDYTVNSSPVQV------LFRATVSILF
OR.SCAF12434.1      CYLIVLFCGFITPVTLTAMTLERYVAICLPLQHPRLCSLHN--TWNCMLIINALSCVPCFIILSTFFAAAPSS-VYKQYKLCSMEMFVAVQWQK------QLKFSVYQSY
```

Figure S3

```
                    TM5                          TM6                      TM7         ⇓
                            230        240        250        260        270        280        290        300        310        320        330
                   ....|....|....|....|....|....|....|....|....|....|....|....|....|....|....|....|....|....|....|....|....|....|....|....|....|....|
OR.SCAF12348.1     YVLHGFFIIWTYVHLAITCTN-SKE--D-RVKFIQTCIPHLVSLIVFLFV-IVFDLMFMRFG---SKTLPQSLMNLIGIKFVIFPPLVNPIIYGFKLQKIRHRIAGYVSR
OR.SCAF7360.1P     YVLHGFFIVWTYVHLAITCTN-SKE--D-RVKFIQTCIPHLVSLIVFIFV-VMFDVMFMRFG---SKTLPQSLMNLIGIKFVIFPPLVNPIIYGFKLQKIRHRVAGYV--
OR.SCAF11981.1     YVLHGFFIVWTYVHLAITCTN-SKE--D-RVKFIQTCIPHLVSLIIFLFV-IVFDLMFMRFG---SKTLPQSLTNLIGIKFVIFPPLVNPIIYGFKLQKIRGKIAGFVRK
OR.SCAF8981.2      YLCLFLLVIYSYFHIMKTCLT-SKD--D-RIKFLQTCLPHLMSFIVMSMC-LLFDILHEGFS---SERIPESARNFIAIQFLLVPPLINPLIYGLKLNKVRNRINRFMCR
OR.SCAF8981.3      YFCVFLLVIYSYFHIMKTCLS-SKD--D-RIKFLQTCLPHLMSFIVVNMC-LLFDMLYEKLS---TEKIPKSTQNFIAIEFLFFPPLINPLIYGLKLNKVRNRIEKFLCR
OR.SCAF13663.2P    YFCLFLLVIHSYFHIMRTCLT-SKE--D-RIKFLQTCLPHLMSLIVVTMC-LLFDVLYEQLS---SERIPESAQNFIAIQFLLFPPLINPIIYGVKLSKVRNRIERFLFR
OR.SCAF7360.2P     NVRQFIVVIWSYVHLIRKCLV-SQK--S-RAKFMQTCLPHALCLITVATY-LLFDLLYMRFG---STQLPQIFQNFIAIQFVLVPPIVNPLIYGFNLKQIRKRIVQVLFC
OR.SCAF14677.3     HLSQTTLILVSYTHLVRASMK-LRS--N-RRKFMQTCVPHLLALLVFTIS-LLFDIMYSRYGGSSSSSTLAVLQNALAAEFLVVPPIINPIIYGINLHQIRTKILQHLRP
OR.SCAF12097.1     IFFPLLYVLYTYVQIVAVCWKGAAE--F-RRKVFQNCLPHVISFVLNSLT-IVCDVAFSRYN---IEEINPFLAVILSLEFILIPPVLNPLVYGLKLPQIRREIFRLFAK
OR.SCAF12097.3     IFFPLLYVLYTYVQIVAVCWKGAAE--F-RRKVFQNCLPHVISFVLNSLT-IVCDVAFSRYN---IEEINPFLAVILSLEFILIPPVLNPLVYGLKLPQIRREIFRLFAK
OR.SCAF12097.2     IIFPLLYVLYTYVQIVAVCWKGAAE--F-RRKVFQNCLPHVVSFVLNSLT-IFCDVALSRYN---IEAINPFLAVILSLEFIVLPPVLNPLVYGLKLPQIRREIFGLFAK
OR.SCAF12097.4P    IIFPLLYVLYTYVQIVAACRKGAAE--F-RRKVFQNCLPHVISFFLNSST-VFCDIALSRYN---IEKINPFLAVILSLEFIVIPPVLNPLVYGLKLPQIRREIVRLFRK
OR.SCAF14321.1     VFLPFVFVLYTYGRIFLICRKRTSD--F-KRKVVQSCLPHIITFVNYSIN-FFCDVALSRID---LESLNPYIAVILSLEFVVIPPLVNPLVYGLKLPEIRKCILRMFQN
OR.SCAF14370.1     VFLPFVFVLYTYGRIFLICRKRTSD--F-KRKVVQSCLPHIITFVNYSIN-FFCDVALSRID---LESLNPYIAVILSLEFVVIPPLVNPLVYGLKLPEIRKCILRMFQN
OR.SCAF14328.1     VFLPFVFVLYTYGRIFLICRKRSSD--F-KRKVVQSCLPHIITFVNYSIN-FFCDVALSRID---LESLNPYIAVILSLEFVVIPPLVNPLVYGLKLPEIRKCILRMFQN
OR.SCAF14339.1     IFLPFVFVLYTYGRIFLICRNRSSD--F-KRKVMQSCLPHIITFVNYSIN-FFCDIALSRID---LESLNPYIAVILSLEFVVIPPLVNPLVYGLKLPEIRKCILRMFQN
OR.SCAF14339.2     VFLPFVFVLYTYGRIFLICRNRSSD--F-KRKVVQSCLPHIITFVIYSIT-VFCNVALSRID---LESLNPYIAVILSLEFVVIPPLVNPLVYGLKLPEIRKCVLRMFQN
OR.SCAF14534.1P    IFLPLLFVLYTYGRIFLICRKRSSD--F-KTKVMESCLPHIITFVNYSIT-AFCDVALSRID---LESLNPYIAVILSLEFVVIPX------------------------
OR.SCAF14328.2     AFLPLVFVLYTYGRIFLICRKRSSN--F-KRKVVQSCLPHIITFVNYSIT-AFCDVALSRID---LESLNPYIAVILSLEFVVIPPLVNPLVYGLKLPEIRKCILRMFQS
OR.SCAF14703.1     ILPQLLMVLFSYVQISRVCRKLPKE--S-RCNALKTCIPHLLSLMIYTIG-SIFEIIQTRFN---MNHVVAEARIFMSLFFVIIPSLFNPVVYGLGTQLIRVYLLKRLMG
OR.SCAF14677.2     VLVPLVLILFSYLRILQVCFSGSRQ--T-RQKAVSTCSPQLVSLLNFALT-CLLEIVQTRLS---LAGLPRMLRNVLSLYFYLLQPLMTPVMFGLQVSAIRSRCKAVLWP
OR.SCAF3073.1      VLVPLVLILFSYLRILQVCFSGSRQ--T-RQKAVSTCSPQLVSLLNFALT-CLLEIVQTRLS---LAGLPRMLRNVLSLYFYLLQPLMTPVMFGLQVSAIRSRCKAVLWP
OR.SCAF14677.4P    VLVPLVPILFSYLRILQVCFSGSRQ--T-RQKAVSTCSPHLISLLNFSFSWLLFASLQSRFD---MTSVPSMLQMFLSLYFLLIQPVLNPLLYGLQMTQIRKFYKRLLSC
OR.SCAF14677.6     VLVPLVPILFSYLRILQVCFSGSRQ--T-RQKAVSTCSPHLISLLNFSFG-CCFAILQSRFD---MTSVPSMLQMFLSLYFLLIQPVLNPLLYGLQMTQIRKFYKRLLSC
OR.SCAF14677.5P    VVGCLLVIGYSYLRILQVCSSGSRR--T-RQKAVSTCSPHLASLLNFSIG-VAFETVQSRLD---MQHLPTLLRVVLSLYFLTCPPLLNPVIYGLKMSKLQRLVSKAGR-
OR.SCAF14677.7     VVGCLLVIGYSYLRILQVCSSGSRR--T-RQKAVSTCSPHLASLLNFSIG-VAFETVQSRLD---MQHLPNLLRVVLSLYFLTCPPLLNPVIYGLKMSKLQRLVSKAGR-
OR.SCAF8332.1      VIVPFSLILFSYLKILAVCLKASKE--N-TQKAVSTVTPQIVSVSNLFVG-CTFHFLESRTD---VTFLPVNIVIFFSVYLLIFQPMINPFMYGFSLPKIKQSFKRLMFN
OR.SCAF15134.2     VILPVVFILFSYIRILLVSYQSSRE--V-RRKAAQTCLPHLLILINFSSF-TIYDVLLIRLS----TTVPKTVRFLITLQMIMYQPLFNPIIYGLKMREISKHLKKLFAR
OR.SCAF15134.3     VILPVVFILFSYIRILLVSYQSSRE--V-RRKAAQTCLPHLLILINFSSF-TIYDVLLIRLS----TTVPKTVRFLITLQMIMYQPLFNPIIYGLKMREISKHLKKLFAR
OR.SCAF15134.1     VILPVIFILFSYIRILLVSYQSSRE--V-RRKAAQTCLPHLLILINFSSF-GIYDVLLLRLN----TTVPKTVRFLITLQMIMYQPLFNPIIYGLKMREISKHLKKLFAR
OR.SCAF14677.1     IALPCLLVLLSYCHIFSVMLKLSGE--S-QRKALQTCTPHLVVFINFSGA-AFCGVTYNRLS----ANIPKTVNILACVSFFLVPPLFNPIIYGIKMKEIRLSIHRVMRR
OR.SCAF11408.5     LCSSIGSVVLTYTKITIVCVTSNSK-SV-NSKALKTCSTHLTVYLIMLIS-GCIVILMHR------FPQYAEYRKLCGILFFVIPGSLNTIIYGVQSKEIQKFLRKSTLL
OR.SCAF11408.3     LCSSIGSIVLTYTKITIVCVTSNSK-SV-NSKALKTCSTHLTVYLVLILT-GVSNIVLHRS-~~~-YPEYRYYRLFCMIMLHIIPGILNPIVYGLHSKEIRQLLSKPCVH
OR.SCAF15002.3P    QIISLTIQAFSYVKILIACRVSCRS-ET-KLKAINTCVAQLMILVIFEFV-ATFTILSHRF-~~~-KNVSADLQKIMGMLIFLIPPLLNPIVYGLYTSEIRNTFLRIFKK
OR.SCAF15002.4     QVVANVIIMYTYLQILVACFRSKHP-DT-KSKALQTCATHLIVFLLLECL-GLFTIISYRL~~---KDISPHFGKFMGMSTLIFPPTLNPIIYGLKTKEIREKLEAFCRN
OR.SCAF15002.2P    QGAPLAMMVYTYAQILYVCVSTKST-DA-RKKAIQTCSSHLLVYILLRKY~DYRVFIDLFIH~~~~GVMGX----------------------------------------
OR.SCAF15002.1     QGAPLAMMVYTYAQILYVCVSTKST-DA-RQKAIQTCSSHLLVYILLQVN-TLFTLLAHRN~~---PNVHPSIRRAFGLSVLVFPPLLDPIIYGLRVKKLKKGLKML---
OR.SCAF14601.1     FWLPLLFILLSYLYISCTLAKVANF-QR-GLKALKTCTGHLLLVAIYFIP-LLITFTLM--~~~~-ENIHPNARIINLSLTSVFPPMLNPIIYVLQTQEIKDSLRRLLKN
OR.SCAF15001.1     LVAPFVFIVLTYTAILLTLFRMKSV-NS-RMKALSTCVEHLVLVAVFYIP-LFTIFIFGFYV----RLIDADLRVLSLSMASCIPPCINPIVYSLKTKEIKIRAVALFRK
OR.SCAF15002.5     LLLPLAFIVFSYVSIIVVIMRRSNG-AG-RRKTLSTCTPQLFITCLFYLP-RCFVYVANTVG----FYFSVDVRILLVLLYSLVPPAVNPFIYCFKTQDIKQTLIKKLRN
OR.SCAF15002.6     LLVPLSFIIFSYSSIIVAVLKISQN-DR-RYKVLSTCIPQFTVTCLYYVP-RCFVYIATDLG----FELSGETRTIVTTMYSLMPAMLDPIIYCLKTKDIKETLMKNIRI
OR.SCAF14677.8     VGMDIPLILFSYAKILSVVSRASVASED-RWKAFHTCGTHLMVMMCFYLV-GSVTFLSRNLN----IYIPADVNTFMGVMYILFPATVNPIIYGVRTTEIRNGFFKLCKI
OR.SCAF10960.3     LVMVWITMFYAYFRILVAAKTASKD----AKKARNTIVLHSFQLLLCMAA-YLTPALRDALQRRFPE-NSVDVLFASYVLVQILPRSLSPLVYSVRDQTFRRYLRRYLLR
OR.SCAF10960.2     LLAVWLVIFYTYFRILFTATSASGD----SRKARRTIVLHGVQVLLCMAT-YAEPLLKQALQLWFPR-SYSDSLFACYIIVQILPRSISPIIYGVRDQTFRRYLRRNLLC
OR.SCAF10960.6     FCCVFLTLLFTYLKVLFAARALSTQRTS-AQKARKTILLHGAQLFMCLLS-YVSPSAEVLLQLIFPG-QILEIRFANFLIVYVLPRCLSPIIYGFRDRKFRKHLKMSLQL
OR.SCAF10960.5     LVLVWLTLLYTYVSIGCAARRASAQ----AQKARNTILLHAFQLLLSMLS-YVRGPFEQALLRLLPR-RYLAIVFASFILIQVLPRLVSPVVYGLRDQSFRKYVGRYLLC
OR.SCAF10960.4     LGLVWSTLVYTYLRILLVAKAADGA----AKKARSTILLHAFQMLLSMLV-YVQPMVLQGAGTMAPA-RLLLLHFLIFILNQMLPRFLSPFIYGFRDKTFWRYLKKNLCG
OR.SCAF14536.1     FVSAGATILFSYIGVTVAAKSASTNKTT-SQKARHTLLLHLVQLGLSLSS-TMYTPILTALSRTVIRIVLVRVQIVLYVWFFILPRCLSALIYGIRDRTIRPVFFYCLCC
OR.SCAF14536.4     FVSAGAAIIFSYIGVTVAAKSASTDKTT-SQKARHTLLLHLVQLGLSLSS-TLYTPILTALSRTVIRIVLVRVQIVLYVWFFILPRCLSALIYGIRDRTIRPVFFYCLCC
OR.SCAF14536.2     FVSAGAAIIFSYIGVTVAAKSASTDKTT-SQKARHTLLLHLVQLGLSLSS-TLYTPILTALSRTVIRIVLVRVQIVLYVWFFILPRCLSALVYGIRDRTIRPVFFYCLCC
OR.SCAF14536.3     FVSAGAAIIFSYIGVTVAAKSASTDKTT-SQKARHTLLLHLVQLGLSLSS-TLYTPILTALSRTVIRIVLVRVQIVLYVWFFILPRCLSALVYGIRDRTIRPIFFYYLCC
OR.SCAF11324.1     FVSAGAAIIFSFIGVTVAAKSASTDKTT-SQKARHTLLLHLVQLGLSLSS-TLFTPILTVLSRLMMRIVFVRTQNVLYVCFFILPRCLSALIYGIRDRTMRSVLFYYLCC
OR.SCAF14536.5P    FVSAGAAIIFSYIGVTVAAKSASTDKTT-SQKARHTLLLHLVQLGLSLSS-TAVHTNTDSIIKTNDEDCVCSCTKFLYVCFFILPRCLSALIYGIRDRTIRSVLFYYLCC
OR.SCAF14825.1     FVSAGAAIIFSYIGVTVAAKSASTDKTT-SQKARHTLLLHLVQLGLSLSS-TLSTPVFIALSTLVMSIALFRAQNVLYVCFFILPRCLSALIYGIRDRTIRPVFLFHLCC
OR.SCAF14448.1     FAVVAVVILFTYMRILLETRKLRQDRFS-VNRAMHTVLLHGFQLLLSVLS-FTSP-ITETLTVLHPNWLREDIAFFNYFCFILIPRFLSPLIYGFRDKTLRSYIGKAFLC
OR.SCAF14955.1     FAVVAVVILFTYMRILLETRKLRQDRFS-VNRAMHTVLLHGFQLLLSVLS-FTSP-ITETLTVLHPNWLREDIAFFNYFCFILIPRFLSPLIYGFRDKTLRSYIGKAFLC
OR.SCAF12434.1     FVIMLITITLSYVKIMKIAKAASEETKTSTFKGLKTVILHSFQLLLCLIQLWCPFIESVVFEN--DKVIFSYVRFFNYVVFYIAPRCLSPLIYGLRDEKIFLALKVRVLF
```

Figure S3

```
                            340       350       360
                        ....|....|....|....|....|....|..
OR.SCAF12348.1          K-------------------------------
OR.SCAF7360.1P          --------------------------------
OR.SCAF11981.1          K-------------------------------
OR.SCAF8981.2           KS------------------------------
OR.SCAF8981.3           KS------------------------------
OR.SCAF13663.2P         KIXVCFQ-------------------------
OR.SCAF7360.2P          KRF-----------------------------
OR.SCAF14677.3          REEPQKPPARS---------------------
OR.SCAF12097.1          HKLGRLS-------------------------
OR.SCAF12097.3          HKLGRLS-------------------------
OR.SCAF12097.2          HKLGRLS-------------------------
OR.SCAF12097.4P         QKILTQN-------------------------
OR.SCAF14321.1          PEKKV---------------------------
OR.SCAF14370.1          PEKKV---------------------------
OR.SCAF14328.1          PEKKV---------------------------
OR.SCAF14339.1          PEKKV---------------------------
OR.SCAF14339.2          PEK-----------------------------
OR.SCAF14534.1P         --------------------------------
OR.SCAF14328.2          PEK-----------------------------
OR.SCAF14703.1          NKVLSERKAKTLRQPKSTG-------------
OR.SCAF14677.2          EKLPRRFSK-----------------------
OR.SCAF3073.1           EKLPRRFSK-----------------------
OR.SCAF14677.4P         EG------------------------------
OR.SCAF14677.6          EG------------------------------
OR.SCAF14677.5P         --------------------------------
OR.SCAF14677.7          --------------------------------
OR.SCAF8332.1           NK------------------------------
OR.SCAF15134.2          KVLG----------------------------
OR.SCAF15134.3          KVLG----------------------------
OR.SCAF15134.1          KVLG----------------------------
OR.SCAF14677.1          GILQHR--------------------------
OR.SCAF11408.5          NKKSPFVIF-----------------------
OR.SCAF11408.3          IKRVSFP-------------------------
OR.SCAF15002.3P         RRVSV---------------------------
OR.SCAF15002.4          KFFKSLSGQ-----------------------
OR.SCAF15002.2P         --------------------------------
OR.SCAF15002.1          --------------------------------
OR.SCAF14601.1          SQSLKWAQRKHPILPQPSGSRC----------
OR.SCAF15001.1          HKIRAIKA------------------------
OR.SCAF15002.5          TRIGTEVNVSF---------------------
OR.SCAF15002.6          GKIHVFK-------------------------
OR.SCAF14677.8          RVKKLTKVSPVGKRQT----------------
OR.SCAF10960.3          WRR-----------------------------
OR.SCAF10960.2          RAQRAPERPGDGGPKPAACFCLD---------
OR.SCAF10960.6          LRCRVQ------GPKEAP--------------
OR.SCAF10960.5          RRSPA---------------------------
OR.SCAF10960.4          TRSQQGAPPPGRQAKARK--------------
OR.SCAF14536.1          QLTISVFPAKGYK-------------------
OR.SCAF14536.4          RLTISVFPAKGYK-------------------
OR.SCAF14536.2          QLTISVFPAKGYK-------------------
OR.SCAF14536.3          QCNISVFLAKGYK-------------------
OR.SCAF11324.1          RLSHSVFPNKGYK-------------------
OR.SCAF14536.5P         QLTHSIFPNKGDKQSFFSNSNRKK--------
OR.SCAF14825.1          RLTISILPAKGYK-------------------
OR.SCAF14448.1          CSNRVGPGGRCKP-------------------
OR.SCAF14955.1          CSNRVGPGGRCKP-------------------
OR.SCAF12434.1          -LRSW---------------------------
```

Figure S3
