# Supplementary figures and images for: The odorant receptor repertoire of teleost fish
Source: BMC Genomics. 2005 Dec 6;6:173. doi: 10.1186/1471-2164-6-173 (PMC1325023; doi:10.1186/1471-2164-6-173)

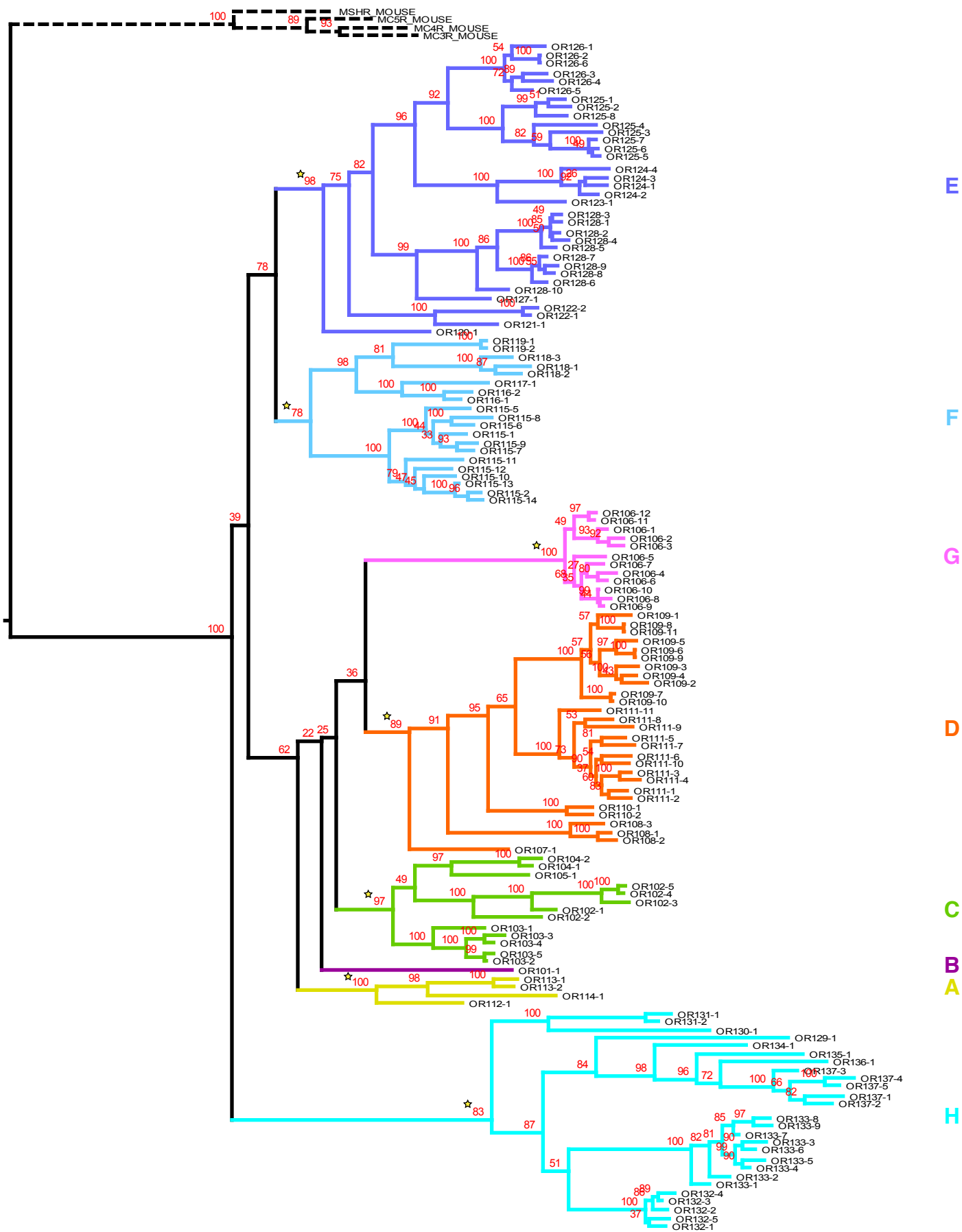

Figure S4

Supplement: Additional File 5 — Figure S4. Phylogeny of zebrafish ORs using maximum likelihood analysis. [file 1471-2164-6-173-S5.pdf]

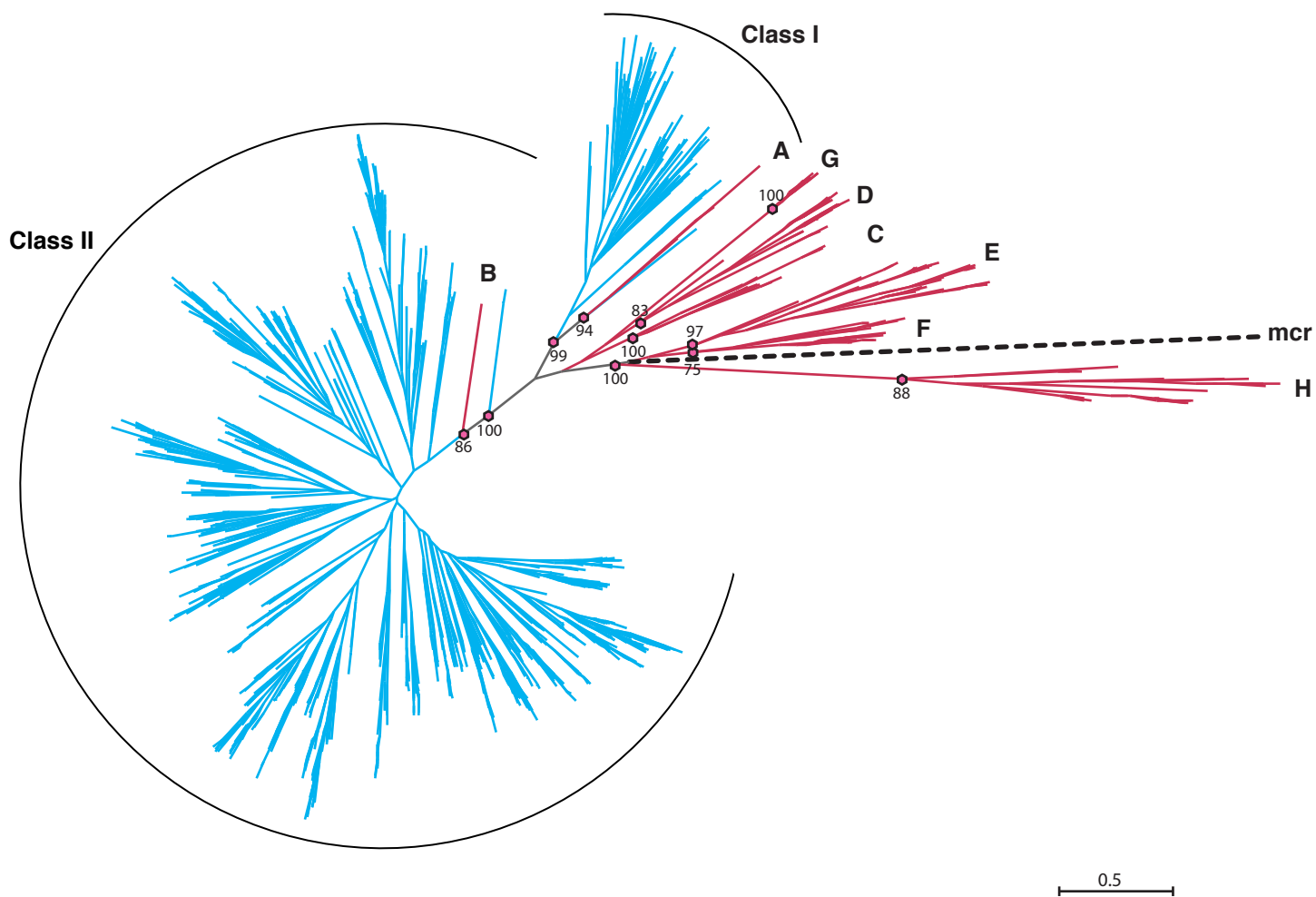

Figure S5

Supplement: Additional File 6 — Figure S5. Phylogeny of zebrafish and mouse ORs using maximum likelihood analysis. [file 1471-2164-6-173-S6.pdf]

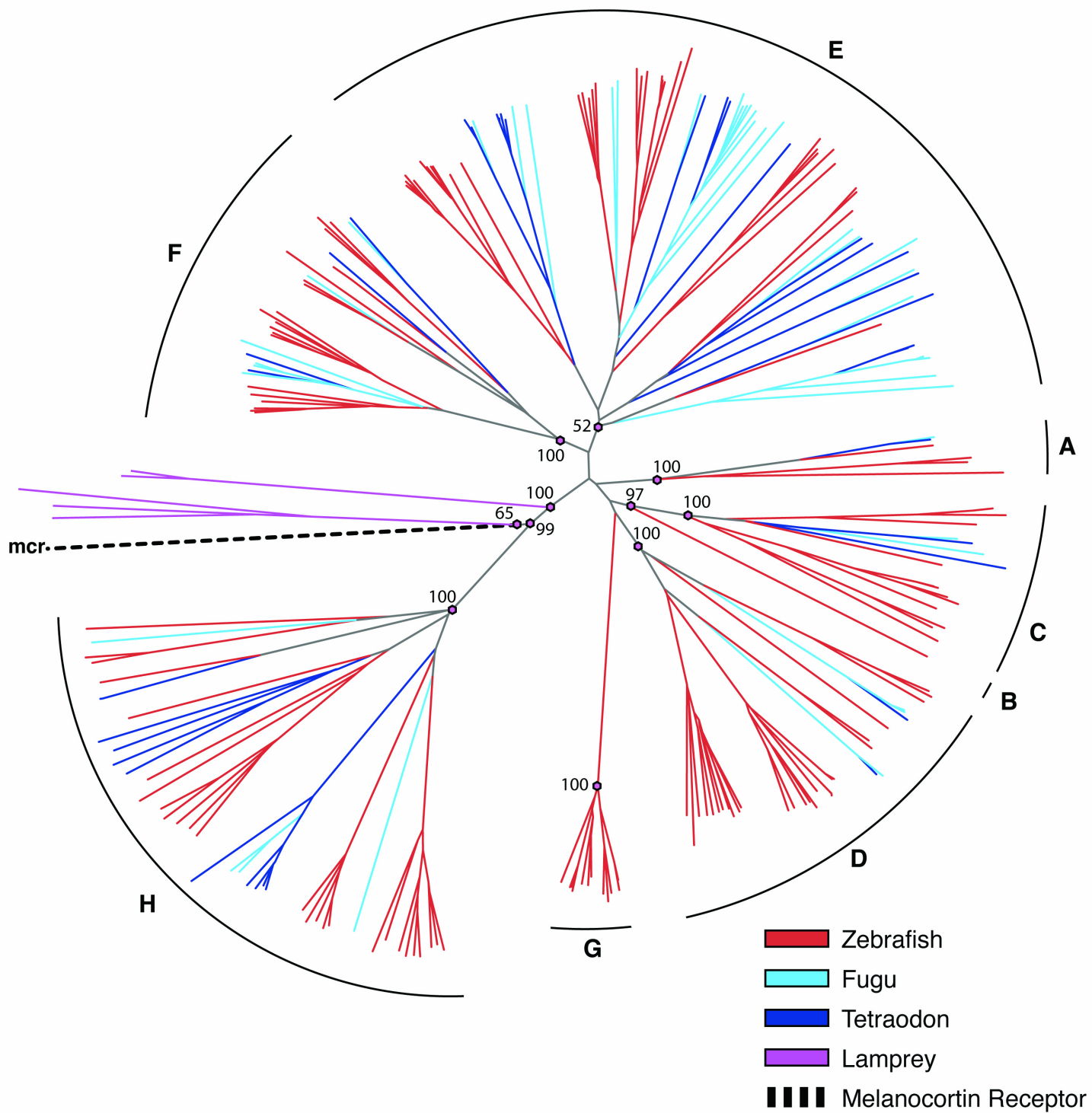

Figure S8

Supplement: Additional File 9 — Figure S8. Phylogeny of zebrafish, fugu, tetraodon and lamprey ORs. [file 1471-2164-6-173-S9.pdf]
