# Supplementary material for: The odorant receptor repertoire of teleost fish
Source: BMC Genomics. 2005 Dec 6;6:173. doi: 10.1186/1471-2164-6-173 (PMC1325023; doi:10.1186/1471-2164-6-173)
Supplement: Additional File 10 — Table S1. The zebrafish OR repertoire. [file 1471-2164-6-173-S10.pdf]

Table S1. The zebrafish OR repertoire

OR Gene	New Accession	Previous Name(s)	Previous Name (f)	Previous Accession(s)	EST Accession	Classification	Gene/Pseudogene	Disruptions	Length (bp)	Annotated Location (Zv3/Zv4/BAC)	Zv4 Location	Chromosomal Location
OR101-1	DQ306041	ZOR1[d]	Dr3OR5.4	U72683		full-length	gene	0	951	Zv4_scaffold1780:182071..183021	Zv4_scaffold1780:182071..183021	21:39155728..39156678
OR102-1	DQ306109	5.1[c]<br>ZOR5[d]	Dr3OR15.48	NM_131740<br>U72687	CO805971	full-length	gene	0	948	ctg26:768131..769078	Zv4_scaffold1337:932115..933062	15:27287344..27288291
OR102-2	DQ306110	5.3[c]	Dr3OR15.49	NM_131739		full-length	gene	0	984	ctg26:772174..773157	Zv4_scaffold1337:936158..937141	15:27291387..27292370
OR102-3	DQ306111	5.4[c]	Dr3OR15.50	NM_131739		full-length	gene	0	987	ctg26:777452..778438	Zv4_scaffold1337:941436..942422	15:27296665..27297651
OR102-4	DQ306112		Dr3OR15.51			full-length	gene	0	981	ctg26:781993..782973	Zv4_scaffold1337:945977..946957	15:27301206..27302186
OR102-5	DQ306114	5.2[c]	Dr3OR15.53	NM_131737		full-length	gene	0	954	ctg26:787119..788072	Zv4_scaffold1337:951103..952056	15:27306332..27307285
OR102-6P	DQ306113		Dr3OR15.52			partial	pseudogene	6	950	ctg26:784842..785791	Zv4_scaffold1337:948826..949775	15:27304055..27305004
OR103-1	DQ306104	13.1[a]<br>ZOR8[d]	Dr3OR15.43	NM_131588<br>U72690		full-length	gene	0	987	ctg26:731961..732947	Zv4_scaffold1337:895945..896931	15:27251174..27252160
OR103-2	DQ306106	13.2	Dr3OR15.45			full-length	gene	0	945	ctg26:741177..742121	Zv4_scaffold1337:905161..906105	15:27260390..27261334
OR103-3	DQ306107		Dr3OR15.46			full-length	gene	0	963	ctg26:743944..744906	Zv4_scaffold1337:907928..908890	15:27263157..27264119
OR103-4	DQ306108	13.4[ab]	Dr3OR15.47	NM_203469		full-length	gene	0	957	ctg26:749834..750790	Zv4_scaffold1337:913818..914774	15:27269047..27270003
OR103-5	DQ306105		Dr3OR15.44			full-length	gene	0	963	ctg26:734376..735338	Zv4_scaffold1337:898360..899322	15:27253589..27254551
OR104-1	DQ305993		Dr3OR10.1			full-length	gene	0	984	Finished101:84610..85593	Zv4_scaffold917:658328..659311	10:29465068..29466051
OR104-2	DQ305994	ZOR10[d]	Dr3OR10.2	U72692		full-length	gene	0	960	Finished101:90370..91329	Zv4_scaffold917:664088..665047	10:29470828..29471787
OR105-1	DQ305995		Dr3OR10.3			full-length	gene	0	978	Finished101:94592..95569	Zv4_scaffold917:668310..669287	10:29475050..29476027
OR106-1	DQ305996					full-length	gene	0	960	Finished101:104926..105885	Zv4_scaffold917:678644..679603	10:29485384..29486343
OR106-2	DQ305997		Dr3OR10.4			full-length	gene	0	963	Finished101:110782..111744	Zv4_scaffold917:684500..685462	10:29491240..29492202
OR106-3	DQ305998		Dr3OR10.5			full-length	gene	0	963	Finished101:116562..117524	Zv4_scaffold917:690280..691242	10:29497020..29497982
OR106-4	DQ305999		Dr3OR10.7			full-length	gene	0	951	Finished101:125185..126135	Zv4_scaffold917:698903..699853	10:29505643..29506593
OR106-5	DQ306000		Dr3OR10.8			full-length	gene	0	951	Finished101:130449..131399	Zv4_scaffold917:704167..705117	10:29510907..29511857
OR106-6	DQ306001		Dr3OR10.10			full-length	gene	0	981	Finished101:136497..137477	Zv4_scaffold917:710215..711195	10:29516955..29517935
OR106-7	DQ306002		Dr3OR10.11		AW232880	full-length	gene	0	978	Finished101:139926..140903	Zv4_scaffold917:713644..714621	10:29520384..29521361
OR106-8	DQ306003					full-length	gene	0	945	Finished101:144861..145805	Zv4_scaffold917:718579..719523	10:29525319..29526263
OR106-9	DQ306004		Dr3OR10.12			full-length	gene	0	942	Finished101:153947..154888	Zv4_scaffold917:727665..728606	10:29534405..29535346
OR106-10	DQ306005		Dr3OR10.13			full-length	gene	0	942	Finished101:162197..163138	Zv4_scaffold917:735915..736856	10:29542655..29543596
OR106-11	DQ306006		Dr3OR10.14			full-length	gene	0	948	Finished101:167530..168477	Zv4_scaffold917:741248..742195	10:29547988..29548935
OR106-12	DQ305986		Dr3OR10.15			full-length	gene	0	981	BX088524:100734..101714	Zv4_scaffold917:748197..749177	10:29554937..29555917
OR107-1	DQ306092	7.1[c]<br>ZOR9[d]<br>ORZF1A[e]	Dr3OR15.30	NM_131750<br>U72691<br>U44439	CO958265	full-length	gene	0	996	ctg26:649954..650949	Zv4_scaffold1337:813938..814933	15:27169167..27170162
OR108-1	DQ305987					full-length	gene	0	966	BX088524:171490..172455	Zv4_scaffold917:841044..842009	10:29647784..29648749
OR108-2	DQ305988		Dr3OR6.2			full-length	gene	0	981	BX088524:181241..182221	Unknown	Unknown
OR108-3	DQ305989		Dr3OR6.1		CO803957	full-length	gene	0	978	BX088524:187251..188228	Unknown	Unknown
OR109-1	DQ306009		Dr3OR10.18			full-length	gene	0	990	Finished101:192523..193512	Zv4_scaffold917:766241..767230	10:29572981..29573970
OR109-2	DQ306010		Dr3OR10.19			full-length	gene	0	1011	Finished101:196495..197505	Zv4_scaffold917:770213..771223	10:29576953..29577963
OR109-3	DQ306011		Dr3OR10.20			full-length	gene	0	1005	Finished101:201141..202145	Zv4_scaffold917:774859..775863	10:29581599..29582603
OR109-4	DQ306012					full-length	gene	0	1017	Finished101:205650..206666	Zv4_scaffold917:779368..780384	10:29586108..29587124
OR109-5	DQ306013					full-length	gene	0	990	Finished101:211674..212663	Zv4_scaffold917:785392..786381	10:29592132..29593121
OR109-6	DQ306014					full-length	gene	0	978	Finished101:214550..215527	Zv4_scaffold917:788268..789245	10:29595008..29595985
OR109-7	DQ306015		Dr3OR10.21			full-length	gene	0	978	Finished101:217578..218555	Zv4_scaffold917:791296..792273	10:29598036..29599013
OR109-8	DQ306016		Dr3OR10.23			full-length	gene	0	1032	Finished101:222852..223883	Zv4_scaffold917:796570..797601	10:29603310..29604341
OR109-9	DQ306018		Dr3OR10.24			full-length	gene	0	978	Finished101:239981..240958	Zv4_scaffold917:813699..814676	10:29620439..29621416
OR109-10	DQ306019		Dr3OR6.3			full-length	gene	0	978	Finished101:243012..243989	Zv4_scaffold917:816730..817707	10:29623470..29624447
OR109-11	DQ306020		Dr3OR10.27			full-length	gene	0	999	Finished101:248418..249416	Zv4_scaffold917:822136..823134	10:29628876..29629874
OR109-12	DQ306017					partial	gene	0	975	Finished101:231120..232094	Zv4_scaffold917:830406..831380	10:29637146..29638120
OR109-13	DQ306021		Dr3OR10.25			partial	gene	0	975	Finished101:256688..257662	Zv4_scaffold917:830406..831380	10:29637146..29638120
OR110-1	DQ306007	ZOR4[d]	Dr3OR10.16	U72686	CO934527	full-length	gene	0	960	Finished101:180476..181435	Zv4_scaffold917:754194..755153	10:29560934..29561893
OR110-2	DQ306008		Dr3OR10.17		CO810947	full-length	gene	0	957	Finished101:186537..187493	Zv4_scaffold917:760255..761211	10:29566995..29567951
OR111-1	DQ306093	2.6[b]<br>ZF1I[e]	Dr3OR15.31	NM_131593<br>U44440	CN013862<br>CO804187	full-length	gene	0	981<br>981	ctg26:656078..657058<br>ctg26:661665..662645	Zv4_scaffold1337:820062..821042<br>Zv4_scaffold1337:825649..826629	15:27175291..27176271<br>15:27180878..27181858
OR111-2	DQ306094	2.4[b]	Dr3OR15.32	NM_131749		full-length	gene	0	981	ctg26:670643..671623	Zv4_scaffold1337:834627..835607	15:27189856..27190836
OR111-3	DQ306095	2.3[b]	Dr3OR15.34	NM_131748	CO802197	full-length	gene	0	981	ctg26:676414..677394	Zv4_scaffold1337:840398..841378	15:27195627..27196607
OR111-4	DQ306096	2.7[b]	Dr3OR15.35	NM_131747		full-length	gene	0	981	ctg26:687576..688556	Zv4_scaffold1337:851560..852540	15:27206789..27207769
OR111-5	DQ306097	2.2[b]	Dr3OR15.36	NM_131592		full-length	gene	0	978	ctg26:694934..695911	Zv4_scaffold1337:858918..859895	15:27214147..27215124
OR111-6	DQ306098	2.5[b]<br>ZOR7[d]	Dr3OR15.37	NM_131746<br>U72689	CO806965	full-length	gene	0	978	ctg26:705339..706316	Zv4_scaffold1337:1377484..1378461	15:27732713..27733690
OR111-7	DQ306099	2.1[ab]<br>ORZF39[e]	Dr3OR15.38	NM_131582<br>U44441		full-length	gene	0	975	ctg26:710239..711213	Zv4_scaffold1337:1383322..1384296	15:27738551..27739525

Table S1. The zebrafish OR repertoire

OR111-8	DQ306100	2.10[c]	Dr3OR15.39	NM_152985		full-length	gene	0	945	ctg26:713020..713964	Zv4_scaffold1337:1386103..1387047	15:27741332..27742276
OR111-9	DQ306101	2.9[c]	Dr3OR15.40	NM_131745		full-length	gene	0				
OR111-10	DQ306102	2.11[ab]	Dr3OR15.41	NM_131581		full-length	gene	0	981	ctg26:717970..718950	Zv4_scaffold1337:881954..882934	15:27237183..27238163
OR111-11	DQ306103	2.8[c]	Dr3OR15.42	NM_131744	CN019319<br>CN013862	full-length	gene	0	978	ctg26:723719..724696	Zv4_scaffold1337:887703..888680	15:27242932..27243909
OR112-1	DQ306144	ZOR6[d]	Dr3OR9.2	U72688		full-length	gene	0	984	ctg9339:234905..235888	Zv4_NA5952:16287..17270	Unknown
OR113-1	DQ306079					full-length	gene	0	954	ctg25655:380111..381064	Zv4_scaffold1495:340499..341452	17:28570443..28571396
OR113-2	DQ306080					full-length	gene	0	954	ctg25655:393664..394617	Zv4_scaffold1495:353029..353982	17:28582973..28583926
OR113-3	DQ306082					full-length	gene	1	947	ctg25655:412946..413892	Zv4_scaffold1495:371047..371993	17:28600991..28601937
OR113-4	DQ306083					full-length	gene	1	950	ctg25655:417801..418750	Zv4_scaffold1495:375902..376851	17:28605846..28606795
OR114-1	DQ306081					full-length	gene	0	945	ctg25655:403808..404752	Zv4_scaffold1495:362662..363606	17:28592606..28593550
OR115-1	DQ306039	9.4[c]	Dr3OR5.1	NM_131742		full-length	gene	0	927	Zv4_scaffold1780:165321..166247	Zv4_scaffold1780:165321..166247	21:39138978..39139904
OR115-2	DQ306139				CO808125	full-length	gene	0	939	ctg30275:230082..231020	Zv4_NA16841:5230..6168	Unknown
OR115-3P	DQ306040		Dr3OR5.3			full-length	pseudogene	2	931	Zv4_scaffold1780:175166..176096	Zv4_scaffold1780:175166..176096	21:39148823..39149753
OR115-4P	DQ306038					full-length	pseudogene	2	952	Zv4_scaffold1780:162298..163249	Zv4_scaffold1780:162298..163249	21:39135955..39136906
OR115-5	DQ306037					full-length	gene	0	942	Zv4_scaffold1780:155915..156856	Zv4_scaffold1780:155915..156856	21:39129572..39130513
OR115-6	DQ306036				CO800381	full-length	gene	0	948	Zv4_scaffold1780:149818..150765	Zv4_scaffold1780:149818..150765	21:39123475..39124422
OR115-7	DQ306035				CN019933<br>CN024010<br>CO811577	full-length	gene	0	942	Zv4_scaffold1780:136998..137939	Zv4_scaffold1780:136998..137939	21:39110655..39111596
OR115-8	DQ306034				CO814811	full-length	gene	0	945	Zv4_scaffold1780:126444..127388	Zv4_scaffold1780:126444..127388	21:39100101..39101045
OR115-9	DQ306033					full-length	gene	0	945	Zv4_scaffold1780:114021..114965	Zv4_scaffold1780:114021..114965	21:39087678..39088622
OR115-10	DQ306032	9.3[a c]		NM_131743		full-length	gene	0	942	Zv4_scaffold1780:110504..111445	Zv4_scaffold1780:110504..111445	21:39084161..39085102
OR115-11	DQ306031					full-length	gene	0	918	Zv4_scaffold1780:106913..107830	Zv4_scaffold1780:106913..107830	21:39080570..39081487
OR115-12	DQ306030				CN015707<br>CN018705<br>CO925021<br>CO813267<br>CO933258<br>AW233446	full-length	gene	0	939	Zv4_scaffold1780:100073..101011	Zv4_scaffold1780:100073..101011	21:39073730..39074668
OR115-13	DQ306029					full-length	gene	0	939	Zv4_scaffold1780:94044..94982	Zv4_scaffold1780:94044..94982	21:39067701..39068639
OR115-14	DQ306028	9.1[c]		NM_131741		full-length	gene	0	939	Zv4_scaffold1780:89657..90595	Zv4_scaffold1780:89657..90595	21:39063314..39064252
*OR115-15**		9.2[c]		U42394								
OR116-1	DQ306084		Dr3OR15.21			full-length	gene	0	936	ctg26:601804..602739	Zv4_scaffold1337:765788..766723	15:27121017..27121952
OR116-2	DQ306085		Dr3OR15.22			full-length	gene	0	939	ctg26:608663..609601	Zv4_scaffold1337:772647..773585	15:27127876..27128814
OR117-1	DQ306086		Dr3OR15.23			full-length	gene	0	981	ctg26:613175..614155	Zv4_scaffold1337:777159..778139	15:27132388..27133368
OR118-1	DQ306087		Dr3OR15.24		CN022514	full-length	gene	0	939	ctg26:617692..618630	Zv4_scaffold1337:781676..782614	15:27136905..27137843
OR118-2	DQ306089		Dr3OR15.27			full-length	gene	0	939	ctg26:627850..628788	Zv4_scaffold1337:791834..792772	15:27147063..27148001
OR118-3	DQ306088		Dr3OR15.26			full-length	gene	0	942	ctg26:623160..624101	Zv4_scaffold1337:787144..788085	15:27142373..27143314
OR119-1	DQ306090		Dr3OR15.28		CN022514	full-length	gene	0	945	ctg26:632860..633804	Zv4_scaffold1337:796844..797788	15:27152073..27153017
OR119-2	DQ306091	4.1[c]	Dr3OR15.29	NM_131751		full-length	gene	0	945	ctg26:637953..638897	Zv4_scaffold1337:801937..802881	15:27157166..27158110
OR120-1	DQ306022				CO918338	full-length	gene	0	990	NA266:6563..7552	Zv4_scaffold1202:2598425..2599414	14:3375277..3376266
OR121-1	DQ306074		Dr3OR17.2			full-length	gene	0	939	ctg13685:19469..20407	Zv4_scaffold336:1956840..1957778	Unknown
OR121-2P	DQ306075		Dr3OR17.3			partial	pseudogene	1	893	ctg13685:20657..21549	Zv4_scaffold336:1955699..1956591	Unknown
OR122-1	DQ306073		Dr3OR17.1			full-length	gene	0	924	ctg13685:11577..12547	Zv4_scaffold336:1964462..1965432	Unknown
OR122-2	DQ306023	ZOR2[d]		U72684		full-length	gene	0	924	NA266:13132..14114	Zv4_scaffold1202:2592134..2593116	14:3368986..3369968
OR123-1	DQ306120		Dr3OR21.6			full-length	gene	0	936	ctg30187:265795..266730	Zv4_scaffold1754:174451..175386	21:14030469..14031404
OR124-1	DQ306119		Dr3OR21.5			full-length	gene	0	927	ctg30187:254003..254929	Zv4_scaffold1754:162659..163585	21:14018677..14019603
OR124-2	DQ306118		Dr3OR21.3			full-length	gene	0	933	ctg30187:249538..250470	Zv4_scaffold1754:158194..159126	21:14014212..14015144
OR124-3	DQ305990		Dr3OR21.4			full-length	gene	0	933	CR450844:107765..108697	Unknown	Unknown
OR124-4	DQ305992		Dr3OR21.1			full-length	gene	0	942	CR450844:119254..120195	Zv4_scaffold1754:149639..150580	21:14005657..14006598
OR124-5P	DQ305991		Dr3OR21.2			partial	pseudogene	3	833	CR450844:117521..118353	Zv4_scaffold1754:151490..152321	21:14007508..14008339
OR125-1	DQ306121		Dr3OR21.7			full-length	gene	0	960	ctg30187:269969..270928	Zv4_scaffold1754:178625..179584	21:14034643..14035602
OR125-2	DQ306122		Dr3OR21.8			full-length	gene	0	921	ctg30187:276567..277487	Zv4_scaffold1754:185223..186143	21:14041241..14042161
OR125-3	DQ306123		Dr3OR21.10			full-length	gene	0	930	ctg30187:286886..287815	Zv4_scaffold1754:195542..196471	21:14051560..14052489
OR125-4	DQ306124		Dr3OR21.11			full-length	gene	0	924	ctg30187:293934..294857	Zv4_scaffold1754:202590..203513	21:14058608..14059531
OR125-5	DQ306125		Dr3OR21.12			full-length	gene	0	927	ctg30187:300111..301037	Zv4_scaffold1754:208767..209693	21:14064785..14065711
OR125-6	DQ306126		Dr3OR21.14			full-length	gene	0	954	ctg30187:305680..306633	Zv4_scaffold1754:214336..215289	21:14070354..14071307
OR125-7	DQ306127		Dr3OR21.15		CO935064	full-length	gene	0	936	ctg30187:316124..317059	Zv4_scaffold1754:224780..225715	21:14080798..14081733
OR125-8	DQ306128		Dr3OR21.16		AW077101<br>AW076855	full-length	gene	0	1026	ctg30187:323370..324395	Zv4_scaffold1754:232026..233051	21:14088044..14089069

Table S1. The zebrafish OR repertoire

OR126-1	DQ306057		Dr3OR15.2			full-length	gene	0	930	ctg12310:23393..24322	Zv4_scaffold1298:23393..24322	15:2947562..2948491
OR126-2	DQ306067		Dr3OR15.12			full-length	gene	0	936	ctg12310:83968..84903	Zv4_scaffold1298:83968..84903	15:3008137..3009072
OR126-3	DQ306068		Dr3OR15.13			full-length	gene	0	948	ctg12310:91460..92407	Zv4_scaffold1298:91460..92407	15:3015629..3016576
OR126-4	DQ306069		Dr3OR15.14			full-length	gene	0	945	ctg12310:96745..97689	Zv4_scaffold1298:96745..97689	15:3020914..3021858
OR126-5	DQ306070		Dr3OR15.15			full-length	gene	0	948	ctg12310:103365..104312	Zv4_scaffold1298:103365..104312	15:3027534..3028481
OR126-7	DQ306071		Dr3OR15.16			partial	gene	0	930	ctg12310:110490..111419	Zv4_scaffold1298:110490..111419	15:3034659..3035588
OR127-1	DQ306056		Dr3OR7.1			full-length	gene	0	945	ctg12310:12687..13631	Zv4_scaffold1298:12687..13631	15:2936856..2937800
OR128-1	DQ306058		Dr3OR15.3			full-length	gene	0	918	ctg12310:30049..30966	Zv4_scaffold1298:30049..30966	15:2954218..2955135
OR128-2	DQ306059		Dr3OR15.4			full-length	gene	0	918	ctg12310:36291..37208	Zv4_scaffold1298:36291..37208	15:2960460..2961377
OR128-3	DQ306060		Dr3OR15.5			full-length	gene	0	912	ctg12310:45376..46287	Zv4_scaffold1298:45376..46287	15:2969545..2970456
OR128-4	DQ306061		Dr3OR15.6			full-length	gene	0	918	ctg12310:52878..53795	Zv4_scaffold1298:52878..53795	15:2977047..2977964
OR128-5	DQ306062	ZOR3[d]	Dr3OR15.7	U72685	CO811737	full-length	gene	0	921	ctg12310:58148..59068	Zv4_scaffold1298:58148..59068	15:2982317..2983237
OR128-6	DQ306063		Dr3OR15.8			full-length	gene	0	918	ctg12310:62773..63690	Zv4_scaffold1298:62773..63690	15:2986942..2987859
OR128-7	DQ306064		Dr3OR15.9			full-length	gene	0	918	ctg12310:67887..68804	Zv4_scaffold1298:67887..68804	15:2992056..2992973
OR128-8	DQ306065		Dr3OR15.10			full-length	gene	0	933	ctg12310:72308..73240	Zv4_scaffold1298:72308..73240	15:2996477..2997409
OR128-9	DQ306066		Dr3OR15.11			full-length	gene	0	918	ctg12310:77484..78401	Zv4_scaffold1298:77484..78401	15:3001653..3002570
OR128-10	DQ306072		Dr3OR15.17			full-length	gene	0	918	ctg12310:118105..119022	Zv4_scaffold1298:118105..119022	15:3042274..3043191
OR128-11	DQ306117					partial	gene	0	803	ctg26417:527381..528183	Unknown	Unknown
OR128-12	DQ306024					partial	gene	0	664	NA3759:1879..2542	Zv4_NA18305:1879..2542	Unknown
OR128-13	DQ306025					full-length	gene	1	917	NA3759:5326..6242	Zv4_scaffold1202:5555006..5555922	14:6331858..6332774
*OR128-14**					CO927854							
OR129-1	DQ306078					full-length	gene	0	948	ctg23107:3279..4226	Zv4_scaffold1309:726151..727098	15:9606636..9607583
OR130-1	DQ306145		Dr3OR13.1			full-length	gene	0	948	ctg9441:562507..563454	Zv4_scaffold303:151346..152293	4:9370252..9371199
OR131-1	DQ306115		Dr3OR15.18			full-length	gene	0	966	ctg26293:212996..213961	Zv4_NA9866:23192..24157	Unknown
OR131-2	DQ306116		Dr3OR15.20			full-length	gene	0	981	ctg26293:228709..229689	Zv4_NA9866:38751..39731	Unknown
OR132-1	DQ306140		Dr3OR8.3			full-length	gene	0	972	ctg626:280661..281632	Zv4_scaffold1759:576909..577880	21:16037286..16038257
OR132-2	DQ306141		Dr3OR8.4			full-length	gene	0	969	ctg626:293082..294050	Zv4_scaffold1759:564491..565459	21:16024868..16025836
OR132-3	DQ306142		Dr3OR8.5			full-length	gene	0	969	ctg626:301578..302546	Zv4_scaffold1759:556296..557264	21:16016673..16017641
OR132-4	DQ306143		Dr3OR8.6			full-length	gene	0	969	ctg626:312165..313133	Zv4_scaffold1759:545800..546768	21:16006177..16007145
OR132-5	DQ306026					full-length	gene	0	969	Zv4_NA13608:168926..169894	Zv4_NA13608:168926..169894	Unknown
OR133-1	DQ306129		Dr3OR21.19			full-length	gene	0	963	ctg30187:379740..380702	Zv4_scaffold1754:288396..289358	21:14144414..14145376
OR133-2	DQ306130		Dr3OR21.20			full-length	gene	1	930	ctg30187:385596..386525	Zv4_scaffold1754:294252..295181	21:14150270..14151199
OR133-3	DQ306131		Dr3OR2.1			full-length	gene	0	948	ctg30187:400224..401171	Zv4_scaffold1754:308880..309827	21:14164898..14165845
OR133-4	DQ306132		Dr3OR21.22			full-length	gene	0	954	ctg30187:410288..411241	Zv4_scaffold1754:318944..319897	21:14174962..14175915
OR133-5	DQ306134		Dr3OR21.24			full-length	gene	0	966	ctg30187:425349..426314	Zv4_scaffold1754:334005..334970	21:14190023..14190988
OR133-6	DQ306135		Dr3OR21.25			full-length	gene	0	954	ctg30187:430427..431380	Zv4_scaffold1754:339083..340036	21:14195101..14196054
OR133-7	DQ306136		Dr3OR21.26			full-length	gene	0	951	ctg30187:437711..438661	Zv4_scaffold1758:1845..2795	21:13646298..13647248
OR133-8	DQ306137		Dr3OR21.28			full-length	gene	0	951	ctg30187:546525..547475	Zv4_NA18046:37665..38615	Unknown
OR133-9	DQ306138		Dr3OR21.29			full-length	gene	0	957	ctg30187:553092..554048	Zv4_NA18046:44232..45188	Unknown
OR133-10	DQ306133		Dr3OR21.23			partial	gene	0	813	ctg30187:416193..417105	Zv4_scaffold1754:324849..325761	21:14180867..14181779
OR134-1	DQ306048					full-length	gene	0	948	ctg12183:126793..127740	Zv4_NA16015:35286..36233	Unknown
OR135-1	DQ306077		Dr3OR8.2			full-length	gene	0	921	ctg22761:1702936..1703856	Zv4_scaffold737:1249245..1250165	8:30959750..30960670
OR135-2	DQ306076		Dr3OR8.1			full-length	gene	1	933	ctg22761:1689991..1690923	Zv4_scaffold737:1236300..1237232	8:30946805..30947737
OR136-1	DQ306047					full-length	gene	0	954	ctg12183:69987..70940	Zv4_scaffold861:714798..715751	10:4458326..4459279
OR136-2P	DQ306046					full-length	pseudogene	2	925	ctg10290:223670..224594	Zv4_scaffold1851:148800..149724	Unknown
OR137-1	DQ306044		Dr3OR7.3			full-length	gene	0	933	ctg10290:204064..204996	Zv4_scaffold1851:167877..168809	Unknown
OR137-2	DQ306045		Dr3OR7.4			full-length	gene	0	945	ctg10290:209663..210607	Zv4_scaffold1851:162571..163515	Unknown
OR137-3	DQ306051					full-length	gene	0	933	ctg12183:141392..142324	Zv4_NA16015:20756..21688	Unknown
OR137-4	DQ306053					full-length	gene	0	933	ctg12183:151806..152738	Zv4_NA16015:11182..12114	Unknown
OR137-5	DQ306055					full-length	gene	0	933	ctg12183:162936..163868	Zv4_NA16015:52..984	Unknown
OR137-6P	DQ306042					partial	pseudogene	2	836	ctg10290:184071..184906	Zv4_scaffold1851:186800..187635	Unknown
OR137-7	DQ306043				CO810666	full-length	gene	1	929	ctg10290:190253..191181	Zv4_scaffold1851:180525..181453	Unknown
OR137-8	DQ306052					full-length	gene	1	932	ctg12183:148100..149031	Zv4_NA16015:14878..15809	Unknown
OR137-9	DQ306054					partial	gene	0	486	ctg12183:160719..161204	Zv4_NA16015:2716..3201	Unknown
OR138-1P	DQ306027					partial	pseudogene	2	909	Zv4_NA14809:3451..4359	Zv4_NA14809:3451..4359	Unknown
OR139-1P	DQ306049					partial	pseudogene	2	915	ctg12183:133587..134501	Zv4_NA16015:28525..29439	Unknown
OR140-1	DQ306050					full-length	pseudogene	4	939	ctg12183:136912..137850	Zv4_NA16015:25176..26114	Unknown

[a](Barth et al., 1996), [b](Barth et al., 1997), [c](Dugas and Ngai, 2001), [d](Weth et al., 1996), [e](Byrd et al., 1996), [f](Niimura and Nei, 2005)

*Not identified in this study
