# Supplementary material for: The odorant receptor repertoire of teleost fish
Source: BMC Genomics. 2005 Dec 6;6:173. doi: 10.1186/1471-2164-6-173 (PMC1325023; doi:10.1186/1471-2164-6-173)
Supplement: Additional File 11 — Table S2. Pairwise intra-subfamily percent identities for zebrafish OR subfamilies. [file 1471-2164-6-173-S11.pdf]

Table S2. Pairwise intra-subfamily percent identities for zebrafish OR subfamilies.

Subfamily	Average	Min	Max
OR102	63	44	95
OR103	75	62	96
OR104	86	86	86
OR106	79	69	97
OR108	78	73	87
OR109	76	69	100
OR110	80	80	80
OR111	72	64	86
OR113	81	81	81
OR115	65	53	88
OR116	82	82	82
OR118	74	72	77
OR119	95	95	95
OR122	91	91	91
OR124	76	69	83
OR125	66	55	94
OR126	77	71	79
OR128	74	63	93
OR131	84	84	84
OR132	84	80	92
OR133	75	65	87
OR137	71	65	83
