# Supplementary material for: The odorant receptor repertoire of teleost fish
Source: BMC Genomics. 2005 Dec 6;6:173. doi: 10.1186/1471-2164-6-173 (PMC1325023; doi:10.1186/1471-2164-6-173)
Supplement: Additional File 12 — Table S3. Pairwise inter-subfamily percent identities for zebrafish OR subfamilies. [file 1471-2164-6-173-S12.pdf]

Table S3. Pairwise inter-subfamily percent identities for zebrafish OR subfamilies.

Subfamily	Average	Min	Max
OR101	37	27	44
OR102	25	17	34
OR103	29	22	38
OR104	30	23	37
OR105	28	21	38
OR106	24	16	32
OR107	29	23	34
OR108	26	19	33
OR109	29	21	40
OR110	27	22	33
OR111	28	20	37
OR112	31	24	42
OR113	28	22	39
OR114	26	18	35
OR115	28	21	35
OR116	28	22	35
OR117	27	21	32
OR118	26	20	33
OR119	28	22	38
OR120	29	22	34
OR121	28	21	33
OR122	27	22	34
OR123	27	22	32
OR124	28	20	35
OR125	27	20	34
OR126	27	20	33
OR127	26	20	32
OR128	26	18	33
OR129	17	11	22
OR130	16	11	20
OR131	17	12	22
OR132	18	12	23
OR133	18	12	24
OR134	18	13	24
OR135	18	13	23
OR136	16	12	22
OR137	17	12	23
