# Supplementary material for: The odorant receptor repertoire of teleost fish
Source: BMC Genomics. 2005 Dec 6;6:173. doi: 10.1186/1471-2164-6-173 (PMC1325023; doi:10.1186/1471-2164-6-173)
Supplement: Additional File 13 — Table S4. Pairwise inter-group percent identities for zebrafish OR families and mouse Class I and Class II ORs. [file 1471-2164-6-173-S13.pdf]

**Table S4. Pairwise inter-group percent identities for zebrafish OR families and mouse Class I and Class II ORs.**

| Comparison | Average | Min | Max |
|---|---|---|---|
| Zebrafish: | | | |
| Family A x B | 29 | 28 | 31 |
| Family A x C | 28 | 22 | 35 |
| Family A x D | 28 | 20 | 34 |
| Family A x E | 28 | 22 | 32 |
| Family A x F | 28 | 24 | 33 |
| Family A x G | 24 | 22 | 27 |
| Family A x H | 18 | 12 | 22 |
| Family B x C | 33 | 26 | 38 |
| Family B x D | 29 | 25 | 33 |
| Family B x E | 26 | 23 | 29 |
| Family B x F | 28 | 24 | 33 |
| Family B x G | 28 | 27 | 29 |
| Family B x H | 18 | 15 | 21 |
| Family C x D | 29 | 23 | 38 |
| Family C x E | 26 | 20 | 31 |
| Family C x F | 27 | 20 | 32 |
| Family C x G | 28 | 25 | 33 |
| Family C x H | 17 | 11 | 21 |
| Family D x E | 26 | 19 | 34 |
| Family D x F | 27 | 22 | 33 |
| Family D x G | 27 | 23 | 35 |
| Family D x H | 18 | 14 | 24 |
| Family E x F | 31 | 26 | 38 |
| Family E x G | 25 | 22 | 31 |
| Family E x H | 18 | 13 | 25 |
| Family F x G | 26 | 21 | 31 |
| Family F x H | 20 | 14 | 26 |
| Family G x H | 17 | 13 | 21 |
| Mouse x Zebrafish: | | | |
| Class I x Family A | 33 | 24 | 42 |
| Class I x Family B | 32 | 27 | 39 |
| Class I x Family C | 29 | 21 | 38 |
| Class I x Family D | 29 | 22 | 37 |
| Class I x Family E | 28 | 20 | 34 |
| Class I x Family F | 29 | 22 | 38 |
| Class I x Family G | 25 | 19 | 32 |
| Class I x Family H | 17 | 12 | 22 |
| Class II x Family A | 28 | 18 | 36 |
| Class II x Family B | 38 | 30 | 44 |
| Class II x Family C | 28 | 17 | 37 |
| Class II x Family D | 28 | 19 | 40 |
| Class II x Family E | 27 | 18 | 35 |
| Class II x Family F | 28 | 20 | 35 |
| Class II x Family G | 24 | 16 | 31 |
| Class II x Family H | 18 | 11 | 24 |
| Mouse: | | | |
| ClassI x ClassII | 29 | 19 | 39 |
