# Supplementary material for: The odorant receptor repertoire of teleost fish
Source: BMC Genomics. 2005 Dec 6;6:173. doi: 10.1186/1471-2164-6-173 (PMC1325023; doi:10.1186/1471-2164-6-173)
Supplement: Additional File 7 — Figure S6. Phylogeny of zebrafish, fugu and tetraodon ORs using maximum likelihood analysis. [file 1471-2164-6-173-S7.pdf]

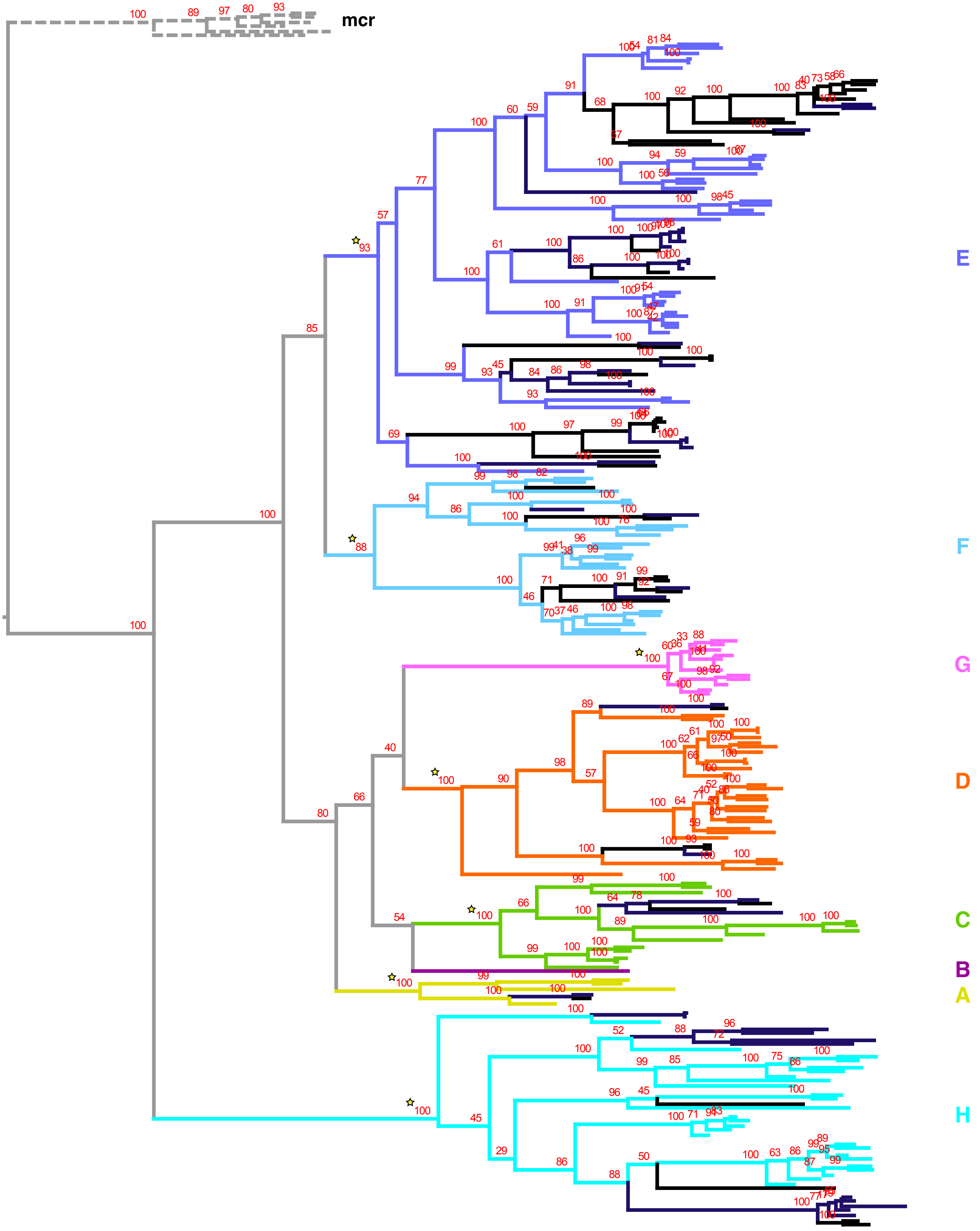

Figure S6
