# Supplementary material for: The odorant receptor repertoire of teleost fish
Source: BMC Genomics. 2005 Dec 6;6:173. doi: 10.1186/1471-2164-6-173 (PMC1325023; doi:10.1186/1471-2164-6-173)
Supplement: Additional File 8 — Figure S7. Phylogeny of zebrafish and mouse ORs rooted by mouse non-OR GPCRs. [file 1471-2164-6-173-S8.pdf]

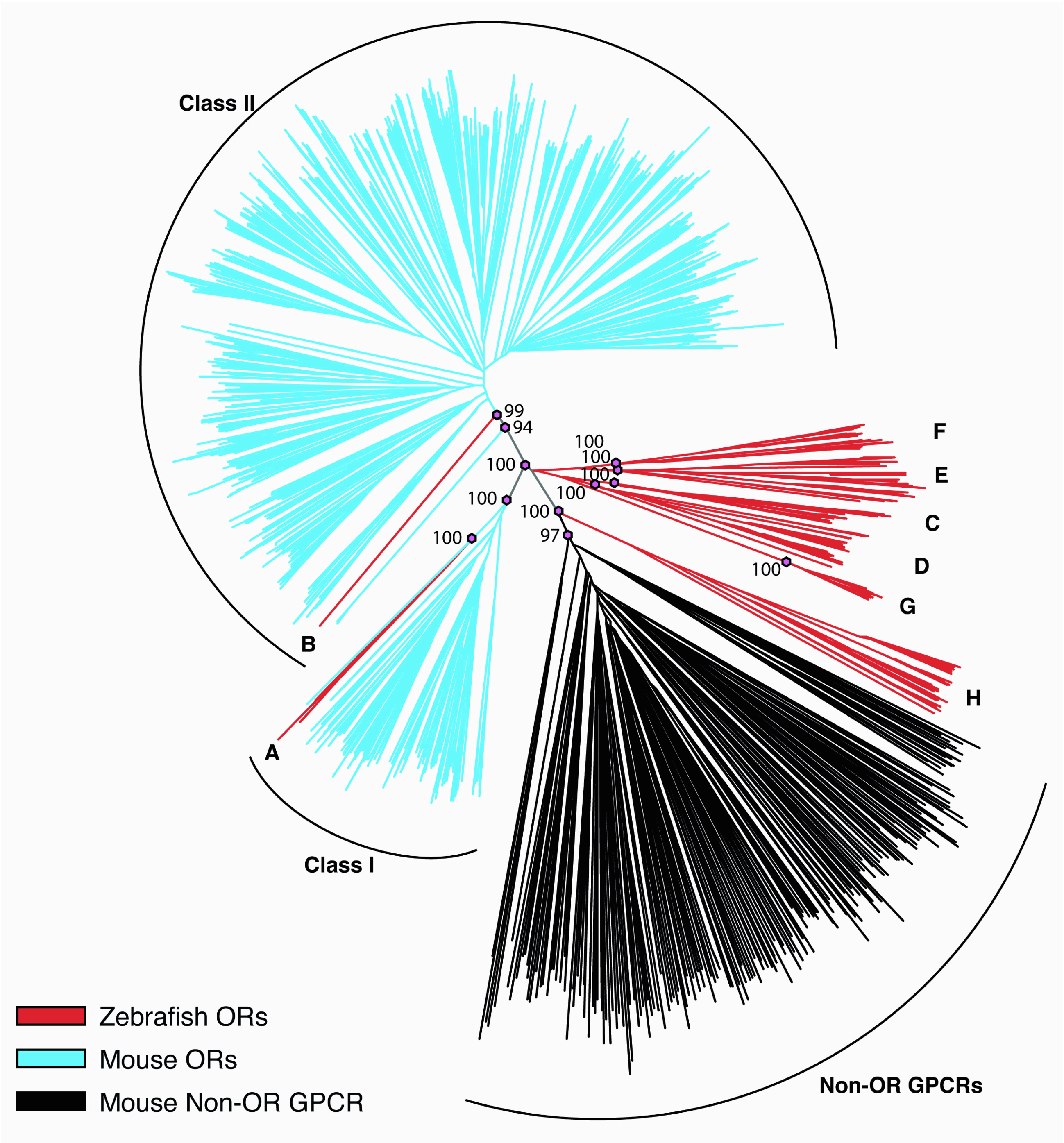
Class II
Class I
Non-OR GPCRs
A
B
C
D
E
F
G
H
99
94
100
97
Zebrafish ORs
Mouse ORs
Mouse Non-OR GPCR

Figure S7
